# Spontaneously Resolved Atopic Dermatitis Shows Melanocyte and Immune Cell Activation Distinct From Healthy Control Skin

**DOI:** 10.3389/fimmu.2021.630892

**Published:** 2021-02-24

**Authors:** Katharina Rindler, Thomas Krausgruber, Felix M. Thaler, Natalia Alkon, Christine Bangert, Harald Kurz, Nikolaus Fortelny, Thomas B. Rojahn, Constanze Jonak, Johannes Griss, Christoph Bock, Patrick M. Brunner

**Affiliations:** ^1^Department of Dermatology, Medical University of Vienna, Vienna, Austria; ^2^CeMM Research Center for Molecular Medicine of the Austrian Academy of Sciences, Vienna, Austria; ^3^Center for Medical Statistics, Informatics, and Intelligent Systems, Institute of Artificial Intelligence and Decision Support, Medical University of Vienna, Vienna, Austria

**Keywords:** atopic dermatitis, eczema, single-cell RNA seq, multiplex proteomics, spontaneous remission

## Abstract

Atopic dermatitis (AD) typically starts in infancy or early childhood, showing spontaneous remission in a subset of patients, while others develop lifelong disease. Despite an increased understanding of AD, factors guiding its natural course are only insufficiently elucidated. We thus performed suction blistering in skin of adult patients with stable, spontaneous remission from previous moderate-to-severe AD during childhood. Samples were compared to healthy controls without personal or familial history of atopy, and to chronic, active AD lesions. Skin cells and tissue fluid obtained were used for single-cell RNA sequencing and proteomic multiplex assays, respectively. We found overall cell composition and proteomic profiles of spontaneously healed AD to be comparable to healthy control skin, without upregulation of typical AD activity markers (e.g., IL13, S100As, and KRT16). Among all cell types in spontaneously healed AD, melanocytes harbored the largest numbers of differentially expressed genes in comparison to healthy controls, with upregulation of potentially anti-inflammatory markers such as PLA2G7. Conventional T-cells also showed increases in regulatory markers, and a general skewing toward a more Th1-like phenotype. By contrast, gene expression of regulatory T-cells and keratinocytes was essentially indistinguishable from healthy skin. Melanocytes and conventional T-cells might thus contribute a specific regulatory milieu in spontaneously healed AD skin.

## Introduction

Atopic dermatitis (AD) is the most common chronic inflammatory skin disease, affecting up to 20% of children and 7–10% of adults (1). The disease usually starts within the first 5 years after birth and resolves in approximately two thirds of cases until adulthood (2, 3). Despite this high rate of spontaneous resolution, severe disease can persist throughout life in a significant proportion of patients, typically showing a chronic-relapsing course, that is often recalcitrant to treatment (4). In addition, there is a risk of subsequent recurrences in resolved patients (3). Factors associated with more chronic-persistent AD are IgE autoreactivity (5, 6), allergic sensitization (7), a higher genetic risk score including *FLG* mutations (8), and high disease severity at baseline (8). However, the exact mechanisms of disease persistence are still unknown, and there is no biomarker available that can predict whether a child will outgrow the disease. Despite current attempts to prevent AD in at-risk children using intensified emollient treatment from birth on (9, 10), there is a lack of disease-modifying therapies inducing permanent remission once AD is established, as opposed to other atopic diseases such as allergic rhinoconjunctivitis or asthma, which can often be modulated by immunotherapy (11). Thus, a better understanding of mechanisms guiding the natural course of the disease is an unmet medical need.

Here, we profiled at the single-cell and proteomic levels skin from adults who suffered from severe AD during infancy and childhood, but remained in remission following spontaneous disease clearance during adolescence. In comparison to healthy control individuals, we found a distinct activation pattern in melanocytes and some T-cell subsets, while keratinocytes remained largely unaltered. These data suggest the existence of long-term immune-modifying alterations in the cellular milieu in resolved AD, which may provide the basis for developing prognostic disease parameters and biomarkers for therapy responses in AD.

## Methods

### Patients

The study was approved by the ethics committee of the Medical University of Vienna, Austria (EK1360/2018). Patients and their baseline characteristics are depicted in Table 1. Single-cell RNA sequencing (scRNA-seq, GSE153760) and proteomic multiplex data of healthy control and active AD samples were published previously by Rojahn et al. demonstrating the validity of suction blistering in comparison to full-thickness skin biopsies (12).

**Table 1:** Baseline characteristics of patients and healthy controls included for skin suction blistering.

**Sample ID**	**Age (years)**	**Sex**	**Race**	**Diagnosis**	**Atopic comorbidities**	**EASI**	**Total serum IgE (kU/L)**	**Serum specific IgE (>0.35 kU/L)**	**Sample type**	**Age of AD disease remission (years)**	**scRNA-seq**
AD20	22	Male	Caucasian	Spontaneously healed AD	Allergic rhinoconjunctivitis, asthma	0	46.2	Mugwort pollen, dog dander, C. herbarum	Suction blister	14	GSM4932358
AD21	45	Female	Caucasian	Spontaneously healed AD	Allergic rhinoconjunctivitis	0	39.7	Timothy grass, birch pollen	Suction blister	19	GSM4932359
AD24	41	Male	Caucasian	Spontaneously healed AD	Allergic rhinoconjunctivitis, asthma	0	281	Timothy grass, rye, birch, mugwort pollen, D. pteronyssinus, cat dander, dog dander	Suction blister	19	GSM4932360
AD25	41	Female	Caucasian	Spontaneously healed AD	Allergic rhinoconjunctivitis	0	428	Mugwort pollen	Suction blister	18	GSM4932361
HC1&2	42	Female	Caucasian	Healthy control	None	n.a.	n.a.	n.a.	Suction blister	n.a.	GSM4653863 GSM4653864
HC3	47	Female	Caucasian	Healthy control	None	n.a.	n.a.	n.a.	Suction blister	n.a.	GSM4653865
HC4	49	Female	Caucasian	Healthy control	None	n.a.	n.a.	n.a.	Suction blister	n.a.	GSM4653866
HC5	39	Male	Caucasian	Healthy control	None	n.a.	n.a.	n.a.	Suction blister	n.a.	GSM4653867
HC6	40	Male	Caucasian	Healthy control	None	n.a.	n.a.	n.a.	Suction blister	n.a.	n.a.
HC7	40	Male	Caucasian	Healthy control	None	n.a.	n.a.	n.a.	Suction blister	n.a.	n.a.
HC8	28	Female	Caucasian	Healthy control	None	n.a.	n.a.	n.a.	Suction blister	n.a.	n.a.
AD1	18	Male	Caucasian	Atopic dermatitis	Allergic rhinitis	34.2	>5000	D. pteronyssinus, D. farinae, plantain pollen	Suction blister	n.a.	GSM4653855
AD2	19	Female	Caucasian	Atopic dermatitis	None	44.6	8.7	None detected	Suction blister	n.a.	GSM4653856
AD3	34	Male	Caucasian	Atopic dermatitis	Allergic rhinoconjunctivitis, asthma	44.7	1,138	n.a.	Suction blister	n.a.	GSM4653857
AD4	33	Female	Caucasian	Atopic dermatitis	None	5.5	180	None detected	Suction blister	n.a.	GSM4653858

*AD, Atopic dermatitis; EASI, Eczema Area and Severity Index; HC, Healthy control; n.a., not applicable. All samples were used for multiplex proteomics; HC6-8 were not used for scRNA-seq*.

### Skin Suction Blisters

Skin suction blisters were formed with a low-pressure device from Electronic Diversities (Ridge Road, Finksburg, MD), as described previously (12, 13), using sterile 5-hole (5 mm diameter per hole) skin suction plates that were mounted onto the antecubital fossa. Samples from spontaneously healed and healthy control skin were comparable on a clinical level, without recent prolonged sun exposure, and without signs of ongoing inflammation or post-inflammatory dyspigmentation. Cell-free blister fluid was used for proteomic multiplex assays. Cells pelleted from blister fluid and the enzymatically digested blister roof (i.e., the epidermis) were subjected to FACS sorting (FACSAria III, BD Biosciences, Franklin Lakes, NJ) for the enrichment of CD45+ leukocytes, in order to also assess smaller cell fractions. Leukocytes were subsequently admixed to CD45– cells in equal amounts for best representation of all cell types, and then analyzed using scRNA-seq (10x Genomics, Pleasanton, Calif.), as previously described (12).

### Droplet-Based Single-Cell RNA-Seq

Single-cell cDNA libraries were generated using the Chromium Controller and Single Cell 3′ Library & Gel Bead Kit v2 and v3 (10x Genomics) according to the manufacturer's protocol and as previously described (12, 14). Sequencing was performed at the Biomedical Sequencing Facility (BSF), Research Center for Molecular Medicine of the Austrian Academy of Sciences (CeMM) using the Illumina HiSeq 3000/4000 platform and the 75 bp paired-end configuration.

### Preprocessing of scRNA-seq Data

Preprocessing of the scRNA-seq data was performed using Cell Ranger version 3.0.2 (10x Genomics). Raw sequencing files were demultiplexed using the Cell Ranger command “mkfastq.” Each sample was aligned to the human reference genome assembly “refdata-cellranger-GRCh38-3.0.0” using the Cell Ranger command “count.” Raw expression data were then loaded into R version 3.6.3 2020-02-29.

### Analysis of scRNA-seq

Secondary analysis of expression data was done using the Seurat package (version 3.1.4). First, cells with high mitochondrial percentage (>15%) and either very low (<200) or high numbers (>6,000) of unique genes (nFeature) were filtered out in order to achieve a population of intact, non-doublet cells to analyze. Standard integration pipeline, as recommended by the Seurat developers (15), was used to align all samples. Briefly, data were log-normalized, 2,000 variable features were selected and used for finding integration anchors, followed by scaling of the data and principal component analysis. Unsupervised clustering was done using the Louvain algorithm and a resolution of 0.6 and clusters were visualized in two-dimensional space by Uniform Manifold Approximation and Projection (UMAP) (16). Significant principal components (PCs) were picked by visual inspection and elbow plot generation and the first 16 PCs were selected as input for clustering and dimension reduction. Clusters were annotated by finding clustermarkers with the “FindAllMarkers” command and running the SingleR package (1.0.5) (17). Slight overclustering was reversed manually by joining two clusters containing melanocytes. Differential gene expression (logFCH>|0.25|, adjusted *p* < 0.05) was calculated using the FindMarkers command with logistic regression as test and “chemistry” as latent variable. *P*-values were adjusted for multiple comparisons with Bonferroni correction. 10x Genomics datasets are accessible via Gene Expression Omnibus (Table 1).

### Proteomic Quantification of Suction Blister Fluid

Suction blister fluid was analyzed by a proteomic OLINK® Proseek multiplex assay (18–20) at a dilution of 1:1 with PBS, to prevent coagulation of the sample. The following panels were used: Immune response, Immuno-oncology, Inflammation, Neuro exploratory, and Neurology, comprising 460 markers (www.olink.com). Results were obtained as log2 of NPX-values. 100% of suction blister samples passed initial QC by the manufacturer, but only samples with peak NPX expression of >2 and average NPX expression of >0.5 were finally used for analyses (*n* = 373), as markers below showed inconsistencies upon re-testing and were deemed below detection level. In case of duplicate testing, samples with a higher absolute value of log2 fold change were depicted in the graphs. The complete, unfiltered data is available in Supplementary Tables 3, 5.

## Results

### Composition of Skin Cells and Proteomic Signatures Are Comparable Between Spontaneously Healed AD and Healthy Controls

**Figure 1:**
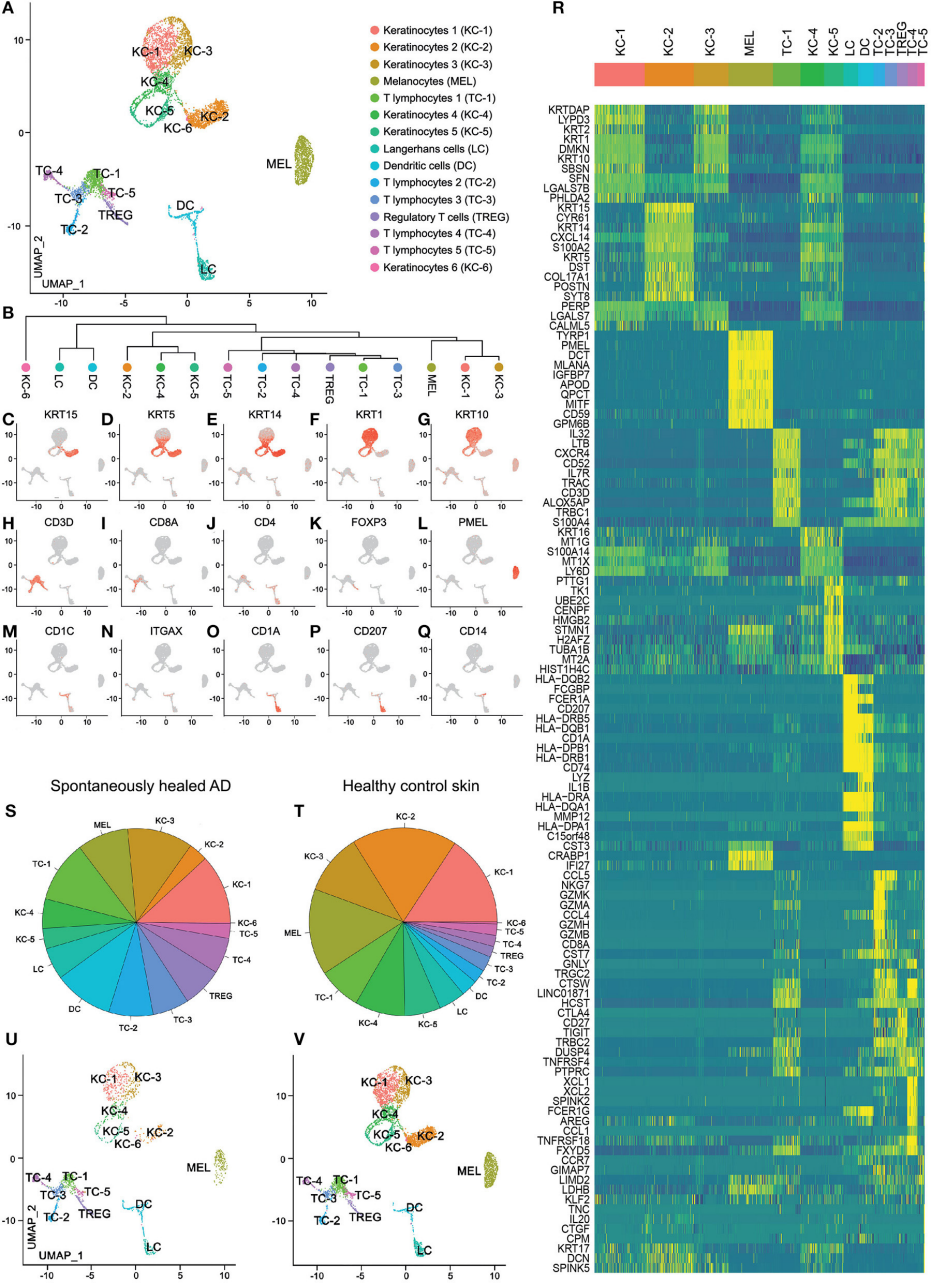
scRNA-seq map of skin cells from suction blister samples of spontaneously healed atopic dermatitis and healthy control skin. **(A)** UMAP of 7,269 cells integrated from spontaneously healed atopic dermatitis patients (*n* = 4) and healthy control individuals (*n* = 4) according to similarity of their transcriptome, resulting in 15 different color-coded clusters. **(B)** Unsupervised hierarchical clustering showing relatedness of cell clusters (average gene signatures; correlation distance metric and average linkage). **(C–Q)** Feature plots combining all samples of expression distribution for cluster-specific marker genes. Intensity of expression levels for each cell is color-coded (red) and overlaid onto UMAP plots. **(R)** Heat map displaying the top 10 differentially expressed genes (according to highest log-fold change ordered by smallest adjusted *p*-value using logistic regression with Bonferroni correction for each cluster compared with the rest of the dataset); upregulation is indicated in yellow, and downregulation in blue; gene names are shown on the left. **(S,T)** Color-coded frequencies of cell clusters for spontaneously healed atopic dermatitis (*n* = 1,849) and healthy control blister samples (*n* = 5,420) displayed as pie graphs. **(U,V)** Separate UMAP plots of spontaneously healed atopic dermatitis and healthy control blister samples. AD, atopic dermatitis; KC, Keratinocytes; MEL, Melanocytes; TC, T lymphocytes; LC, Langerhans cells; DC, Dendritic cells; TREG, regulatory T lymphocytes; UMAP, Uniform Manifold Approximation and Projection.

We investigated adult atopic patients with a history of moderate-to-severe AD starting in childhood, who outgrew their disease during their teenage years (median of disease remission: 18.5 years, see Table 1). We performed suction blistering to obtain material for proteomic (interstitial fluid) and transcriptomic analyses (cells pelleted from interstitial fluid and enzymatically digested from epidermal sheets, i.e., the blister roof) from the antecubital fossa, that has been affected by AD during childhood. As a comparator, we investigated skin from matched locations of healthy control individuals without personal or familial history of atopy (Table 1). scRNA-seq analysis was able to profile a total of 7,269 cells, 1,849 from 4 spontaneously healed AD and 5,420 from 4 healthy control individuals (Supplementary Table 1). Groups were analyzed using the Seurat toolkit in R (21, 22). Clustering followed by visualization using uniform manifold approximation and projection for dimension reduction (UMAP) (16) depicted 15 cell clusters (Figures 1A,B). Allocation of cell types to specific clusters was corroborated by canonical markers (Figures 1C–Q) and top upregulated genes (Figure 1R, Supplementary Table 2). Keratinocytes consisted of basal KC-2 (KRT15, KRT5, KRT14, POSTN, COL17A1), suprabasal KC-1 and KC-3 (KRT1, KRT10, KRT2), proliferating KC-5 (TK1, TUBA1B), activated KC-4 (KRT16), and a minor population of KC-6 expressing Tenascin-C (TNC), SPINK5, IL20 and decorin (DCN) (Figures 1C–G,R, Supplementary Table 2). The T-cell cluster contained mostly CD69+ tissue-resident memory cells (Supplementary Figure 1A), with mutually exclusive populations of CD3D+CD8+ cytotoxic (TC-2, TC-3) and CD3D+CD4+ helper T-cells (TC-1, TC-5), including FOXP3+ regulatory T-cells (TREG) (Figures 1H–K). CD3D–, CD4–, CD8–, NKG7+, KLRB1+, XCL1+, XCL2+, TNFRSF18/GITR+, FCER1G+, GNLY+, TRDC+, GZMB+, and NCAM1/CD56+ TC-4 were identified as NK-cells (TC-4), and CD3D+ NKG7+ cells as NKT cells (adjacent to and thus clustered together with TC-2) (Figures 1H–J,R and Supplementary Figures 1B–K). TC-5 expressed CCR7 and SELL/L-Selectin (Supplementary Figures 1L,M) and were thus deemed central memory T-cells (23). Myeloid cells were characterized as CD207+, CD1A+ Langerhans cells (LC) and CD1C+, ITGAX/CD11c+, LYZ+, IL1B+ dendritic cells (DC) (Figures 1M–R). All these populations were found in both healthy control and spontaneously healed samples (Figures 1S–V), and were consistently present across individual donors (Supplementary Figure 2). Consistently, we did not detect significant proteomic differences between spontaneously healed AD and healthy control samples in blister fluid, as detected by a proteomic multiplex assay (Supplementary Table 3). These data suggest that the composition of individual cell types, as well as soluble proteins are comparable between spontaneously healed AD and healthy control skin.

### On a Transcriptomic Level, Melanocytes and Conventional T-Cells From Spontaneously Healed AD Showed Largest Numbers of Differentially Expressed Genes in Comparison to Healthy Control Skin

**Figure 2:**
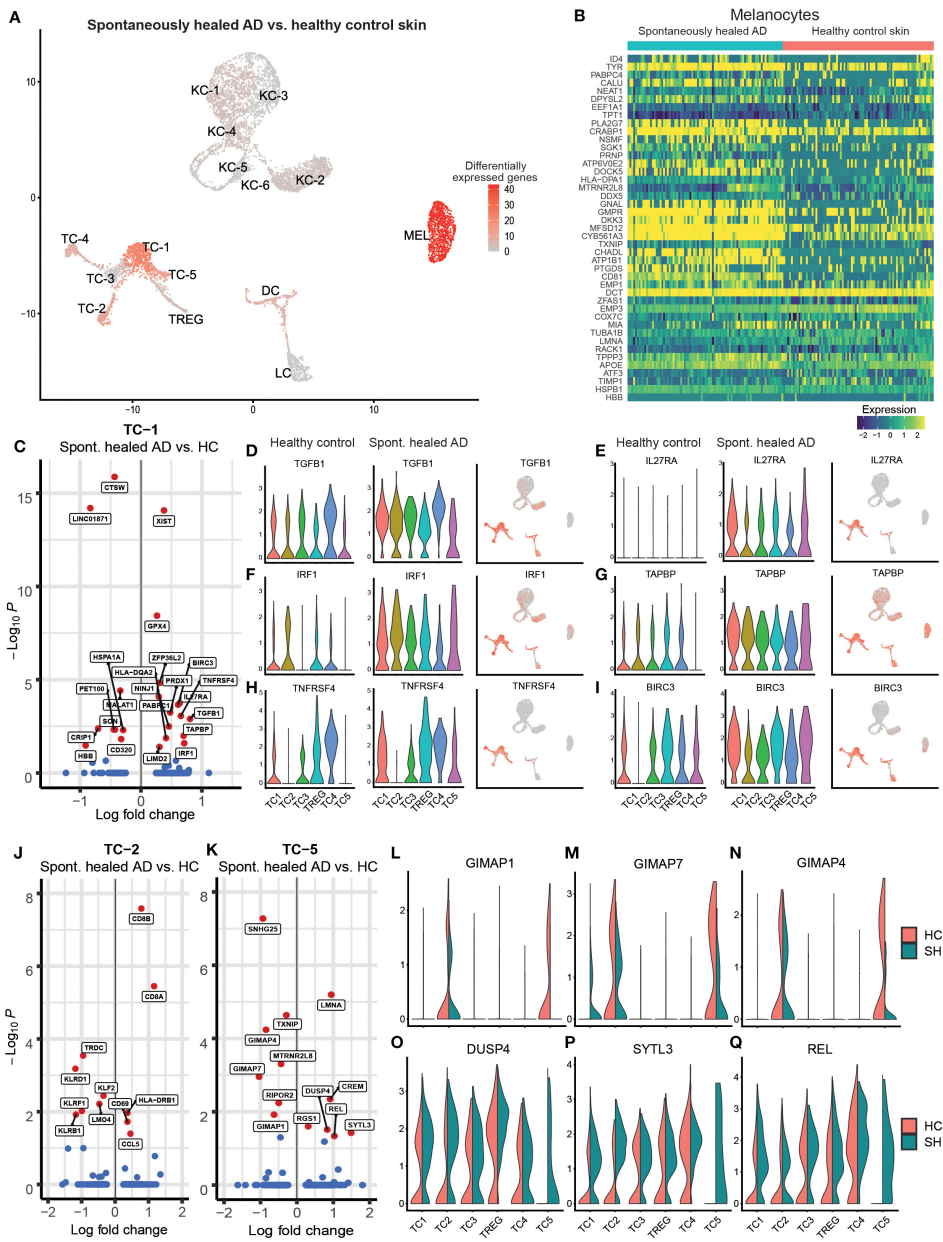
Melanocytes and T-cells show highest numbers of regulated genes in spontaneously healed atopic dermatitis in comparison to healthy control skin. **(A)** Number of differentially expressed genes (DEGs) within each cluster comparing spontaneously healed AD with healthy control samples, projected onto UMAP plots. Differential gene expression was defined as log fold change >|0.25| and adjusted *p* < 0.05 as calculated by logistic regression and Bonferroni correction. **(B)** Expression heat map of genes differentially expressed in melanocytes of spontaneously healed AD compared to healthy controls. Upregulation is indicated in yellow, downregulation in blue. Cell numbers in this heat map were down-sampled to depict equal numbers of cells between groups. **(C)** Volcano plot of differentially expressed genes in cells of the TC-1 cluster of spontaneously healed atopic dermatitis compared to healthy control samples depicted as log fold change (FCH), with a cut-off>|0.25|. Genes with a –log10 adjusted *p* > 1.3 (corresponding to an adjusted *p* < 0.05) are labeled in red. **(D–I)** Violin plots sorted per sample group within T-cell clusters showing distribution of normalized gene expression levels of the respective genes, as well as feature plots (right) combining all samples, with intensity of expression levels for each cell color-coded (red), overlaid onto UMAP plots. **(J,K)** Volcano plot of differentially expressed genes in cells of the TC-2 and the TC-5 cluster of spontaneously healed atopic dermatitis compared to healthy control samples depicted as log fold change (FCH), with a cut-off>|0.25|. Genes with a –log10 adjusted *p* > 1.3 (corresponding to an adjusted *p* < 0.05) are labeled in red. **(L–Q)** Violin plots of T-cell clusters showing distribution of normalized gene expression levels of the respective genes in healthy control (red) and spontaneously healed atopic dermatitis (green). AD, atopic dermatitis; DEGs, differentially expressed genes; HC, healthy control; SH, spontaneously healed; FCH, fold change; KC, Keratinocytes; MEL, Melanocytes; TC, T lymphocytes; LC, Langerhans cells; DC, Dendritic cells; TREG, regulatory T lymphocytes; UMAP, Uniform Manifold Approximation and Projection.

When comparing single-cell transcriptomics of cell clusters between spontaneously healed AD and healthy control skin, we only found 131 genes to be differentially expressed at a log fold change of >|0.25| and an adjusted *p* < 0.05 (Supplementary Table 4). Gene expression of keratinocytes and myeloid cells (DC, LC) was largely comparable between groups, while melanocytes and T-cell clusters TC-1, TC-2, and TC-5 showed marked differences (Figure 2A, Supplementary Table 4). Upregulated melanocyte genes (Figure 2B) included pigmentation-associated markers such as TYR and MFSD12 (24), the WNT signaling pathway inhibitor DKK3, the tetraspanin family member CD81 that can facilitate TGF-β signaling in melanocytes together with CD9 (Supplementary Figure 1N) (25), and PLA2G7 (Lipoprotein-associated phospholipase A2 or “platelet-activating factor acetylhydrolase”) catalyzing the degradation of the strongly pro-inflammatory phospholipid mediator platelet-activating factor (PAF) (26). In the helper-T-cell cluster TC-1 (Figure 2C), top upregulated genes included the regulatory cytokine TGFB1, which also showed a trend of upregulation in most other T-cell clusters (Figure 2D). We also found elevated levels of IL27RA, the receptor for the IL-12 cytokine family member IL-27 (Figure 2E), a cytokine involved in Th1 priming of T-cells (27). In line, we found significantly elevated levels of the Th1-associated (28, 29) transcription factor Interferon Regulatory Factor-1 (IRF1) in TC-1 and a general trend of elevated IRF1 levels in all T-cell subclusters (Figure 2F, Supplementary Table 4). Other upregulated markers included the MHC-associated TAPBP (tapasin) and the secondary co-stimulatory immune checkpoint molecule TNFRSF4 (CD134 or OX40), an activation marker of T-cells and anti-apoptotic receptor (30) (Figures 2G,H). In line, we also found significantly increased levels of the anti-apoptotic IAP family member BIRC3 in TC-1 cells (Figure 2I). Markers downregulated in TC-1 included Cathepsin W (CTSW), previously associated with autoimmune atrophic gastritis (31), and the transcobalamin receptor CD320 (Figure 2C). Cytotoxic T-cells in the TC-2 cluster showed upregulation of the chemokine CCL5 (RANTES) but downregulation of several killer cell lectin-like receptor genes including KLRD1 (CD94), KLRF1, and KLRB1 (CD161) (Figure 2J). Central memory T helper cells in TC-5 were characterized by downregulation of the GTPase immune-associated proteins (Gimap) genes GIMAP7, GIMAP4 and GIMAP1 (Figures 2K–N), small GTP-binding molecules defined by a common “AIG” GTP-binding domain (32), that are preferentially expressed in hematopoietic and lymphoid cells, and are associated with immune cell development and survival. We also found upregulation of dual specificity protein phosphatase 4 (DUSP4), a known inhibitor of mitogen-activated protein (MAP) kinases, thereby inhibiting cellular proliferation and differentiation (Figure 2O). TC-5 also showed elevated levels of the proto-oncogene REL/c-Rel (Figure 2P), a member of the NF-κB family of transcription factors, which is associated with Th1 skewing (33), as well as enhanced Synaptotagmin Like 3 (SYTL3) expression (Figure 2Q), belonging to a family of peripheral membrane proteins involved in vesicular trafficking. Despite a proposed key role of regulator T-cells (TREG) in controlling human autoimmune and/or auto-inflammatory skin diseases including AD (34, 35), their gene expression was essentially similar between spontaneously healed and healthy control skin (Supplementary Table 4). Thus, helper T-cells from the TC-1 and TC-5 cluster, rather than TREG, showed significant increases in regulatory as well as type-1-associated immune markers in spontaneously resolved AD when compared to healthy controls.

### Type 1-Associated Inflammation in Spontaneously Healed AD Is Increased Even Beyond Levels in Active, Chronic AD

**Figure 3:**
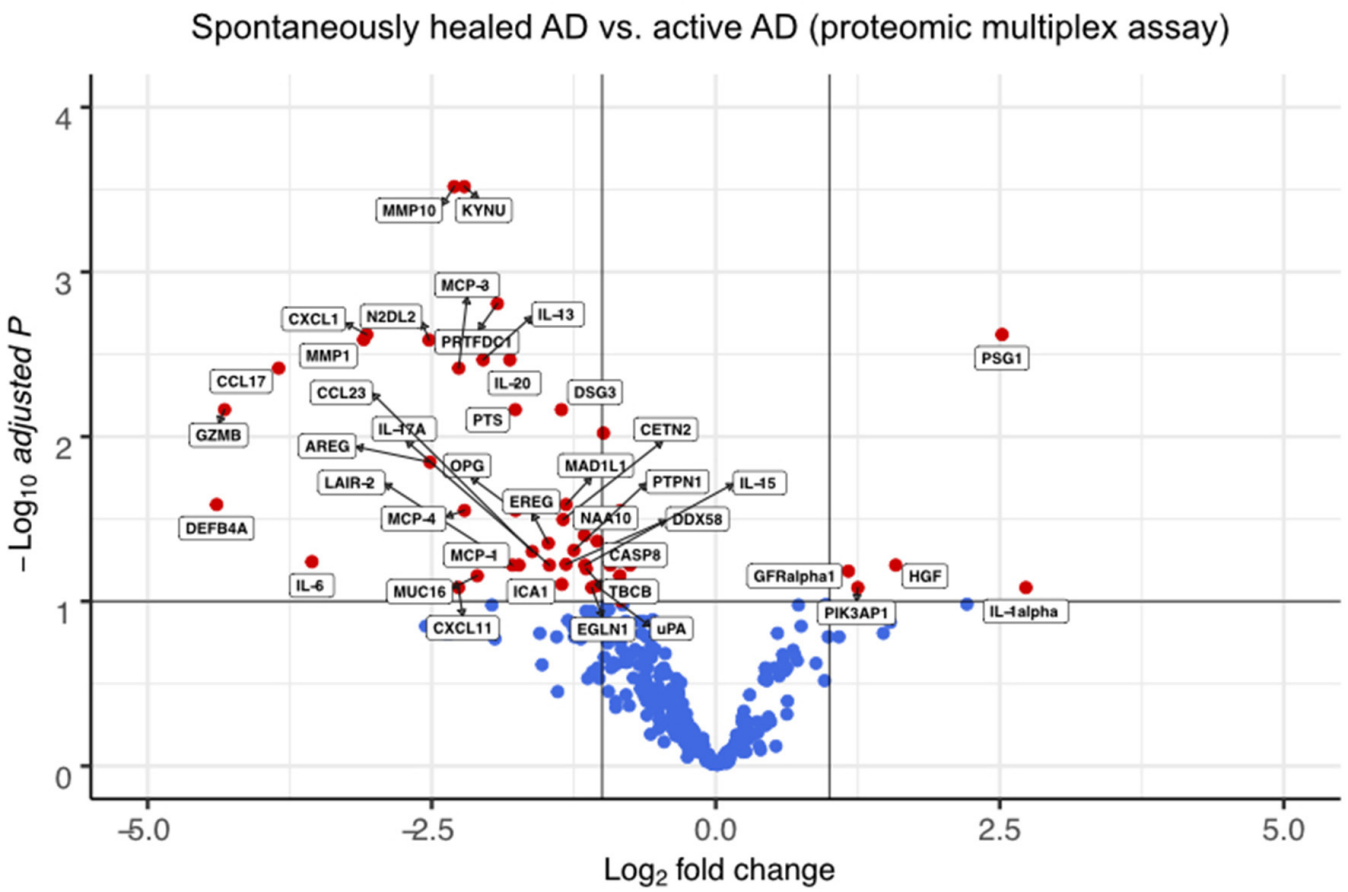
Multiplex proteomics identify markers of spontaneously healed AD. Volcano plots of proteins detected by proteomic multiplex assays in suction blister fluid, that are upregulated or downregulated in spontaneously healed atopic dermatitis (*n* = 4), depicted as log2 fold change (FCH) over active AD lesions (*n* = 4). Differences between groups were assessed using a linear mixed model (36) and Benjamini-Hochberg correction for multiple testing. Proteins with a –log10 adjusted *p* > 1 (corresponding to an adjusted *p* < 0.1) are labeled in red. AD, atopic dermatitis.

**Figure 4:**
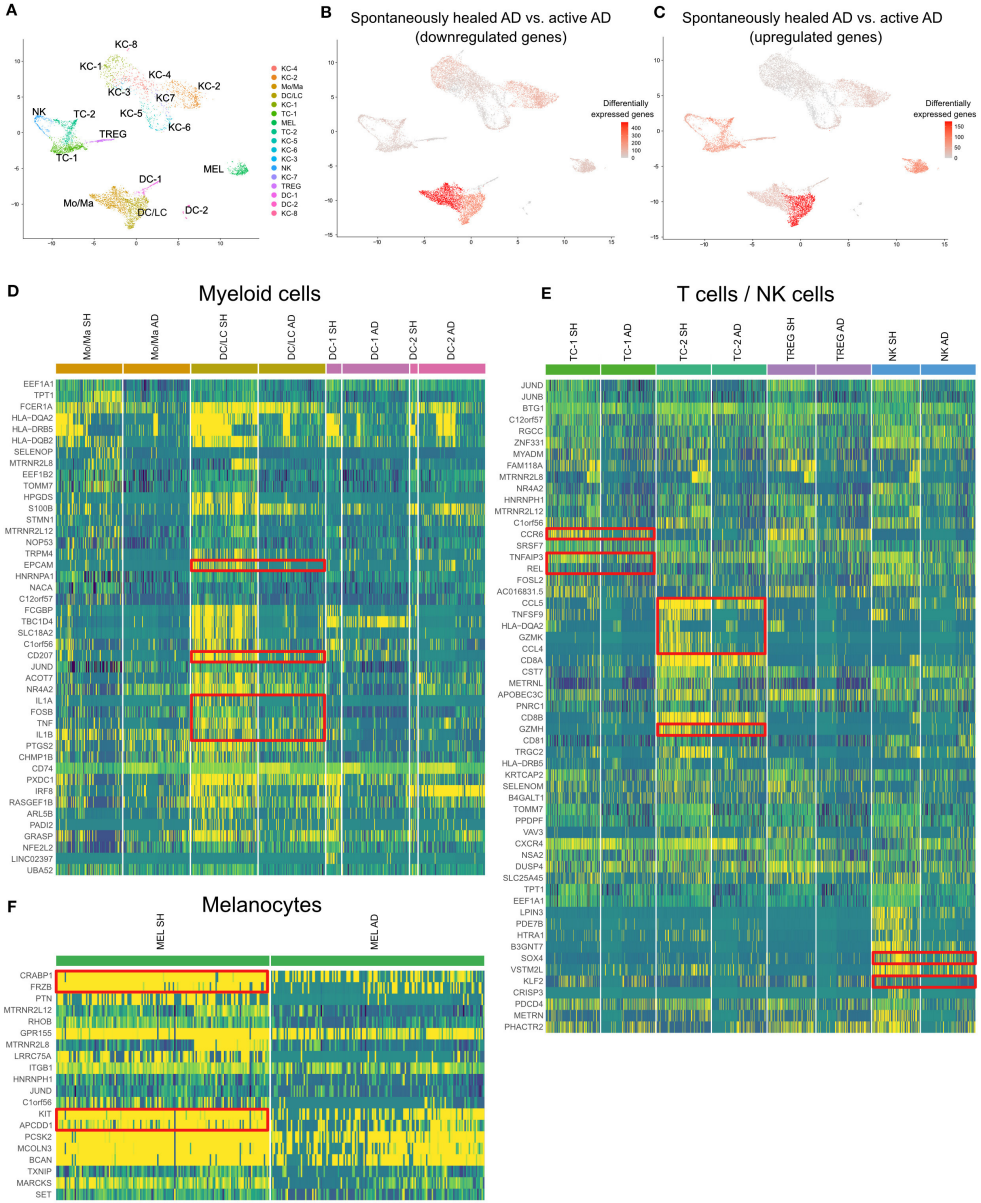
scRNA-seq map of skin cells from suction blister samples of spontaneously healed atopic dermatitis and active AD lesions. **(A)** UMAP of 14,443 cells integrated from spontaneously healed atopic dermatitis patients (*n* = 4) and active AD samples (*n* = 4) according to similarity of their transcriptome, resulting in 17 different color-coded clusters. **(B,C)** Number of differentially expressed genes (DEGs) within each cluster comparing spontaneously healed AD with active AD samples, separately for up- and downregulated DEGs, projected onto UMAP plots. Differential gene expression was defined as log fold change >|0.25| and adjusted *p* < 0.05 as calculated by logistic regression and Bonferroni correction. **(D–F)** Expression heat map of the top 20 upregulated genes in spontaneously healed AD compared to AD samples in the respective cell clusters. Upregulation is indicated in yellow, downregulation in blue. Cell numbers in heat maps were downsampled for better comparability of clusters. AD, Atopic dermatitis; KC, Keratinocytes; Mo/Ma, Monocytes/Macrophages; LC, Langerhans cells; TC, T lymphocytes; MEL, Melanocytes; NK, NK cells; TREG, regulatory T lymphocytes; DC, Dendritic cells; SH, Spontaneously healed; HC, healthy controls; UMAP, Uniform Manifold Approximation and Projection.

We also compared spontaneously healed AD with skin from 4 patients with chronic, active disease on proteomic (Figure 3, Supplementary Table 5) and transcriptomic levels (Figure 4, Supplementary Figure 3, Supplementary Table 6).

In suction blister fluid, we found broad decreases in protein levels of various pro-inflammatory cytokines, including Th2 (IL-13, CCL13/MCP-4, CCL17, CCL23), Th17 (IL-17A, CXCL1) and Th1-associated inflammation (CXCL11), and several other strongly pro-inflammatory mediators including IL-6, IL-20, CCL2/MCP-1, CCL7/MCP-3, GZMB, the T-cell/NK-cell growth factor IL-15, and the EGFR ligands AREG and EREG (Figure 3, Supplementary Table 5). By contrast, we found significant increases in the innate mediator IL-1alpha, as well as of HGF, PIK3AP1, and PSG1, markers previously associated with anti-inflammatory and immunoregulatory properties (Figure 3) (37–42). On a transcriptomic level using scRNA-seq of skin cells (Figure 4A), we observed downregulation of a variety of genes in spontaneously healed vs. active AD (Figure 4B), including typical AD activity markers such as inflammatory keratins (KRT6A, KRT16), S100A genes (S100A7, S100A8, S100A9), the EGFR ligand amphiregulin (AREG), the CCR2 and CCR4 chemokine ligand CCL2, T-cell cytotoxic molecules (GZMB, TNFSF10/TRAIL, GNLY), as well as the Th2 lead cytokine IL13 (Supplementary Figure 3C, Supplementary Table 7), consistent with proteomic data (Figure 3), and in line with clinically resolved lesions (43). Despite largely mitigated inflammation in comparison to active AD, we also found several immune mediators to be upregulated in spontaneously healed AD, especially in myeloid cells, T-cells and melanocytes (Figure 4C, Supplementary Table 7). Dendritic cells showed increased levels of the acute-phase cytokines IL1A, IL1B, and TNF, in line with more Th1 (and less Th2) skewing of spontaneously healed AD (44, 45), and increases in CD207 and EPCAM, suggestive of a shift toward higher proportions of Langerhans cells (46) (Figure 4D). Among T-cells, CD4+ helper cells (TC-1) harbored increases in the CCL20 receptor CCR6, the transcription factor REL, and TNFAIP3, a TNF-induced NF-κB inhibitor (Figure 4E, Supplementary Table 7). CD8+ T-cells (TC-2) showed increases in cytotoxic molecules GZMK, GZMH, the chemokine ligands CCL4, CCL5, as well as the TNF superfamily member TNFSF9 (CD137L), all associated with preferential type-1 T-cell polarization (47–49) (Figure 4E). NK-cells upregulated SOX4, a TGF-β-induced transcription factor previously shown to inhibit Th2 cell differentiation (50), and KLF2 (Figure 4E), implicated in NK-cell quiescence, termination of inflammation, but also NK-cell tissue survival under homeostatic conditions (51). Overall, these data demonstrate the overexpression of various Th1-associated inflammatory mediators in spontaneously healed AD, even beyond levels found in active, chronic AD. Top upregulated markers in melanocytes included the retinoid acid transporter CRABP1, the Wnt signaling antagonists Frzb and APCDD1, as well as the pigmentation-associated receptor tyrosine kinase KIT (CD117) (Figure 4F) (52).

## Discussion

This is the first cellular and molecular characterization of AD patients that spontaneously cleared their skin disease. In comparison to healthy control individuals, we found only minimal regulation in keratinocytes and myeloid cells. Among T-cells, there was an increase in Th1-associated markers such as the interferon regulatory factor IRF1, the transcription factor REL/c-Rel, and the cytokine receptor IL27RA on a transcriptomic level. IL-27 is not only involved in Th1 priming of T-cells, but also inhibits the development of Th2 and Th17 cells (27). Importantly, IL-27 receptor-deficient (IL27RA–/–) mice have been shown to develop exaggerated pro-inflammatory T-cell responses and autoimmunity (53, 54), suggesting that IL-27 to also inhibits tissue inflammation, possibly via induction of type 1 regulatory cells (Tr1) cells (55, 56). The Th1-skewing in spontaneously healed skin is consistent with previous data showing that toddlers and children suffering from AD have a delayed development of skin-homing Th1 cells (57–59). Moreover, reduced IFN-γ and enhanced IL-4 production in cord blood CD4+ T cells is associated with a higher risk to develop AD during the first 2 years after birth (60–62), suggesting that a Th1-skewed immune milieu protects against atopy early in life (63–65). Conversely, T-cells from adult patients with active AD lack significant amounts of c-Rel, failing to sufficiently translocate this transcription factor into the nucleus following activation, resulting in an impaired Th1 cytokine response, while production of Th2-associated mediators remains undisturbed (33). Thus, our data suggest that a Th1-skewed immune profile of activated T-cells (e.g., TNFRSF4/OX40+ cells in TC-1) might be involved in the sustained remission of spontaneously healed AD in adults. In line, OX40 has previously been demonstrated to control autoinflammation in a colitis model via expression on regulatory T-cells (66). However, OX40-OX40L interaction can also be involved in acceleration of Th2 responses in some settings, such as in the absence of IL-12 (67), adding to the complexity of skin immune regulation, which needs further investigation in future studies. On a proteomic level, we found the immunoregulatory markers PIK3AP1 and PSG1 to be elevated above levels of active AD. PIK3AP1 is a phosphoinositide 3-kinase (PI3K) binding protein (68) with anti-inflammatory properties on myeloid and T-cells (37, 38), as its deficiency was demonstrated to result in exaggerated innate immune responses via recruitment of inflammatory myeloid cells and inflammasome activation (39, 40). PSG1 has originally been described to be upregulated during pregnancy as an immunomodulator to protect the growing fetus, but also has regulatory properties in a murine colitis model by activating TGF-β1 and TGF-β2 (41). The concept of a decreased propensity to autoimmune disease in spontaneously healed AD was further supported by a significant increase of TGFB1 expression, the gene encoding the prototypic regulatory cytokine TGF-β (34), when comparing T-cells from spontaneously healed AD with those from healthy control skin. TGF-β, one of the most immunosuppressive cytokines known (69), is a potent inhibitor of activated T-cells and a suppressor of macrophage and monocyte activity (70). However, TGF-β functions are manifold and complex, as this cytokine also contributes to the development, long-term retention, and survival of tissue-resident memory T-cells (71–74), which are considered to be main players in chronic-recurrent inflammatory diseases (75). Surprisingly, we did not see any transcriptomic changes in regulatory T-cells, which have previously been proposed as the major cell type preventing autoimmunity, including in AD (76, 77). Thus, one might speculate that regulatory T-cells could play less of an active role at this stage of the disease, at least inferred from overall transcriptomic patterns, but this notion needs confirmation in functional experiments.

Inflammation-associated pigmentation changes are very common, including post-inflammatory leukoderma in AD or pityriasis alba in atopic individuals (78–80), but underlying mechanisms are only insufficiently understood. Generally, Napolitano et al. found fewer melanocytic nevi in atopic vs. non-atopic dermatitis children in a birth cohort study (81). On a molecular level, inflammatory cytokines synergistically inhibit growth and pigment production of melanocytes (82, 83), but also TGF-β can influence skin pigmentation via melanocyte growth (84, 85) and melanin production (84, 86). Thus, a TGF-β1-skewed environment as in our spontaneously healed samples, combined with increases in CD81 in melanocytes that facilitates TGF-β signaling (25), might have some impact on these cells, despite the fact that during the time of sampling, we did not notice overt clinical dyspigmentation in our patients. Given transcriptomic changes, melanocytes themselves might even influence the inflammatory milieu in spontaneously healed AD, as suggested by their upregulation of PLA2G7 that degrades the strongly inflammatory bioactive lipid PAF. Bioactive lipids have been identified as critical regulators of inflammation, including various atopic diseases (26). However, PAF is also secreted early after UV irradiation of skin (87), mediating immunosuppressive properties of phototherapy (88, 89), a paradox that needs further elucidation in functional experiments.

Limitations of our suction blister study are the lack of dermal cells such as mast cells, fibroblasts, endothelial cells or macrophages, which themselves might have important contributions to skin clearance in AD, and should be assessed in future studies. Also, the sample size was relatively small. Given the overall heterogeneity of AD (90), results obtained will need validation in more defined AD subsets, including individuals with or without allergic sensitization to environmental or auto-allergens (91), or depending on ethnic background or underlying mutations (92). Nevertheless, our study provides the first molecular characterization of spontaneously healed AD, highlighting several differences from healthy control skin that might help to facilitate future disease-modifying treatment strategies, which are urgently needed for patients suffering from persistent disease.

## Data Availability Statement

The datasets presented in this study can be found in online repositories. The names of the repository/repositories and accession number(s) can be found below: NCBI GEO GSE162054 and GSE153760.

## Ethics Statement

The studies involving human participants were reviewed and approved by the Ethics Committee of the Medical University of Vienna. The patients/participants provided their written informed consent to participate in this study.

## Author Contributions

PB, CBo, TK, and CBa planned this study. KR, TK, FT, NA, CBa, HK, NF, TR, CJ, and JG were involved in acquisition and processing of samples and/or analysis and interpretation of data. KR, CBa, and PB drafted and wrote the initial manuscript. All authors reviewed the final manuscript and approved its submission to Frontiers in Immunology.

## Conflict of Interest

CBa is an employee of the Medical University of Vienna, and has received personal fees from Bayer, Mylan, LEO Pharma, Pfizer, Sanofi Genzyme, Eli Lilly, Novartis, Celgene, and AbbVie. CBa is an investigator for Novartis, Sanofi, Abbvie, Elli Lilly, and Galderma (grants paid to her institution). CJ is an employee of the Medical University of Vienna, and has received personal fees from LEO Pharma, Pfizer, Eli Lilly and Company, Novartis, Takeda, Mallinckrodt/Therakos, AbbVie, Janssen, and Almirall; and is an investigator for Eli Lilly and Company, Novartis, and 4SC (grants paid to her institution). PB is an employee of the Medical University of Vienna, and has received personal fees from LEO Pharma, Pfizer, Sanofi, Eli Lilly, Novartis, Celgene, UCB Pharma, Biotest, Boehringer Ingelheim, AbbVie, Amgen and Arena Pharmaceuticals. PB is an investigator for Novartis (grants paid to his institution). The remaining authors declare that the research was conducted in the absence of any commercial or financial relationships that could be construed as a potential conflict of interest.

## Supplementary Material

Supplementary Figure 1 Feature plots of selected marker genes for the entire scRNA-seq dataset: **(A–N)** Expression levels for each cell are color-coded (red) and overlaid onto UMAP plots. Intensity of color reflects respective level of expression.

Supplementary Figure 2 scRNA-seq map of individual suction blister donors from spontaneously healed AD and healthy control individuals: UMAP plots of individual samples as used for scRNA-seq analyses. AD and HC labels correspond to sample ID of individuals listed in Table 1. AD, Atopic dermatitis; HC, Healthy control; KC, Keratinocytes; MEL, Melanocytes; TC, T lymphocytes; LC, Langerhans cells; DC, Dendritic cells; TREG, regulatory T lymphocytes; UMAP, Uniform Manifold Approximation and Projection.

Supplementary Figure 3 scRNA-seq map of skin cells from suction blister samples of spontaneously healed atopic dermatitis and active AD lesions. **(A,B)** UMAP of spontaneously healed atopic dermatitis patients (*n* = 4) and active AD samples (*n* = 4) according to similarity of their transcriptome, resulting in 17 different color-coded clusters. **(C)** Expression heat map of top 10 downregulated DEGs per cluster in spontaneously healed AD vs. active AD samples. Upregulation is indicated in yellow, downregulation in purple. AD, Atopic dermatitis; KC, Keratinocytes; Mo/Ma, Monocytes/Macrophages; LC, Langerhans cells; TC, T lymphocytes; MEL, Melanocytes; NK, NK cells; TREG, regulatory T lymphocytes; DC, Dendritic cells; UMAP, Uniform Manifold Approximation and Projection.

Supplementary Table 1 Cell numbers, read numbers, and gene numbers for each suction blister sample used for scRNA-seq. AD, Atopic dermatitis; HC, Healthy control.

Supplementary Table 2 Top 10 differentially expressed genes according to highest average log fold change (avg logFC) ordered by smallest adjusted *p*-value using logistic regression with Bonferroni correction for each cluster, as compared to the rest of the dataset in all sequenced skin cells (comparison spontaneously healed AD vs. healthy control skin). pct.1/2: The percentage of cells in which the feature is detected in the first/second group.

Supplementary Table 3 Differential protein expression as detected by a proteomic multiplex assay from suction blister fluid, given as log2 fold change (FCH) of spontaneously healed atopic dermatitis (*n* = 4) over healthy control skin (*n* = 7); adjusted *p*-values were calculated using a linear mixed model with Benjamini-Hochberg correction for multiple testing.

Supplementary Table 4 Differentially expressed genes of single-cell RNA sequencing analyses, comparing spontaneously healed atopic dermatitis with healthy control samples for each cluster. Differential gene expression was defined by average log fold change (avg logFC) >|0.25| and adjusted *p* < 0.05, using logistic regression and Bonferroni correction. pct.1/2: The percentage of cells in which the feature is detected in the first/second group.

Supplementary Table 5 Differential protein expression as detected by a proteomic multiplex assay from suction blister fluid, given as log2 fold change (logFC) of spontaneously healed atopic dermatitis (*n* = 4) over active AD (*n* = 4); adjusted p values were calculated using a linear mixed model with Benjamini-Hochberg correction for multiple testing.

Supplementary Table 6 Top 10 differentially expressed genes according to highest average log fold change (avg logFC) ordered by smallest adjusted p-value using logistic regression with Bonferroni correction for each cluster, as compared to the rest of the dataset in all sequenced skin cells (comparison spontaneously healed AD vs. chronic active AD). pct.1/2: The percentage of cells in which the feature is detected in the first/second group.

Supplementary Table 7 See separate Excel Sheet. Differentially expressed genes of single-cell RNA sequencing analyses, comparing spontaneously healed atopic dermatitis with untreated, active AD for each cluster. Differential gene expression was defined by average log fold change (avg logFC) >|0.25| and adjusted *p* < 0.05, using logistic regression and Bonferroni correction. pct.1/2: The percentage of cells in which the feature is detected in the first/second group.

## Abbreviations

AD: Atopic dermatitis
AREG: Amphiregulin
EASI: Eczema Area and Severity Index
GZMB: Granzyme B
HC: Healthy control
IRF1: Interferon regulatory factor 1
LC: Langerhans cell
NPX: Normalized protein expression
scRNA-seq: Single-cell RNA sequencing
UMAP: Uniform Manifold Approximation and Projection.
