# Supplementary figures and images for: Spontaneously Resolved Atopic Dermatitis Shows Melanocyte and Immune Cell Activation Distinct From Healthy Control Skin
Source: Front Immunol. 2021 Feb 24;12:630892. doi: 10.3389/fimmu.2021.630892 (PMC7943477; doi:10.3389/fimmu.2021.630892)

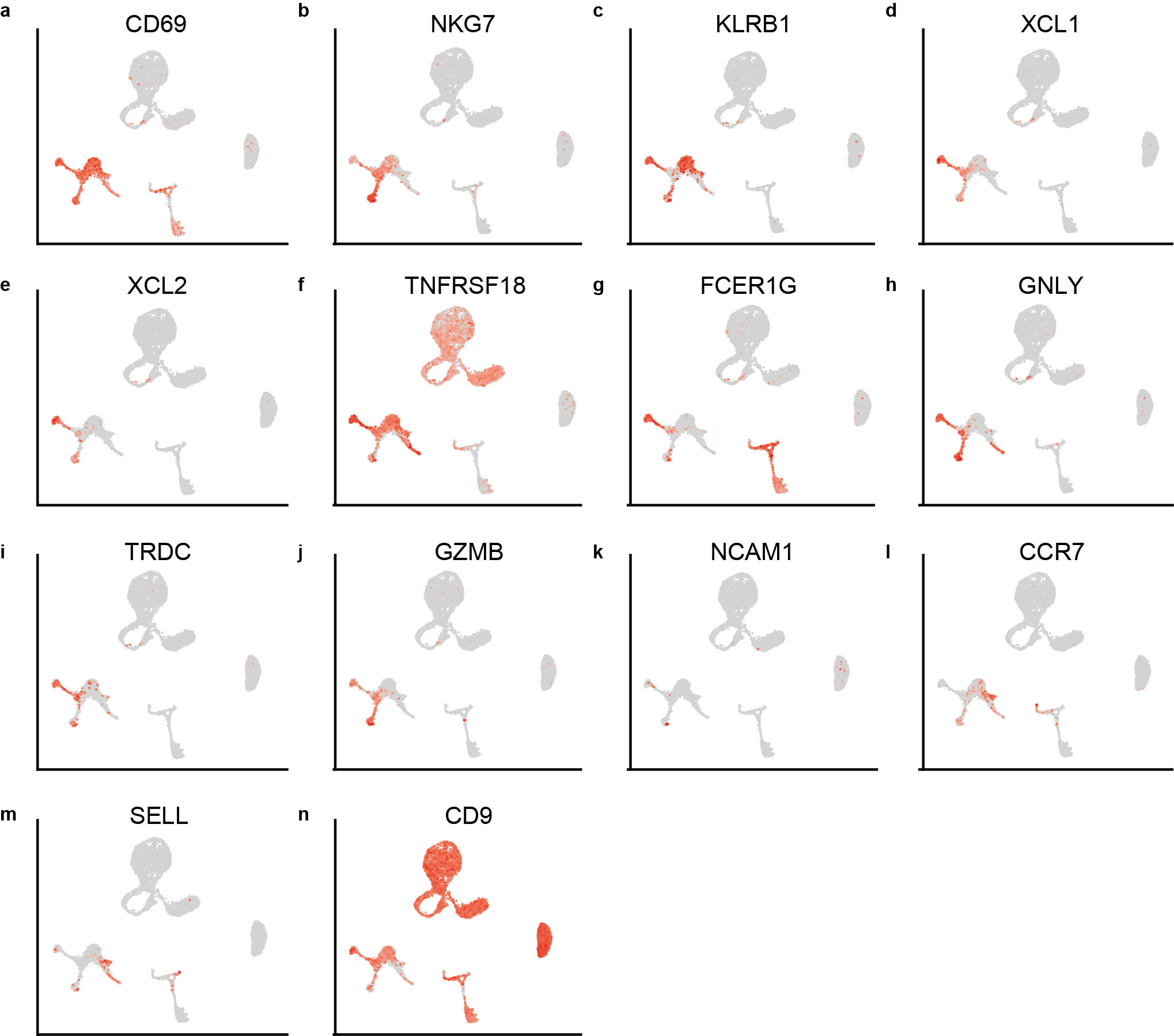

Supplement: Supplementary Figure 1 — Feature plots of selected marker genes for the entire scRNA-seq dataset: (A–N) Expression levels for each cell are color-coded (red) and overlaid onto UMAP plots. Intensity of color reflects respective level of expression. [file Image_1.JPEG]

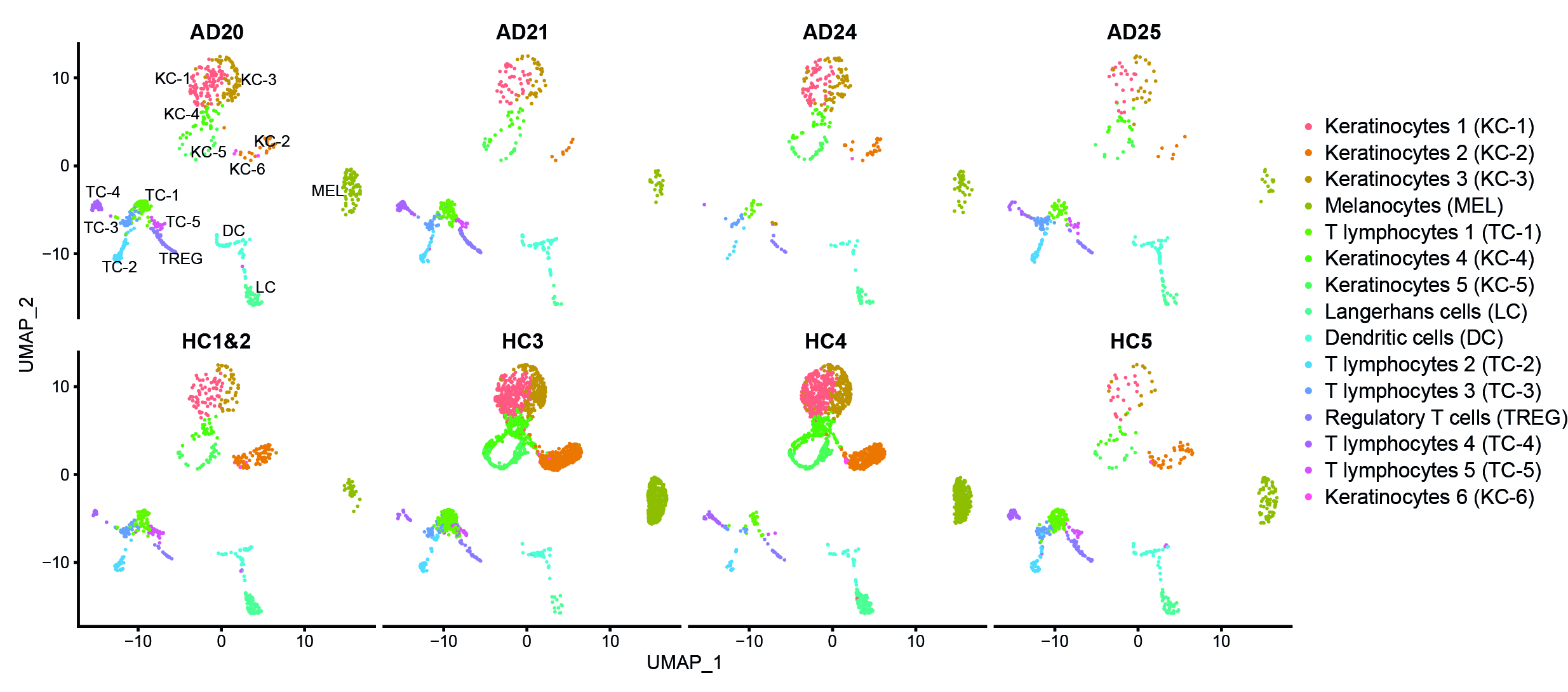

Supplement: Supplementary Figure 2 — scRNA-seq map of individual suction blister donors from spontaneously healed AD and healthy control individuals: UMAP plots of individual samples as used for scRNA-seq analyses. AD and HC labels correspond to sample ID of individuals listed in Table 1. AD, Atopic dermatitis; HC, Healthy control; KC, Keratinocytes; MEL, Melanocytes; TC, T lymphocytes; LC, Langerhans cells; DC, Dendritic cells; TREG, regulatory T lymphocytes; UMAP, Uniform Manifold Approximation and Projection. [file Image_2.JPEG]
