# Supplementary material for: Spontaneously Resolved Atopic Dermatitis Shows Melanocyte and Immune Cell Activation Distinct From Healthy Control Skin
Source: Front Immunol. 2021 Feb 24;12:630892. doi: 10.3389/fimmu.2021.630892 (PMC7943477; doi:10.3389/fimmu.2021.630892)
Supplement: Supplementary Table 1 — Cell numbers, read numbers, and gene numbers for each suction blister sample used for scRNA-seq. AD, Atopic dermatitis; HC, Healthy control. [file Data_Sheet_1.zip › Supplementary Tables 1-6 .docx]

| **Sample ID** | **Cell number** | **Read number** | **Gene number** | **Cells after filtration** | **Chemistry** |
| --- | --- | --- | --- | --- | --- |
| AD20 | 1081 | 74886 | 1691 | 705 | v3 |
| AD21 | 851 | 91969 | 1274 | 476 | v3 |
| AD24 | 860 | 83454 | 422 | 339 | v3 |
| AD25 | 806 | 92103 | 384 | 329 | v3 |
| HC1 | 171 | 509171 | 273 | 85 | v2 |
| HC2 | 751 | 125639 | 1024 | 554 | v2 |
| HC3 | 2709 | 31222 | 1206 | 2236 | v2 |
| HC4 | 2277 | 35237 | 1520 | 1983 | v2 |
| HC5 | 915 | 106691 | 1936 | 562 | v3 |
| AD1 | 4864 | 17160 | 1158 | 4104 | v2 |
| AD2 | 4104 | 16031 | 1784 | 3727 | v2 |
| AD3 | 2084 | 36272 | 1994 | 1713 | v2 |
| AD4 | 3483 | 22785 | 2179 | 3050 | v3 |

**Supplementary Table 1: Cell numbers, read numbers and gene numbers** for each suction blister sample used for scRNA-seq. AD: Atopic dermatitis; HC: Healthy control.

| **p value** | **Avg_logFC** | **pct.1** | **pct.2** | **Adjusted p value** | **Cluster** | **Gene** | **Cluster label** |
| --- | --- | --- | --- | --- | --- | --- | --- |
| 0 | 1.849647815 | 0.97 | 0.3 | 0 | 0 | KRTDAP | KC-1 |
| 0 | 1.781201819 | 0.97 | 0.33 | 0 | 0 | LYPD3 |  |
| 0 | 1.616345965 | 1 | 0.48 | 0 | 0 | KRT1 |  |
| 0 | 1.569644996 | 1 | 0.55 | 0 | 0 | DMKN |  |
| 0 | 1.534443868 | 1 | 0.75 | 0 | 0 | KRT10 |  |
| 0 | 1.434214978 | 0.84 | 0.18 | 0 | 0 | SBSN |  |
| 0 | 1.432265594 | 1 | 0.57 | 0 | 0 | SFN |  |
| 0 | 1.271270917 | 0.98 | 0.45 | 0 | 0 | LGALS7B |  |
| 2.66E-239 | 1.628678088 | 0.62 | 0.12 | 8.93E-235 | 0 | KRT2 |  |
| 1.56E-206 | 1.262054487 | 0.75 | 0.32 | 5.22E-202 | 0 | PHLDA2 |  |
| 0 | 3.095626172 | 0.97 | 0.09 | 0 | 1 | KRT15 | KC-2 |
| 0 | 2.269129359 | 0.78 | 0.12 | 0 | 1 | CYR61 |  |
| 0 | 2.219869944 | 1 | 0.56 | 0 | 1 | KRT14 |  |
| 0 | 2.179233784 | 1 | 0.46 | 0 | 1 | CXCL14 |  |
| 0 | 1.859879256 | 0.98 | 0.24 | 0 | 1 | S100A2 |  |
| 0 | 1.505560337 | 1 | 0.39 | 0 | 1 | KRT5 |  |
| 0 | 1.456050813 | 0.9 | 0.25 | 0 | 1 | DST |  |
| 0 | 1.453167594 | 0.84 | 0.14 | 0 | 1 | COL17A1 |  |
| 0 | 1.427852568 | 0.69 | 0.04 | 0 | 1 | POSTN |  |
| 0 | 1.366549217 | 0.76 | 0.14 | 0 | 1 | SYT8 |  |
| 0 | 1.529519815 | 1 | 0.51 | 0 | 2 | KRT1 | KC-3 |
| 0 | 1.269884223 | 1 | 0.57 | 0 | 2 | DMKN |  |
| 0 | 1.51446693 | 0.99 | 0.76 | 2.94E-307 | 2 | KRT10 |  |
| 5.10E-285 | 1.142201617 | 0.97 | 0.47 | 1.71E-280 | 2 | LGALS7B |  |
| 3.14E-284 | 1.105179573 | 0.99 | 0.57 | 1.05E-279 | 2 | PERP |  |
| 6.22E-280 | 1.551745641 | 0.94 | 0.33 | 2.09E-275 | 2 | KRTDAP |  |
| 3.10E-195 | 1.121842988 | 0.88 | 0.43 | 1.04E-190 | 2 | LGALS7 |  |
| 3.33E-168 | 1.104191935 | 0.74 | 0.23 | 1.12E-163 | 2 | SBSN |  |
| 3.42E-133 | 1.273177749 | 0.56 | 0.16 | 1.15E-128 | 2 | CALML5 |  |
| 8.11E-111 | 1.498715838 | 0.53 | 0.16 | 2.72E-106 | 2 | KRT2 |  |
| 0 | 4.924198459 | 1 | 0.04 | 0 | 3 | DCT | Melanocytes |
| 0 | 4.172606609 | 1 | 0.04 | 0 | 3 | MLANA |  |
| 0 | 4.016906647 | 0.98 | 0.04 | 0 | 3 | PMEL |  |
| 0 | 3.820572286 | 0.97 | 0.02 | 0 | 3 | TYRP1 |  |
| 0 | 3.645225822 | 0.99 | 0.03 | 0 | 3 | IGFBP7 |  |
| 0 | 3.454980218 | 0.99 | 0.02 | 0 | 3 | APOD |  |
| 0 | 2.895267227 | 0.94 | 0.01 | 0 | 3 | MITF |  |
| 0 | 2.87804726 | 1 | 0.41 | 0 | 3 | CD59 |  |
| 0 | 2.827293753 | 0.98 | 0.07 | 0 | 3 | QPCT |  |
| 0 | 2.699542157 | 0.97 | 0.03 | 0 | 3 | GPM6B |  |
| 0 | 1.949456333 | 1 | 0.18 | 0 | 4 | IL32 | TC-1 |
| 0 | 1.869089561 | 0.98 | 0.17 | 0 | 4 | CXCR4 |  |
| 0 | 1.721168491 | 1 | 0.26 | 0 | 4 | CD52 |  |
| 0 | 1.520842102 | 1 | 0.53 | 0 | 4 | S100A4 |  |
| 0 | 1.556423999 | 0.96 | 0.13 | 0 | 4 | CD3D |  |
| 3.84E-305 | 1.51306132 | 0.92 | 0.18 | 1.29E-300 | 4 | ALOX5AP |  |
| 6.04E-303 | 1.91846408 | 0.88 | 0.17 | 2.02E-298 | 4 | LTB |  |
| 1.18E-286 | 1.560137422 | 0.95 | 0.26 | 3.95E-282 | 4 | TRAC |  |
| 2.77E-239 | 1.581787399 | 0.75 | 0.13 | 9.28E-235 | 4 | IL7R |  |
| 1.05E-164 | 1.536579413 | 0.62 | 0.09 | 3.52E-160 | 4 | TRBC1 |  |
| 8.92E-277 | 1.541924559 | 1 | 0.44 | 2.99E-272 | 5 | KRT5 | KC-4 |
| 1.16E-230 | 1.097793003 | 0.99 | 0.46 | 3.88E-226 | 5 | S100A14 |  |
| 4.13E-186 | 1.15376907 | 0.99 | 0.59 | 1.38E-181 | 5 | KRT14 |  |
| 3.76E-182 | 0.994397492 | 1 | 0.6 | 1.26E-177 | 5 | SFN |  |
| 5.85E-166 | 1.056106693 | 0.99 | 0.65 | 1.96E-161 | 5 | MT1X |  |
| 2.74E-163 | 1.014856554 | 0.73 | 0.19 | 9.19E-159 | 5 | MT1G |  |
| 3.50E-153 | 0.866419714 | 1 | 0.49 | 1.17E-148 | 5 | LY6D |  |
| 3.90E-149 | 1.495494581 | 0.63 | 0.11 | 1.31E-144 | 5 | KRT16 |  |
| 1.34E-141 | 1.187173223 | 0.9 | 0.3 | 4.49E-137 | 5 | S100A2 |  |
| 5.95E-53 | 0.878454074 | 0.5 | 0.23 | 1.99E-48 | 5 | PTTG1 |  |
| 0 | 1.79310678 | 0.98 | 0.43 | 0 | 6 | HMGB2 | KC-5 |
| 0 | 1.449112892 | 0.79 | 0.02 | 0 | 6 | TK1 |  |
| 0 | 1.416990471 | 1 | 0.73 | 0 | 6 | H2AFZ |  |
| 0 | 1.547909537 | 1 | 0.81 | 1.26E-307 | 6 | TUBA1B |  |
| 5.64E-277 | 1.444244077 | 0.65 | 0.01 | 1.89E-272 | 6 | UBE2C |  |
| 3.75E-211 | 1.502358865 | 0.97 | 0.32 | 1.26E-206 | 6 | STMN1 |  |
| 1.29E-158 | 1.373925011 | 0.99 | 0.69 | 4.33E-154 | 6 | MT2A |  |
| 2.51E-145 | 1.260387525 | 0.58 | 0.06 | 8.43E-141 | 6 | CENPF |  |
| 4.15E-142 | 1.49665125 | 0.73 | 0.22 | 1.39E-137 | 6 | PTTG1 |  |
| 1.23E-130 | 2.030627549 | 0.9 | 0.66 | 4.13E-126 | 6 | HIST1H4C |  |
| 0 | 3.758006174 | 1 | 0.06 | 0 | 7 | HLA-DQB2 | LC |
| 0 | 3.528166857 | 0.99 | 0.02 | 0 | 7 | FCGBP |  |
| 0 | 3.027902636 | 1 | 0.3 | 0 | 7 | CD74 |  |
| 0 | 2.991879023 | 1 | 0.06 | 0 | 7 | FCER1A |  |
| 0 | 2.890214866 | 1 | 0.02 | 0 | 7 | CD207 |  |
| 0 | 2.717196643 | 1 | 0.22 | 0 | 7 | HLA-DQB1 |  |
| 0 | 2.701499713 | 0.99 | 0.04 | 0 | 7 | CD1A |  |
| 0 | 2.674596607 | 1 | 0.23 | 0 | 7 | HLA-DPB1 |  |
| 0 | 2.642331942 | 1 | 0.27 | 0 | 7 | HLA-DRB1 |  |
| 6.02E-286 | 2.833279706 | 0.86 | 0.12 | 2.02E-281 | 7 | HLA-DRB5 |  |
| 0 | 3.986940773 | 0.84 | 0.01 | 0 | 8 | LYZ | DC |
| 0 | 3.045763865 | 1 | 0.16 | 0 | 8 | HLA-DRA |  |
| 0 | 3.035661779 | 0.98 | 0.61 | 0 | 8 | CST3 |  |
| 0 | 2.850267832 | 0.97 | 0.26 | 0 | 8 | HLA-DPA1 |  |
| 1.35E-305 | 2.700728068 | 0.97 | 0.23 | 4.51E-301 | 8 | HLA-DPB1 |  |
| 3.96E-284 | 2.744047631 | 0.93 | 0.12 | 1.33E-279 | 8 | HLA-DQA1 |  |
| 5.26E-271 | 2.672997458 | 0.97 | 0.27 | 1.76E-266 | 8 | HLA-DRB1 |  |
| 1.65E-237 | 3.327248031 | 0.63 | 0.02 | 5.54E-233 | 8 | IL1B |  |
| 3.72E-147 | 2.576183684 | 0.58 | 0.06 | 1.25E-142 | 8 | C15orf48 |  |
| 2.15E-95 | 2.673054533 | 0.26 | 0 | 7.22E-91 | 8 | MMP12 |  |
| 0 | 3.117186478 | 0.93 | 0.05 | 0 | 10 | NKG7 | TC-2 |
| 0 | 2.752238911 | 0.91 | 0.03 | 8.44E-307 | 10 | GZMA |  |
| 2.37E-304 | 2.468329959 | 0.83 | 0.01 | 7.95E-300 | 10 | GZMH |  |
| 4.60E-303 | 3.165726656 | 0.95 | 0.07 | 1.54E-298 | 10 | CCL5 |  |
| 4.91E-263 | 2.812877708 | 0.74 | 0.01 | 1.65E-258 | 10 | GZMK |  |
| 5.94E-205 | 2.209840249 | 0.71 | 0.02 | 1.99E-200 | 10 | GZMB |  |
| 1.29E-203 | 2.524628456 | 0.82 | 0.04 | 4.33E-199 | 10 | CCL4 |  |
| 5.93E-167 | 1.8190559 | 0.9 | 0.17 | 1.99E-162 | 10 | CST7 |  |
| 9.22E-164 | 1.905892571 | 0.69 | 0.04 | 3.09E-159 | 10 | CD8A |  |
| 7.87E-32 | 2.130202021 | 0.27 | 0.04 | 2.64E-27 | 10 | GNLY |  |
| 4.32E-137 | 1.573232917 | 0.98 | 0.22 | 1.45E-132 | 11 | IL32 | TC-3 |
| 9.97E-130 | 1.722500003 | 0.88 | 0.08 | 3.34E-125 | 11 | CCL5 |  |
| 1.46E-127 | 1.579663261 | 0.86 | 0.12 | 4.91E-123 | 11 | LINC01871 |  |
| 1.33E-126 | 1.390019702 | 0.98 | 0.26 | 4.47E-122 | 11 | HCST |  |
| 1.28E-123 | 1.392941183 | 0.96 | 0.17 | 4.30E-119 | 11 | CD3D |  |
| 4.95E-123 | 1.464262545 | 0.66 | 0.04 | 1.66E-118 | 11 | TRGC2 |  |
| 1.58E-122 | 1.474623531 | 0.93 | 0.13 | 5.31E-118 | 11 | CTSW |  |
| 4.91E-114 | 1.40178904 | 0.97 | 0.21 | 1.65E-109 | 11 | CXCR4 |  |
| 3.47E-109 | 1.463680359 | 0.93 | 0.56 | 1.16E-104 | 11 | ZFP36L2 |  |
| 8.18E-102 | 1.430880967 | 0.68 | 0.04 | 2.74E-97 | 11 | CD8A |  |
| 2.96E-157 | 1.574499889 | 0.65 | 0.01 | 9.92E-153 | 12 | CTLA4 | TREG |
| 2.01E-141 | 1.570147418 | 0.75 | 0.04 | 6.74E-137 | 12 | TIGIT |  |
| 9.41E-134 | 1.733623621 | 0.97 | 0.29 | 3.15E-129 | 12 | TRAC |  |
| 6.91E-132 | 1.574335385 | 0.77 | 0.06 | 2.32E-127 | 12 | CD27 |  |
| 6.32E-131 | 1.735268676 | 1 | 0.22 | 2.12E-126 | 12 | IL32 |  |
| 1.85E-109 | 1.842219585 | 0.89 | 0.17 | 6.19E-105 | 12 | TRBC2 |  |
| 6.04E-96 | 1.526746011 | 0.84 | 0.19 | 2.03E-91 | 12 | DUSP4 |  |
| 3.39E-81 | 1.457867198 | 0.91 | 0.25 | 1.14E-76 | 12 | PTPRC |  |
| 6.51E-67 | 1.912883202 | 0.65 | 0.11 | 2.18E-62 | 12 | TNFRSF4 |  |
| 6.98E-62 | 1.587132583 | 0.65 | 0.12 | 2.34E-57 | 12 | TRBC1 |  |
| 1.26E-290 | 2.79780168 | 1 | 0.13 | 4.24E-286 | 13 | CTSW | TC-4 |
| 1.30E-275 | 2.423591317 | 0.93 | 0.03 | 4.36E-271 | 13 | SPINK2 |  |
| 3.03E-252 | 3.034010272 | 0.92 | 0.02 | 1.02E-247 | 13 | XCL1 |  |
| 6.92E-239 | 2.817289125 | 0.99 | 0.22 | 2.32E-234 | 13 | AREG |  |
| 9.29E-216 | 2.48434177 | 0.84 | 0.01 | 3.12E-211 | 13 | XCL2 |  |
| 1.78E-208 | 1.939326548 | 0.99 | 0.37 | 5.99E-204 | 13 | TNFRSF18 |  |
| 1.67E-176 | 1.775760592 | 0.99 | 0.39 | 5.59E-172 | 13 | FXYD5 |  |
| 2.82E-162 | 2.057470694 | 0.93 | 0.09 | 9.47E-158 | 13 | FCER1G |  |
| 3.30E-117 | 2.33994206 | 0.71 | 0.03 | 1.11E-112 | 13 | GNLY |  |
| 5.75E-45 | 2.867840442 | 0.22 | 0 | 1.93E-40 | 13 | CCL1 |  |
| 2.54E-66 | 1.357868196 | 0.44 | 0.02 | 8.53E-62 | 14 | CCR7 | TC-5 |
| 5.27E-61 | 1.453254477 | 0.49 | 0.06 | 1.77E-56 | 14 | GIMAP7 |  |
| 6.09E-57 | 1.599048182 | 0.75 | 0.22 | 2.04E-52 | 14 | LTB |  |
| 7.53E-46 | 1.424833315 | 0.64 | 0.17 | 2.53E-41 | 14 | IL7R |  |
| 1.31E-44 | 1.150370329 | 0.63 | 0.22 | 4.39E-40 | 14 | LIMD2 |  |
| 9.60E-40 | 1.124042302 | 0.65 | 0.39 | 3.22E-35 | 14 | LDHB |  |
| 8.38E-38 | 1.142193558 | 0.74 | 0.26 | 2.81E-33 | 14 | PTPRC |  |
| 4.70E-35 | 1.041655123 | 0.84 | 0.31 | 1.58E-30 | 14 | CD52 |  |
| 5.96E-32 | 1.092187015 | 0.67 | 0.18 | 2.00E-27 | 14 | TRBC2 |  |
| 2.63E-18 | 1.220212893 | 0.35 | 0.17 | 8.80E-14 | 14 | KLF2 |  |
| 2.79E-50 | 4.73978787 | 1 | 0.21 | 9.34E-46 | 15 | KRT17 | KC-6 |
| 2.65E-28 | 2.205894436 | 1 | 0.54 | 8.89E-24 | 15 | CXCL14 |  |
| 4.10E-28 | 1.574159294 | 0.54 | 0.01 | 1.37E-23 | 15 | TNC |  |
| 8.80E-23 | 1.326420618 | 0.58 | 0.02 | 2.95E-18 | 15 | CPM |  |
| 2.05E-22 | 1.61148786 | 0.63 | 0.02 | 6.89E-18 | 15 | IL20 |  |
| 4.85E-20 | 1.909796736 | 0.96 | 0.34 | 1.63E-15 | 15 | DST |  |
| 3.12E-19 | 2.457280964 | 0.92 | 0.21 | 1.05E-14 | 15 | CYR61 |  |
| 7.56E-19 | 1.517008625 | 0.67 | 0.16 | 2.54E-14 | 15 | DCN |  |
| 5.45E-17 | 1.497337969 | 0.42 | 0.01 | 1.83E-12 | 15 | CTGF |  |
| 7.93E-09 | 1.305259967 | 0.58 | 0.2 | 0.000265945 | 15 | SPINK5 |  |

**Supplementary Table 2: Top 10 differentially expressed genes** according to highest average log fold change (avg logFC) ordered by smallest adjusted p-value using logistic regression with Bonferroni correction for each cluster, as compared to the rest of the dataset in all sequenced skin cells (comparison spontaneously healed AD vs. healthy control skin). pct.1/2: The percentage of cells in which the feature is detected in the first/second group.

| **Protein name** | **logFCH** | **AveExpr** | **t** | **P.Value** | **adj.P.Val** | **B** |
| --- | --- | --- | --- | --- | --- | --- |
| CD244.1 | -0.6851868 | 3.82984733 | -2.9335751 | 0.00986361 | 0.80610042 | -4.3885839 |
| CCL4.1 | -1.8131718 | 4.19437 | -2.8742381 | 0.0111489 | 0.80610042 | -4.3952465 |
| CCL4 | -1.8259904 | 4.110052 | -2.859777 | 0.01148605 | 0.80610042 | -4.3968775 |
| CCL3.1 | -1.3744054 | 5.966756 | -2.5548913 | 0.02138047 | 0.80610042 | -4.4317989 |
| PDCD1 | -0.6209911 | 2.04571667 | -2.5481347 | 0.02167319 | 0.80610042 | -4.4325819 |
| FCRL6 | -0.6951382 | 1.266898 | -2.5433387 | 0.02188329 | 0.80610042 | -4.4331378 |
| CCL3 | -1.3428193 | 5.59784 | -2.4733842 | 0.02517696 | 0.80610042 | -4.4412604 |
| ADAM15 | -0.5090418 | 7.322374 | -2.391441 | 0.02963276 | 0.80610042 | -4.4507991 |
| PRTFDC1 | -0.6226707 | 2.077094 | -2.2831618 | 0.03666632 | 0.80610042 | -4.4634166 |
| MCP.1.1 | -1.0722271 | 11.3488527 | -2.1793175 | 0.04485038 | 0.80610042 | -4.4754967 |
| CD244 | -0.5887704 | 2.91399733 | -2.1085124 | 0.05136674 | 0.80610042 | -4.4837018 |
| EFNA4 | -0.5344986 | 3.80227467 | -2.1033261 | 0.0518767 | 0.80610042 | -4.4843013 |
| EREG | -0.7844611 | 4.43716333 | -2.0736406 | 0.05488601 | 0.80610042 | -4.4877286 |
| MCP.1 | -1.0348614 | 10.5260993 | -2.062461 | 0.05606011 | 0.80610042 | -4.4890172 |
| IL7.1 | -0.4649571 | 2.60088733 | -1.9908489 | 0.06413993 | 0.80610042 | -4.4972396 |
| CDSN | 0.42737071 | 8.67206533 | 1.97272514 | 0.06634561 | 0.80610042 | -4.4993107 |
| TRIM21 | -0.4441289 | 2.351604 | -1.9691619 | 0.06678725 | 0.80610042 | -4.4997174 |
| RPS6KB1 | -0.6540539 | 4.790132 | -1.9499257 | 0.06921753 | 0.80610042 | -4.5019098 |
| CCL11.1 | -0.6078361 | 3.975568 | -1.9168375 | 0.0735847 | 0.80610042 | -4.5056683 |
| MUC.16 | -1.1967164 | 3.552174 | -1.9084937 | 0.07472429 | 0.80610042 | -4.5066133 |
| TGF.alpha | -0.6556454 | 5.877876 | -1.8899385 | 0.07731539 | 0.80610042 | -4.5087109 |
| Flt3L | -0.731445 | 9.62566933 | -1.8472418 | 0.08358462 | 0.80610042 | -4.5135147 |
| CXCL1.1 | -0.8460157 | 7.049578 | -1.809113 | 0.08956056 | 0.80610042 | -4.5177755 |
| LAP.TGF.beta.1.1 | -0.6685929 | 4.826524 | -1.8013867 | 0.09081654 | 0.80610042 | -4.5186353 |
| CD28 | -0.60864 | 1.172156 | -1.797214 | 0.09150128 | 0.80610042 | -4.5190992 |
| TANK | -0.5224307 | 4.627088 | -1.7966838 | 0.09158861 | 0.80610042 | -4.5191581 |
| CST5 | -0.43041 | 4.336758 | -1.7743647 | 0.09533211 | 0.80610042 | -4.5216324 |
| PTS | -0.5951825 | 5.586348 | -1.7601734 | 0.09778169 | 0.80610042 | -4.5232 |
| SLAMF1 | -0.3727557 | 1.398912 | -1.7583816 | 0.09809486 | 0.80610042 | -4.5233976 |
| SMOC1 | -0.6001675 | 3.76668 | -1.7560709 | 0.09850003 | 0.80610042 | -4.5236523 |
| IL.18R1 | -0.5497129 | 5.37206667 | -1.7499115 | 0.09958721 | 0.80610042 | -4.5243306 |
| GDF.8 | -0.4668089 | 2.21429933 | -1.7495594 | 0.09964967 | 0.80610042 | -4.5243694 |
| LAP.TGF.beta.1 | -0.7473 | 5.53878067 | -1.7468635 | 0.10012909 | 0.80610042 | -4.524666 |
| N.CDase | -0.7489986 | 0.78788733 | -1.7398134 | 0.10139231 | 0.80610042 | -4.5254408 |
| EPHB6 | -0.6414446 | 6.44665133 | -1.7326644 | 0.10268746 | 0.80610042 | -4.5262253 |
| MMP.10 | -0.4756471 | 5.07359333 | -1.7270937 | 0.10370666 | 0.80610042 | -4.5268358 |
| KLRD1.1 | -0.4377714 | 2.63913067 | -1.7253562 | 0.10402636 | 0.80610042 | -4.527026 |
| FCRL2 | -0.3506236 | 1.75438133 | -1.717789 | 0.1054287 | 0.80610042 | -4.5278537 |
| TDGF1 | -0.68916 | 0.83736933 | -1.7132445 | 0.10627876 | 0.80610042 | -4.5283501 |
| BMP.4 | -0.4523586 | 2.81864667 | -1.7060865 | 0.10762978 | 0.80610042 | -4.5291309 |
| BCAN | -0.7884186 | 5.64780667 | -1.6945458 | 0.1098394 | 0.80610042 | -4.530387 |
| CXCL13 | 0.99892857 | 5.51345333 | 1.69383783 | 0.10997622 | 0.80610042 | -4.530464 |
| FASLG | -0.4766339 | 3.44532333 | -1.6609825 | 0.11649022 | 0.80610042 | -4.5340202 |
| NOS3 | 0.45359036 | 0.94918067 | 1.65732057 | 0.11723646 | 0.80610042 | -4.5344147 |
| CLEC10A | -0.5168329 | 2.79907933 | -1.6522017 | 0.11828649 | 0.80610042 | -4.5349656 |
| TRAIL.1 | -0.6664789 | 6.236228 | -1.6456121 | 0.1196501 | 0.80610042 | -4.5356737 |
| FLRT2 | -0.5585543 | 4.86073667 | -1.62437 | 0.12413812 | 0.80610042 | -4.5379479 |
| CASP.8.1 | -0.4256729 | 4.90855533 | -1.611702 | 0.12688261 | 0.80610042 | -4.5392979 |
| VEGFA.1 | -0.6398464 | 8.119742 | -1.5890223 | 0.13192554 | 0.80610042 | -4.5417027 |
| CDCP1 | -0.5976696 | 6.790348 | -1.565822 | 0.13725946 | 0.80610042 | -4.5441461 |
| uPA | -0.5029921 | 8.403514 | -1.5448592 | 0.1422348 | 0.80610042 | -4.5463389 |
| CD200 | -0.3928989 | 3.91090667 | -1.5429205 | 0.14270251 | 0.80610042 | -4.546541 |
| IL10.2 | -1.0301729 | 5.567268 | -1.5417395 | 0.14298807 | 0.80610042 | -4.5466641 |
| MAD1L1 | -0.4546275 | 4.82653467 | -1.5154377 | 0.14947315 | 0.80610042 | -4.5493919 |
| CXCL1 | -0.7204714 | 5.19560733 | -1.5136564 | 0.14992114 | 0.80610042 | -4.5495758 |
| CSF.1.1 | -0.5480361 | 8.16635733 | -1.5117527 | 0.15040114 | 0.80610042 | -4.5497722 |
| IFNLR1 | -0.392695 | 1.92911333 | -1.5088224 | 0.15114249 | 0.80610042 | -4.5500743 |
| EN.RAGE | 0.432385 | 2.36032467 | 1.50583682 | 0.15190098 | 0.80610042 | -4.5503817 |
| TNFRSF21.1 | -0.3828396 | 6.83865733 | -1.4991834 | 0.15360271 | 0.80610042 | -4.5510657 |
| CRADD | -0.4303543 | 7.313446 | -1.4945443 | 0.15479865 | 0.80610042 | -4.5515418 |
| IL.22.RA1 | -0.4495121 | 2.56774067 | -1.4942468 | 0.1548756 | 0.80610042 | -4.5515723 |
| PD.L1.1 | -0.4138857 | 4.11626267 | -1.4888441 | 0.15627871 | 0.80610042 | -4.5521256 |
| Siglec.9 | -0.4137357 | 3.09519267 | -1.4814279 | 0.15822196 | 0.80610042 | -4.5528834 |
| IL6.2 | -1.4401079 | 4.45151933 | -1.4810447 | 0.1587354 | 0.80610042 | -4.5530278 |
| AARSD1 | -0.2886618 | 6.605638 | -1.4765418 | 0.15951318 | 0.80610042 | -4.5533816 |
| UNC5C | -0.6184504 | 1.95645533 | -1.4729319 | 0.16047278 | 0.80610042 | -4.5537492 |
| IL7 | -0.48938 | 3.40738267 | -1.470317 | 0.16117083 | 0.80610042 | -4.5540151 |
| NTRK2 | -0.3557582 | 4.32928267 | -1.4536044 | 0.16569208 | 0.80610042 | -4.5557086 |
| HCLS1 | 0.79564036 | 4.53235 | 1.4513414 | 0.16631226 | 0.80610042 | -4.5559371 |
| LEPR | -0.3873736 | 1.593664 | -1.4499652 | 0.16669033 | 0.80610042 | -4.556076 |
| CXADR | -0.4712843 | 2.70476667 | -1.4456119 | 0.16789101 | 0.80610042 | -4.5565148 |
| TNFRSF21 | -0.4444286 | 6.33833267 | -1.4428208 | 0.16866456 | 0.80610042 | -4.5567958 |
| CTSC | -0.4344868 | 3.43494333 | -1.4402468 | 0.16938051 | 0.80610042 | -4.5570546 |
| IL10.1 | -0.9956982 | 4.491188 | -1.4403885 | 0.16947061 | 0.80610042 | -4.557069 |
| HGF.1 | -0.6112657 | 8.81748667 | -1.4310908 | 0.1719476 | 0.80610042 | -4.5579732 |
| NEFL | -0.9023107 | 2.02421067 | -1.4218381 | 0.17498135 | 0.80610042 | -4.5589823 |
| ATP6V1F | -0.3483179 | 3.40652267 | -1.4177961 | 0.1757318 | 0.80610042 | -4.5593013 |
| CD33 | -0.8059675 | 2.64840267 | -1.414965 | 0.1765464 | 0.80610042 | -4.5595832 |
| PLA2G10 | -0.3938571 | 1.90714533 | -1.4139064 | 0.17685178 | 0.80610042 | -4.5596886 |
| RSPO1 | -0.5708507 | 6.22276867 | -1.4002276 | 0.18083675 | 0.80610042 | -4.5610455 |
| SMOC2 | -0.6165382 | 8.235928 | -1.39554 | 0.18221908 | 0.80610042 | -4.5615088 |
| GBP2 | -0.2876143 | 1.67958067 | -1.3933969 | 0.18285392 | 0.80610042 | -4.5617203 |
| IL15 | -0.4555368 | 3.81005733 | -1.3889207 | 0.18418568 | 0.80610042 | -4.5621615 |
| IL6.1 | -1.3017836 | 3.34373267 | -1.3895091 | 0.18441363 | 0.80610042 | -4.5621771 |
| ADGRB3 | -0.3830493 | 3.03718 | -1.3804976 | 0.18671307 | 0.80610042 | -4.5629894 |
| IL.10RB | -0.3590668 | 3.71566467 | -1.371531 | 0.18943438 | 0.80610042 | -4.5638676 |
| TWEAK.1 | -0.54236 | 9.13287733 | -1.3578319 | 0.19365377 | 0.80610042 | -4.5652026 |
| VEGFA | -0.5287579 | 5.94944533 | -1.3497828 | 0.19616803 | 0.80610042 | -4.5659833 |
| IL.20RA | -0.3044454 | 2.15062933 | -1.3405957 | 0.19906978 | 0.80610042 | -4.566871 |
| IL8.1 | -1.1844143 | 7.95293133 | -1.3363066 | 0.20083223 | 0.80610042 | -4.5673419 |
| PD.L1 | -0.344235 | 2.51579867 | -1.3262384 | 0.20367331 | 0.80610042 | -4.5682508 |
| CASP.8 | -0.3846479 | 6.91289933 | -1.3220232 | 0.20504093 | 0.80610042 | -4.5686542 |
| MIC.A.B | 0.57466107 | 1.585832 | 1.32176519 | 0.20512487 | 0.80610042 | -4.5686788 |
| TRAIL | -0.5647764 | 6.105954 | -1.3153844 | 0.20720969 | 0.80610042 | -4.5692879 |
| OSM | -0.7522971 | 4.233262 | -1.3078107 | 0.20970612 | 0.80610042 | -4.5700084 |
| TPSAB1 | -0.4919443 | 5.57535867 | -1.3052449 | 0.21055726 | 0.80610042 | -4.5702519 |
| CD27 | -0.3850232 | 5.80362133 | -1.3032062 | 0.2112355 | 0.80610042 | -4.5704452 |
| ADGRG1 | -0.3602925 | 1.63717267 | -1.2978377 | 0.21302982 | 0.80610042 | -4.5709532 |
| CCL25 | -0.4271421 | 3.60056667 | -1.2950937 | 0.21395159 | 0.80610042 | -4.5712124 |
| IL6 | -1.1104225 | 3.38274733 | -1.2823512 | 0.21866027 | 0.80657067 | -4.5724551 |
| CSF.1 | -0.4921229 | 7.43004867 | -1.2789616 | 0.21943466 | 0.80657067 | -4.572729 |
| BST2 | -0.2658464 | 1.95245133 | -1.2754363 | 0.22064748 | 0.80657067 | -4.5730588 |
| ICOSLG | -0.3270936 | 3.74165133 | -1.2502748 | 0.22945747 | 0.80657067 | -4.5753958 |
| LAYN | -0.3732054 | 5.817376 | -1.246422 | 0.23083044 | 0.80657067 | -4.575751 |
| SFRP1 | -0.6973136 | 10.6416153 | -1.2395712 | 0.23328752 | 0.80657067 | -4.5763807 |
| HGF | -0.5601961 | 8.18112467 | -1.2372258 | 0.23413339 | 0.80657067 | -4.5765958 |
| NAA10 | -0.3560261 | 7.49264067 | -1.236552 | 0.23437684 | 0.80657067 | -4.5766575 |
| HNMT | -0.5303371 | 11.9747627 | -1.2248926 | 0.2386206 | 0.80657067 | -4.5777223 |
| DDR1 | -0.3153761 | 7.80842267 | -1.2224356 | 0.23952244 | 0.80657067 | -4.5779459 |
| IMPA1 | -0.2528557 | 2.88716067 | -1.2098336 | 0.24418951 | 0.80657067 | -4.5790877 |
| LY75 | -0.3143593 | 1.71931667 | -1.2022598 | 0.24702791 | 0.80657067 | -4.57977 |
| KLRD1 | -0.3121139 | 3.21115 | -1.199792 | 0.24795824 | 0.80657067 | -4.5799918 |
| IL8 | -1.0605929 | 7.517286 | -1.1988751 | 0.24867406 | 0.80657067 | -4.5800997 |
| PGF | -0.4281146 | 6.81734 | -1.1949123 | 0.2498057 | 0.80657067 | -4.5804293 |
| EDAR | -0.2456661 | 1.516034 | -1.1915701 | 0.25107714 | 0.80657067 | -4.5807282 |
| GPNMB | -0.2360246 | 6.176242 | -1.191268 | 0.25119231 | 0.80657067 | -4.5807552 |
| THY.1 | -0.4124704 | 9.82812133 | -1.1865417 | 0.25299938 | 0.80657067 | -4.5811769 |
| Alpha.2.MRAP | -0.6510189 | 6.58946467 | -1.1692181 | 0.25970789 | 0.81627189 | -4.5827128 |
| Nr.CAM | -0.2960896 | 7.043074 | -1.1621226 | 0.26249432 | 0.81627189 | -4.5833373 |
| PLXNB3 | -0.1972579 | 2.057812 | -1.1618354 | 0.26260758 | 0.81627189 | -4.5833626 |
| CX3CL1.1 | -0.3947861 | 3.45324733 | -1.1367383 | 0.27264831 | 0.81849457 | -4.5855499 |
| DNER | -0.3618239 | 6.23319067 | -1.1283757 | 0.27605726 | 0.81849457 | -4.5862712 |
| CD5.1 | -0.3484675 | 2.632816 | -1.121821 | 0.27875146 | 0.81849457 | -4.5868339 |
| N2DL.2 | -0.4532675 | 4.44294 | -1.1199915 | 0.27950692 | 0.81849457 | -4.5869906 |
| IFNL1 | -0.2583039 | 1.129902 | -1.1150918 | 0.28153776 | 0.81849457 | -4.5874092 |
| GZMA | -0.56705 | 4.07848933 | -1.1103738 | 0.28350362 | 0.81849457 | -4.5878111 |
| CX3CL1 | -0.4461143 | 4.38999667 | -1.1102095 | 0.28357227 | 0.81849457 | -4.587825 |
| CD200R1 | -0.2877243 | 2.64480667 | -1.1018761 | 0.2870701 | 0.81849457 | -4.5885318 |
| CCL11 | -0.3993354 | 4.15165733 | -1.1014069 | 0.28726798 | 0.81849457 | -4.5885714 |
| CRTAM | -0.382635 | 2.515772 | -1.0971383 | 0.28907294 | 0.81849457 | -4.5889318 |
| CETN2 | -0.3532504 | 2.93629267 | -1.0930787 | 0.29079728 | 0.81849457 | -4.5892736 |
| JAM.B | -0.3344007 | 6.909452 | -1.0880768 | 0.29293228 | 0.81849457 | -4.5896935 |
| MMP12 | 1.03865679 | 7.85513533 | 1.08318113 | 0.29537309 | 0.81849457 | -4.5901102 |
| CRTAM.1 | -0.3179193 | 2.41204933 | -1.0806661 | 0.29611663 | 0.81849457 | -4.590313 |
| LIF | -0.4388571 | 2.10812333 | -1.0706477 | 0.30046182 | 0.81849457 | -4.5911455 |
| sFRP.3 | -0.4613361 | 5.17201667 | -1.0704734 | 0.30053782 | 0.81849457 | -4.5911599 |
| FGF.19 | -0.4305489 | 5.807234 | -1.0658549 | 0.30255698 | 0.81849457 | -4.5915417 |
| CPA2 | -0.4495589 | 5.90223067 | -1.0639886 | 0.3033757 | 0.81849457 | -4.5916957 |
| ADAM.22 | -0.3399218 | 3.01796067 | -1.0602619 | 0.3050154 | 0.81849457 | -4.5920025 |
| CD40.1 | -0.3560764 | 9.739654 | -1.0509728 | 0.30913053 | 0.82051263 | -4.5927637 |
| CD8A.1 | -0.3693654 | 5.953074 | -1.0452574 | 0.3116824 | 0.82051263 | -4.5932295 |
| TWEAK | -0.4647107 | 7.93084067 | -1.0437305 | 0.31236674 | 0.82051263 | -4.5933537 |
| GALNT3 | -0.2253532 | 2.08188467 | -1.0339815 | 0.31676162 | 0.82623836 | -4.594143 |
| PTPN1 | -0.3354307 | 6.52158533 | -1.0148189 | 0.32552956 | 0.83627698 | -4.5956783 |
| PRTG | -0.2423171 | 4.09204067 | -1.0076865 | 0.32883689 | 0.83627698 | -4.5962441 |
| WWP2 | -0.2498546 | 6.96952667 | -1.00102 | 0.3319497 | 0.83627698 | -4.5967702 |
| HMOX2 | -0.4210089 | 4.374058 | -0.9996097 | 0.33261087 | 0.83627698 | -4.5968811 |
| CDH17 | -0.3102614 | 1.08910933 | -0.9872325 | 0.3384537 | 0.83627698 | -4.5978496 |
| MCP.2.1 | -0.4219654 | 7.88289 | -0.9859663 | 0.33905548 | 0.83627698 | -4.5979482 |
| MCP.2 | -0.430845 | 6.07298733 | -0.984171 | 0.33991005 | 0.83627698 | -4.5980878 |
| IL10 | -0.6715839 | 5.514914 | -0.9802855 | 0.34176464 | 0.83627698 | -4.5983891 |
| OPG | -0.4056607 | 7.30271067 | -0.9782516 | 0.34273832 | 0.83627698 | -4.5985465 |
| G.CSF | -0.3590882 | 1.99874267 | -0.977642 | 0.3430305 | 0.83627698 | -4.5985937 |
| CDH3 | -0.3868193 | 7.12423 | -0.9719882 | 0.3457489 | 0.83743077 | -4.5990296 |
| PDGF.subunit.B | -0.2528261 | 2.188612 | -0.9647623 | 0.34924501 | 0.84044121 | -4.5995839 |
| CD6 | -0.3345454 | 2.39212333 | -0.9592121 | 0.35194702 | 0.84151435 | -4.6000075 |
| NFATC3 | -0.2031879 | 1.378208 | -0.9436207 | 0.35961503 | 0.84976544 | -4.601187 |
| RGMB | -0.2845364 | 4.542826 | -0.9429363 | 0.35995426 | 0.84976544 | -4.6012384 |
| GZMA.1 | -0.4278229 | 3.469452 | -0.936327 | 0.36324143 | 0.84992903 | -4.6017335 |
| SCARF2 | -0.2756339 | 4.19470933 | -0.9267036 | 0.36806443 | 0.84992903 | -4.6024493 |
| SKR3 | -0.2485918 | 5.62163867 | -0.9216984 | 0.3705901 | 0.84992903 | -4.6028193 |
| CLM.6 | -0.2707514 | 4.09243 | -0.92038 | 0.37125738 | 0.84992903 | -4.6029165 |
| ROBO2 | -0.2767786 | 3.34497333 | -0.9200654 | 0.37141671 | 0.84992903 | -4.6029397 |
| TNFSF14.1 | -0.3544475 | 2.51072733 | -0.9107194 | 0.37617156 | 0.85252689 | -4.6036252 |
| IL15.1 | -0.20537 | 1.99603267 | -0.9021681 | 0.38055809 | 0.85252689 | -4.6042476 |
| SNCG | -0.6612589 | 4.22956667 | -0.8942407 | 0.38493656 | 0.85252689 | -4.6048107 |
| RBKS | -0.2026182 | 7.41382267 | -0.8927662 | 0.38542065 | 0.85252689 | -4.6049263 |
| SPOCK1 | -0.2465807 | 1.93733667 | -0.8926143 | 0.38549955 | 0.85252689 | -4.6049372 |
| KIF1BP | -0.1773257 | 6.43904067 | -0.8889984 | 0.38738094 | 0.85252689 | -4.6051966 |
| GFR.alpha.1 | -0.3040989 | 8.46002067 | -0.8867553 | 0.38855113 | 0.85252689 | -4.6053572 |
| X4E.BP1 | -0.2135875 | 10.7715627 | -0.8777292 | 0.39328375 | 0.85732557 | -4.6059997 |
| CD70 | 0.26166214 | 2.43169333 | 0.87383928 | 0.39533511 | 0.85732557 | -4.6062749 |
| DEFB4A | -0.8520786 | 0.08940067 | -0.8510072 | 0.40778495 | 0.87878473 | -4.6078588 |
| CLM.1 | -0.3145586 | 4.38284533 | -0.8425491 | 0.41209341 | 0.87878473 | -4.6084522 |
| PPP3R1 | -0.2175325 | 4.78560267 | -0.8421715 | 0.41229846 | 0.87878473 | -4.6084781 |
| CD5 | -0.3008839 | 2.975118 | -0.8373027 | 0.41494798 | 0.87940681 | -4.6088108 |
| STAMBP | -0.3230411 | 8.47873933 | -0.8253253 | 0.42151273 | 0.88219383 | -4.6096225 |
| CLEC7A | -0.3878886 | 3.18832 | -0.82514 | 0.42161483 | 0.88219383 | -4.6096349 |
| CD40 | -0.2931821 | 7.59228333 | -0.8213084 | 0.42372929 | 0.88219383 | -4.6098925 |
| MSR1 | -0.3773029 | 4.52127467 | -0.8177058 | 0.42572357 | 0.88219383 | -4.6101337 |
| PTN | -0.4610618 | 6.24665933 | -0.8101778 | 0.42991025 | 0.88594765 | -4.6106348 |
| PSME1 | -0.2103232 | 6.41961867 | -0.8054643 | 0.43254492 | 0.88647941 | -4.6109466 |
| CD83.1 | -0.2207271 | 1.63380067 | -0.7917764 | 0.44025402 | 0.88993481 | -4.6118432 |
| NF2 | -0.3718421 | 2.51296267 | -0.7914687 | 0.44042828 | 0.88993481 | -4.6118632 |
| MATN3 | -0.3799654 | 6.25952667 | -0.7895771 | 0.44150067 | 0.88993481 | -4.6119861 |
| BIRC2 | -0.2937796 | 2.80174467 | -0.7846108 | 0.44432396 | 0.88993481 | -4.6123074 |
| SH2D1A | 0.32601464 | 1.72601067 | 0.78139117 | 0.44616035 | 0.88993481 | -4.6125147 |
| CXCL6 | -0.2969575 | 4.07502 | -0.775923 | 0.44929002 | 0.89141052 | -4.6128653 |
| LILRB4 | -0.2036082 | 1.712382 | -0.770368 | 0.45248343 | 0.8929964 | -4.6132192 |
| GDNFR.alpha.3 | -0.2172121 | 4.39912267 | -0.7640756 | 0.45611765 | 0.89543096 | -4.6136175 |
| ARG1 | -0.3834261 | 5.540088 | -0.7574233 | 0.45997925 | 0.8979086 | -4.6140355 |
| DFFA | -0.271075 | 9.83476133 | -0.7508397 | 0.46382067 | 0.8979086 | -4.614446 |
| MCP.3 | -0.2968261 | 1.03565067 | -0.7478924 | 0.4655467 | 0.8979086 | -4.6146288 |
| RGMA | -0.2166389 | 8.53068133 | -0.7371228 | 0.47188701 | 0.8979086 | -4.6152914 |
| CD63 | -0.3231496 | 2.07229733 | -0.7324937 | 0.47462822 | 0.8979086 | -4.6155736 |
| TNFRSF9.1 | -0.2473218 | 5.30925133 | -0.731692 | 0.47510397 | 0.8979086 | -4.6156224 |
| IL.17C | 0.48316607 | 2.18890533 | 0.72326521 | 0.48034369 | 0.8979086 | -4.6161179 |
| CD8A | -0.2885882 | 5.27410933 | -0.7186629 | 0.48287544 | 0.8979086 | -4.6164076 |
| CTSS | -0.1871007 | 5.48802333 | -0.7165778 | 0.48412613 | 0.8979086 | -4.6165321 |
| CDH15 | -0.2709482 | 2.76480867 | -0.7077573 | 0.48943817 | 0.8979086 | -4.6170554 |
| BACH1 | -0.2828071 | 5.27286133 | -0.7052625 | 0.49094692 | 0.8979086 | -4.6172023 |
| NTRK3 | -0.18027 | 4.084812 | -0.7022255 | 0.49278719 | 0.8979086 | -4.6173806 |
| ING1 | -0.2887504 | 4.49426067 | -0.7015467 | 0.49319906 | 0.8979086 | -4.6174203 |
| PMVK | -0.1373321 | 7.77638333 | -0.6996888 | 0.49432744 | 0.8979086 | -4.617529 |
| NRP2 | -0.1997957 | 6.77884867 | -0.6975652 | 0.49561899 | 0.8979086 | -4.6176528 |
| SIGLEC1 | -0.215395 | 4.66747 | -0.6971103 | 0.4958959 | 0.8979086 | -4.6176793 |
| NCR1.1 | -0.1550889 | 1.73345267 | -0.6849796 | 0.50331404 | 0.89935598 | -4.61838 |
| CD302 | -0.2150929 | 3.63938867 | -0.6846228 | 0.5035332 | 0.89935598 | -4.6184004 |
| WFIKKN1 | -0.1442082 | 1.585504 | -0.6837261 | 0.50408424 | 0.89935598 | -4.6184518 |
| STC1 | 0.29742821 | 5.626566 | 0.68006139 | 0.50633983 | 0.89935598 | -4.618661 |
| Dkk.4 | -0.2312746 | 2.18476733 | -0.6726255 | 0.51093442 | 0.90321583 | -4.6190823 |
| NMNAT1 | -0.2963311 | 8.89081733 | -0.6637354 | 0.51645887 | 0.90491979 | -4.6195807 |
| IL32 | 0.20634357 | 1.84879 | 0.66063989 | 0.51839041 | 0.90491979 | -4.6197528 |
| PPP1R9B | -0.2309782 | 5.05223533 | -0.6543252 | 0.52234333 | 0.90491979 | -4.6201017 |
| CLEC4C | -0.1959996 | 2.003412 | -0.6535883 | 0.52280573 | 0.90491979 | -4.6201422 |
| DCBLD2 | -0.3077414 | 5.307228 | -0.65057 | 0.52470212 | 0.90491979 | -4.6203077 |
| AREG | -0.3431768 | 5.457344 | -0.647786 | 0.52645468 | 0.90491979 | -4.6204598 |
| VEGFR.2 | -0.2687464 | 5.146016 | -0.6409965 | 0.53074252 | 0.90810532 | -4.6208282 |
| LAMP3.1 | -0.1972496 | 2.109474 | -0.6241006 | 0.54149678 | 0.91842928 | -4.6217298 |
| EDA2R | -0.2024175 | 3.445968 | -0.6227585 | 0.54235618 | 0.91842928 | -4.6218004 |
| DCTN1 | -0.1820861 | 8.55627467 | -0.6199401 | 0.54416319 | 0.91842928 | -4.6219484 |
| NDRG1 | -0.102065 | 3.38654333 | -0.6135257 | 0.5482881 | 0.91931021 | -4.6222829 |
| ADA | 0.15874 | 6.40945533 | 0.60915722 | 0.55110699 | 0.91931021 | -4.6225089 |
| AXIN1 | -0.26606 | 4.36183867 | -0.6064256 | 0.55287362 | 0.91931021 | -4.6226495 |
| CCL28 | -0.0883068 | 2.198728 | -0.5969525 | 0.55902374 | 0.91931021 | -4.6231325 |
| Gal.8 | -0.1788521 | 8.547786 | -0.5906442 | 0.56313934 | 0.91931021 | -4.6234502 |
| TBCB | -0.1951021 | 8.29528267 | -0.5900253 | 0.56354398 | 0.91931021 | -4.6234813 |
| ILKAP | -0.1584554 | 6.71198333 | -0.5882119 | 0.5647305 | 0.91931021 | -4.6235719 |
| CLEC4D | -0.191235 | 1.54126267 | -0.5863203 | 0.56596952 | 0.91931021 | -4.6236663 |
| PDGF.R.alpha | -0.2046418 | 5.07817733 | -0.5849518 | 0.56686688 | 0.91931021 | -4.6237343 |
| DDX58 | -0.2095621 | 5.79898867 | -0.5727315 | 0.57491258 | 0.92357565 | -4.6243357 |
| SMPD1 | 0.15016 | 2.77664267 | 0.57101757 | 0.57604576 | 0.92357565 | -4.6244191 |
| CXCL5.1 | -0.1848389 | 4.157954 | -0.5696885 | 0.57692527 | 0.92357565 | -4.6244836 |
| MANF | -0.3706139 | 6.13890333 | -0.5608444 | 0.58279546 | 0.92898593 | -4.6249094 |
| NCAN | -0.2143025 | 4.33208667 | -0.5521212 | 0.58861522 | 0.9303323 | -4.6253233 |
| FGF.21 | -0.2691211 | 2.63970133 | -0.5521014 | 0.58862848 | 0.9303323 | -4.6253242 |
| MDGA1 | -0.1264146 | 1.88388667 | -0.5460434 | 0.59268747 | 0.93279505 | -4.6256082 |
| CCL19.1 | -0.4151393 | 7.30787933 | -0.530969 | 0.60300367 | 0.94318469 | -4.6262908 |
| IL.17A | -0.2209525 | 1.005168 | -0.5264111 | 0.60593736 | 0.94318469 | -4.6265082 |
| TRANCE | -0.1854582 | 3.87758067 | -0.5250301 | 0.60687487 | 0.94318469 | -4.6265703 |
| NPM1 | 0.30677571 | 8.003858 | 0.5201631 | 0.61018451 | 0.94439346 | -4.6267882 |
| TNFRSF9 | -0.1831411 | 4.48321267 | -0.5090735 | 0.61775831 | 0.94560225 | -4.6272774 |
| CADM3 | -0.2056175 | 2.79597 | -0.5085121 | 0.61814295 | 0.94560225 | -4.6273019 |
| AKT1S1 | 0.21250393 | 6.016606 | 0.50498219 | 0.62056392 | 0.94560225 | -4.6274554 |
| CAIX | -0.1768761 | 8.02969933 | -0.4987292 | 0.62486363 | 0.94560225 | -4.6277249 |
| TNFSF14 | -0.2056496 | 2.37373733 | -0.4899472 | 0.63092595 | 0.94560225 | -4.6280979 |
| CNTN5 | 0.13013964 | 2.09713067 | 0.48845366 | 0.63195973 | 0.94560225 | -4.6281608 |
| PRDX5 | -0.1118775 | 8.30354467 | -0.4878782 | 0.63235824 | 0.94560225 | -4.6281849 |
| NCR1 | -0.0999886 | 1.32152067 | -0.4819511 | 0.63646963 | 0.94560225 | -4.6284322 |
| IRAK1 | -0.1838271 | 4.34604867 | -0.4792833 | 0.63832423 | 0.94560225 | -4.6285426 |
| PSG1 | 0.18587214 | 3.53220667 | 0.47769942 | 0.6394265 | 0.94560225 | -4.6286078 |
| CKAP4 | -0.1619857 | 3.90193667 | -0.4746498 | 0.64155124 | 0.94560225 | -4.6287329 |
| JUN | -0.118495 | 1.45470467 | -0.471781 | 0.64355295 | 0.94560225 | -4.6288498 |
| LAMP3 | -0.1103779 | 1.994154 | -0.4686134 | 0.64576648 | 0.94560225 | -4.6289782 |
| HEXIM1 | 0.21292107 | 8.46514133 | 0.46457742 | 0.64859177 | 0.94560225 | -4.6291406 |
| MCP.3.1 | -0.1637543 | 1.289078 | -0.4586718 | 0.65273588 | 0.94560225 | -4.6293757 |
| NBL1 | -0.09393 | 5.41685333 | -0.4568877 | 0.65399021 | 0.94560225 | -4.6294462 |
| IL33.1 | 0.26529643 | 3.428026 | 0.45419327 | 0.65588649 | 0.94560225 | -4.6295522 |
| DSG3 | -0.1172893 | 8.6837 | -0.4503352 | 0.65860599 | 0.94560225 | -4.6297028 |
| SIRT2 | -0.2679407 | 7.48034933 | -0.4495885 | 0.65913294 | 0.94560225 | -4.6297318 |
| ARNT | -0.2718886 | 1.508608 | -0.4398621 | 0.66613877 | 0.94619185 | -4.630097 |
| PIK3AP1 | 0.17342929 | 2.68296067 | 0.43880171 | 0.66676526 | 0.94619185 | -4.630146 |
| PSIP1 | 0.36245179 | 6.02344933 | 0.43702497 | 0.66815067 | 0.94619185 | -4.6302047 |
| IFN.gamma | 0.34782214 | 3.37874467 | 0.43140583 | 0.67214315 | 0.94619185 | -4.630416 |
| IL18.1 | -0.1660704 | 9.322184 | -0.4311149 | 0.67222745 | 0.94619185 | -4.6304352 |
| PHOSPHO1 | -0.0909196 | 4.99262733 | -0.4220049 | 0.67872558 | 0.95174677 | -4.6307717 |
| Gal.9 | -0.1531246 | 6.844904 | -0.4147688 | 0.68390585 | 0.95220396 | -4.631034 |
| EIF4B | -0.087715 | 8.230958 | -0.4144182 | 0.68415727 | 0.95220396 | -4.6310466 |
| KYNU | 0.10485929 | 7.859276 | 0.40390422 | 0.69171445 | 0.95591263 | -4.6314198 |
| SCARB2 | -0.0935471 | 2.985386 | -0.3997173 | 0.69473335 | 0.95591263 | -4.6315658 |
| MMP.1 | -0.1953707 | 6.68538667 | -0.3991062 | 0.69517444 | 0.95591263 | -4.631587 |
| NTF4 | 0.07423643 | 1.66850867 | 0.38767731 | 0.7034441 | 0.95591263 | -4.6319775 |
| EIF4G1 | -0.1095568 | 7.26126933 | -0.384489 | 0.70575801 | 0.95591263 | -4.6320845 |
| HAGH | 0.10910786 | 2.238034 | 0.38167294 | 0.70780429 | 0.95591263 | -4.6321784 |
| CXCL5 | -0.1245311 | 3.395236 | -0.3814816 | 0.70794343 | 0.95591263 | -4.6321847 |
| GM.CSF.R.alpha | 0.14751857 | 4.516196 | 0.3800198 | 0.7090066 | 0.95591263 | -4.6322331 |
| CCL19 | -0.2964154 | 6.50520067 | -0.3779632 | 0.71060946 | 0.95591263 | -4.6322941 |
| FAM3B | -0.1415257 | 2.46356933 | -0.3752918 | 0.71244963 | 0.95591263 | -4.6323884 |
| IL.20 | -0.1098475 | 1.22726333 | -0.3634604 | 0.72109353 | 0.96191622 | -4.6327689 |
| MGMT | 0.0690125 | 7.89820267 | 0.35343633 | 0.72844788 | 0.96191622 | -4.6330821 |
| IL13.1 | -0.1202025 | 1.21567867 | -0.3461723 | 0.73379446 | 0.96191622 | -4.6333037 |
| CCL17 | 0.23260143 | 7.076212 | 0.34542629 | 0.73437481 | 0.96191622 | -4.6333243 |
| EGF | -0.142885 | 2.45633 | -0.3407266 | 0.73781206 | 0.96191622 | -4.6334669 |
| TRAF2 | -0.1106139 | 5.88905133 | -0.3402165 | 0.73818881 | 0.96191622 | -4.633482 |
| ADA.1 | 0.08139429 | 5.809828 | 0.33954324 | 0.73868615 | 0.96191622 | -4.633502 |
| FGFR2 | 0.07495679 | 2.368186 | 0.32692861 | 0.74802662 | 0.96191622 | -4.6338691 |
| HO.1 | -0.177765 | 10.356456 | -0.3250055 | 0.74945418 | 0.96191622 | -4.6339239 |
| ANGPT2 | -0.1029914 | 3.14252067 | -0.3210185 | 0.75241686 | 0.96191622 | -4.6340364 |
| ICA1 | -0.1324671 | 3.99222133 | -0.3198589 | 0.75327927 | 0.96191622 | -4.6340689 |
| CRIP2 | -0.0542461 | 9.43178733 | -0.3173461 | 0.7551493 | 0.96191622 | -4.6341389 |
| CLEC1B | -0.0807164 | 5.10061133 | -0.3135277 | 0.75799392 | 0.96191622 | -4.6342442 |
| TIE2 | -0.0979711 | 4.28769133 | -0.3085426 | 0.7617131 | 0.96191622 | -4.6343799 |
| CD38 | -0.0804614 | 2.66715267 | -0.3076305 | 0.76239428 | 0.96191622 | -4.6344045 |
| IFI30 | -0.0647632 | 4.55556867 | -0.3022854 | 0.76638996 | 0.96191622 | -4.6345471 |
| EZR | -0.0519079 | 6.77741133 | -0.2987325 | 0.76904969 | 0.96191622 | -4.6346406 |
| GZMB | 0.2409675 | 2.52571467 | 0.29416743 | 0.77255227 | 0.96191622 | -4.6347547 |
| NT.3 | 0.06420571 | 1.40743 | 0.29282754 | 0.77347681 | 0.96191622 | -4.6347936 |
| IL.1.alpha.1 | -0.2418257 | 4.59490733 | -0.2917978 | 0.77432966 | 0.96191622 | -4.6348156 |
| SCARA5 | -0.0894471 | 8.81008667 | -0.2793684 | 0.78359751 | 0.96191622 | -4.635131 |
| CCL23 | 0.12270393 | 7.22796867 | 0.27289078 | 0.78848293 | 0.96191622 | -4.6352879 |
| IL33 | 0.08949536 | 1.281152 | 0.27199172 | 0.78916173 | 0.96191622 | -4.6353094 |
| LAIR.2 | -0.1379068 | 4.98779733 | -0.2707023 | 0.79013558 | 0.96191622 | -4.6353401 |
| ITGA11 | -0.0607304 | 1.63231133 | -0.2704573 | 0.79032064 | 0.96191622 | -4.6353459 |
| DAPP1 | -0.1397589 | 4.694372 | -0.2701491 | 0.79055343 | 0.96191622 | -4.6353532 |
| CD4 | 0.08054357 | 2.14838467 | 0.26780338 | 0.79232625 | 0.96191622 | -4.6354085 |
| Gal.1 | -0.0876704 | 6.65992467 | -0.264352 | 0.79493677 | 0.96191622 | -4.6354891 |
| CD83 | -0.0699954 | 1.76900267 | -0.2640825 | 0.79514078 | 0.96191622 | -4.6354954 |
| ST1A1 | 0.11501929 | 1.94166867 | 0.26338605 | 0.79566787 | 0.96191622 | -4.6355115 |
| ZBTB16 | -0.128315 | 4.34092133 | -0.2604205 | 0.79791354 | 0.96191622 | -4.6355797 |
| IL13 | 0.09380821 | 0.472444 | 0.25374763 | 0.80297334 | 0.96191622 | -4.6357303 |
| TRIM5 | 0.09416429 | 4.681386 | 0.25008377 | 0.80575538 | 0.96191622 | -4.6358114 |
| MCP.4 | 0.12351929 | 9.11640867 | 0.25003541 | 0.80579212 | 0.96191622 | -4.6358124 |
| TNFB | -0.0668857 | 3.425338 | -0.2482032 | 0.80718439 | 0.96191622 | -4.6358525 |
| IL4 | 0.11424214 | -0.077628 | 0.24029942 | 0.81319797 | 0.96599632 | -4.6360222 |
| CCL27 | 0.04883143 | 5.73406 | 0.22609162 | 0.82403818 | 0.97027882 | -4.6363135 |
| SRPK2 | 0.11482714 | 6.01826333 | 0.22565499 | 0.82437191 | 0.97027882 | -4.6363222 |
| FHIT | 0.05709643 | 3.23354067 | 0.22416512 | 0.82551094 | 0.97027882 | -4.6363517 |
| MILR1 | -0.0524925 | 1.88823867 | -0.2188658 | 0.82956561 | 0.97027882 | -4.636455 |
| IL.1.alpha | -0.1847896 | 3.754054 | -0.2155015 | 0.83220052 | 0.97027882 | -4.6365168 |
| CCL23.1 | 0.09260286 | 7.605724 | 0.2147304 | 0.83273322 | 0.97027882 | -4.6365339 |
| ADAM.23 | 0.057335 | 1.679288 | 0.2117577 | 0.83501207 | 0.97027882 | -4.6365897 |
| GGT5 | -0.0410079 | 2.42460733 | -0.2078465 | 0.83801268 | 0.97074139 | -4.6366619 |
| TNF | -0.1542693 | 3.19042467 | -0.197218 | 0.84623266 | 0.97085839 | -4.6368494 |
| PTH1R | -0.0587336 | 2.125702 | -0.1968968 | 0.84642671 | 0.97085839 | -4.6368571 |
| CCL20.1 | -0.1835482 | 3.701202 | -0.1937685 | 0.84888628 | 0.97085839 | -4.6369089 |
| IL12 | -0.1240443 | 5.21435733 | -0.1888348 | 0.85263414 | 0.97085839 | -4.6369941 |
| CPM | -0.0723261 | 6.185972 | -0.1859073 | 0.85489078 | 0.97085839 | -4.6370424 |
| SPRY2 | 0.04882393 | 1.72181133 | 0.18401098 | 0.85635316 | 0.97085839 | -4.6370733 |
| MMP7 | -0.0595139 | 11.6912907 | -0.1838387 | 0.85648605 | 0.97085839 | -4.6370761 |
| COL4A3BP | 0.0894825 | 3.175458 | 0.18066303 | 0.85893637 | 0.97085839 | -4.6371272 |
| PFDN2 | -0.0381846 | 2.37961267 | -0.1714499 | 0.86605351 | 0.97359498 | -4.6372702 |
| VWC2 | -0.0756936 | 3.91596933 | -0.1675689 | 0.86905517 | 0.97359498 | -4.6373282 |
| CARHSP1 | -0.0471921 | 3.31112933 | -0.1673972 | 0.86918801 | 0.97359498 | -4.6373308 |
| DCN | -0.0396236 | 4.862266 | -0.158514 | 0.87606648 | 0.97685417 | -4.6374585 |
| MCP.4.1 | 0.07802429 | 13.935558 | 0.1506919 | 0.88213188 | 0.97685417 | -4.6375652 |
| MAPT | -0.1292214 | 2.890688 | -0.1445461 | 0.88694129 | 0.97685417 | -4.6376441 |
| TNFRSF4 | -0.0550496 | 4.02166933 | -0.1395695 | 0.89076936 | 0.97685417 | -4.6377077 |
| CDH6 | 0.04161107 | 2.53549133 | 0.13654896 | 0.89311755 | 0.97685417 | -4.6377445 |
| TPPP3 | -0.0243304 | 7.50903933 | -0.1362024 | 0.89338702 | 0.97685417 | -4.6377487 |
| IL.12B | -0.0823071 | 4.95215467 | -0.1343274 | 0.89484525 | 0.97685417 | -4.6377711 |
| IKZF2 | -0.04434 | 2.40361733 | -0.1318385 | 0.89678156 | 0.97685417 | -4.6378004 |
| IL18 | -0.0538614 | 9.10684733 | -0.130696 | 0.89767062 | 0.97685417 | -4.6378136 |
| FKBP5 | 0.0256825 | 8.30480933 | 0.12990427 | 0.89828681 | 0.97685417 | -4.6378227 |
| GDNF | 0.02507143 | 1.40385667 | 0.12501337 | 0.90209474 | 0.97777363 | -4.6378778 |
| DRAXIN | 0.04492714 | 2.67732933 | 0.12144098 | 0.90487769 | 0.97777363 | -4.6379167 |
| SCF | 0.03213429 | 7.066144 | 0.11636846 | 0.90883145 | 0.97777363 | -4.63797 |
| CXCL11.1 | -0.0827679 | 3.53159067 | -0.1151315 | 0.90982656 | 0.97777363 | -4.6379819 |
| ITGB6 | -0.0276225 | 1.733256 | -0.1070959 | 0.9160653 | 0.97777363 | -4.6380615 |
| TNFRSF12A.1 | -0.0286825 | 2.19654333 | -0.1069356 | 0.91619038 | 0.97777363 | -4.6380631 |
| GZMH | -0.0402711 | 1.446484 | -0.1047566 | 0.91789145 | 0.97777363 | -4.6380835 |
| GCP5 | 0.03472893 | 2.11501333 | 0.10192406 | 0.92010333 | 0.97777363 | -4.6381093 |
| CXCL11 | 0.0717325 | 2.90605 | 0.09527911 | 0.92532015 | 0.9788953 | -4.6381667 |
| ANGPT1 | -0.0210893 | 2.685086 | -0.0853085 | 0.93309117 | 0.9788953 | -4.6382469 |
| LXN | 0.01871536 | 1.73456867 | 0.08495525 | 0.93336753 | 0.9788953 | -4.6382495 |
| NAAA | -0.0341054 | 3.161468 | -0.0826627 | 0.93516124 | 0.9788953 | -4.6382665 |
| IL12.1 | 0.04945286 | 6.298844 | 0.08093688 | 0.93651175 | 0.9788953 | -4.638279 |
| CLEC4A | 0.01858821 | 2.291828 | 0.07905194 | 0.93798701 | 0.9788953 | -4.6382923 |
| IRAK4 | -0.0296239 | 6.409184 | -0.0748933 | 0.94124267 | 0.9788953 | -4.6383206 |
| TMPRSS5 | -0.0144164 | 1.53344467 | -0.0737295 | 0.94215392 | 0.9788953 | -4.6383283 |
| SH2B3 | -0.018735 | 1.37384867 | -0.0412562 | 0.96761012 | 0.99230445 | -4.6384935 |
| PVR | 0.01193071 | 5.16264267 | 0.04092506 | 0.96786996 | 0.99230445 | -4.6384947 |
| CXCL9 | 0.031125 | 5.48359467 | 0.04058385 | 0.96814841 | 0.99230445 | -4.6384958 |
| SRP14 | -0.0200889 | 5.842748 | -0.0385901 | 0.96970215 | 0.99230445 | -4.6385029 |
| EGLN1 | 0.01332536 | 9.17908867 | 0.03850092 | 0.9697721 | 0.99230445 | -4.6385032 |
| CXCL9.1 | -0.0278864 | 6.21711933 | -0.0369209 | 0.97102178 | 0.99230445 | -4.6385084 |
| TNFRSF12A | -0.0078068 | 2.01245533 | -0.0280385 | 0.97798369 | 0.99619398 | -4.638534 |
| IRF9 | -0.0145204 | 4.17827867 | -0.0252542 | 0.98016942 | 0.99619398 | -4.6385406 |
| KPNA1 | -0.0080482 | -0.1835153 | -0.0151369 | 0.98811302 | 0.99864209 | -4.6385587 |
| IL4.1 | -0.0083204 | 0.29341333 | -0.0144869 | 0.98862349 | 0.99864209 | -4.6385595 |
| PRDX1 | 0.00257607 | 7.59865133 | 0.00913558 | 0.99282568 | 0.99864209 | -4.6385651 |
| CXCL10.1 | -0.0058136 | 8.93675333 | -0.0062573 | 0.99508769 | 0.99864209 | -4.6385671 |
| CXCL10 | -0.0020079 | 7.92039067 | -0.0021574 | 0.99830628 | 0.99864209 | -4.6385686 |
| CCL20 | -0.0016893 | 3.52175933 | -0.0017297 | 0.99864209 | 0.99864209 | -4.6385687 |

**Supplementary Table 3: Differential protein expression as detected by a proteomic multiplex assay from suction blister fluid,** given as log2 fold change (FCH) of spontaneously healed atopic dermatitis (n=4) over healthy control skin (n=7); adjusted p values were calculated using a linear mixed model with Benjamini-Hochberg correction for multiple testing.

| **Gene name** | **p_val** | **avg_logFC** | **pct.1** | **pct.2** | **p_val_adj** | **Gene** | **Cluster label** |
| --- | --- | --- | --- | --- | --- | --- | --- |
| NEAT1 | 1.26E-09 | 0.44967093 | 0.645 | 0.97 | 4.23E-05 | NEAT1 | KC-1 |
| MALAT1 | 3.09E-08 | -0.765825 | 0.723 | 0.999 | 0.00103583 | MALAT1 | KC-1 |
| DDX5 | 6.78E-08 | 0.61330016 | 0.809 | 0.797 | 0.00227346 | DDX5 | KC-1 |
| DDX5 | 6.82E-08 | 0.36050815 | 0.721 | 0.673 | 0.00228875 | DDX5 | KC-2 |
| HBB | 2.99E-07 | -0.6384742 | 0.016 | 0.162 | 0.01003196 | HBB | KC-2 |
| SNHG25 | 1.34E-10 | -0.2804284 | 0.181 | 0.313 | 4.50E-06 | SNHG25 | KC-4 |
| CRIP1 | 1.02E-06 | -0.6624768 | 0.574 | 0.747 | 0.0342464 | CRIP1 | KC-4 |
| SNHG25 | 9.31E-11 | -0.2933392 | 0.053 | 0.239 | 3.12E-06 | SNHG25 | DC |
| HLA-DRB5 | 1.12E-10 | -0.6342661 | 0.624 | 0.845 | 3.77E-06 | HLA-DRB5 | DC |
| NEAT1 | 4.11E-10 | -0.3163401 | 0.577 | 0.787 | 1.38E-05 | NEAT1 | DC |
| GABARAP | 7.93E-10 | -0.4719409 | 0.238 | 0.387 | 2.66E-05 | GABARAP | DC |
| FAM133B | 3.53E-09 | -0.2790845 | 0.429 | 0.413 | 0.00011823 | FAM133B | DC |
| TPP1 | 3.92E-09 | -0.3428582 | 0.365 | 0.465 | 0.0001314 | TPP1 | DC |
| HSPA1A | 1.49E-08 | -0.2881882 | 0.455 | 0.555 | 0.00050002 | HSPA1A | DC |
| FAM118A | 2.98E-08 | -0.2940641 | 0.228 | 0.284 | 0.00099932 | FAM118A | DC |
| MALAT1 | 4.71E-08 | -0.846791 | 0.624 | 0.968 | 0.00157968 | MALAT1 | DC |
| SORL1 | 7.30E-08 | -0.2562597 | 0.143 | 0.219 | 0.00244709 | SORL1 | DC |
| MEF2C | 1.24E-07 | -0.3987418 | 0.349 | 0.503 | 0.00415649 | MEF2C | DC |
| ZFHX3 | 2.07E-07 | -0.2865475 | 0.249 | 0.284 | 0.00695515 | ZFHX3 | DC |
| TNFRSF1B | 2.56E-07 | -0.3872409 | 0.376 | 0.503 | 0.00859689 | TNFRSF1B | DC |
| ANKRD11 | 2.60E-07 | -0.273792 | 0.407 | 0.381 | 0.00872975 | ANKRD11 | DC |
| CD74 | 1.71E-12 | -0.3579569 | 1 | 1 | 5.72E-08 | CD74 | LC |
| HLA-DRB5 | 8.92E-12 | -1.0999724 | 0.723 | 0.912 | 2.99E-07 | HLA-DRB5 | LC |
| CRIP1 | 9.43E-10 | -0.9938253 | 0.733 | 0.851 | 3.16E-05 | CRIP1 | LC |
| BNIP3 | 4.24E-08 | -0.3727127 | 0.099 | 0.293 | 0.00142116 | BNIP3 | LC |
| MTRNR2L8 | 1.80E-07 | 0.26405637 | 0.663 | 0.859 | 0.00604314 | MTRNR2L8 | LC |
| AC114760.2 | 5.32E-07 | -0.4532154 | 0.277 | 0.39 | 0.01782694 | AC114760.2 | LC |
| DDX5 | 9.72E-07 | 0.43545341 | 0.941 | 0.775 | 0.03258791 | DDX5 | LC |
| DDX5 | 1.50E-19 | 0.56411416 | 0.986 | 0.616 | 5.02E-15 | DDX5 | MEL |
| DCT | 4.05E-19 | -0.2672681 | 1 | 1 | 1.36E-14 | DCT | MEL |
| TXNIP | 4.62E-19 | 0.95862411 | 0.936 | 0.288 | 1.55E-14 | TXNIP | MEL |
| HLA-DPA1 | 7.47E-19 | 0.48882327 | 0.95 | 0.546 | 2.51E-14 | HLA-DPA1 | MEL |
| NEAT1 | 8.74E-18 | 0.30721592 | 0.993 | 0.779 | 2.93E-13 | NEAT1 | MEL |
| EMP1 | 2.27E-17 | -0.2588514 | 0.9 | 0.74 | 7.63E-13 | EMP1 | MEL |
| ATP1B1 | 1.92E-15 | 1.12039438 | 0.936 | 0.36 | 6.45E-11 | ATP1B1 | MEL |
| MIA | 9.84E-15 | -0.3776917 | 0.386 | 0.368 | 3.30E-10 | MIA | MEL |
| ZFAS1 | 3.34E-14 | -0.304151 | 0.9 | 0.72 | 1.12E-09 | ZFAS1 | MEL |
| APOE | 5.07E-14 | -0.4859509 | 1 | 0.998 | 1.70E-09 | APOE | MEL |
| GMPR | 7.02E-14 | 0.76692721 | 0.993 | 0.618 | 2.36E-09 | GMPR | MEL |
| DPYSL2 | 5.98E-13 | 0.33408808 | 0.893 | 0.372 | 2.01E-08 | DPYSL2 | MEL |
| ATF3 | 9.36E-13 | -0.5205262 | 0.5 | 0.536 | 3.14E-08 | ATF3 | MEL |
| PABPC4 | 1.99E-12 | 0.29342812 | 0.643 | 0.168 | 6.67E-08 | PABPC4 | MEL |
| PRNP | 6.41E-12 | 0.40508698 | 0.757 | 0.174 | 2.15E-07 | PRNP | MEL |
| MFSD12 | 2.94E-11 | 0.88494822 | 1 | 0.906 | 9.87E-07 | MFSD12 | MEL |
| MTRNR2L8 | 5.01E-11 | 0.50827136 | 0.757 | 0.628 | 1.68E-06 | MTRNR2L8 | MEL |
| PLA2G7 | 9.29E-11 | 0.37509616 | 0.771 | 0.254 | 3.11E-06 | PLA2G7 | MEL |
| CHADL | 1.27E-10 | 1.07461295 | 0.743 | 0.096 | 4.24E-06 | CHADL | MEL |
| TPT1 | 2.94E-10 | 0.36690078 | 1 | 0.969 | 9.86E-06 | TPT1 | MEL |
| TPPP3 | 4.84E-10 | -0.479876 | 0.979 | 0.93 | 1.62E-05 | TPPP3 | MEL |
| EEF1A1 | 3.00E-09 | 0.34591199 | 1 | 0.998 | 0.00010054 | EEF1A1 | MEL |
| HBB | 3.71E-09 | -1.645607 | 0.079 | 0.184 | 0.00012441 | HBB | MEL |
| RACK1 | 3.96E-09 | -0.4660439 | 1 | 0.992 | 0.0001328 | RACK1 | MEL |
| EMP3 | 4.28E-09 | -0.3133407 | 1 | 0.996 | 0.00014347 | EMP3 | MEL |
| NSMF | 4.37E-09 | 0.39883576 | 0.529 | 0.104 | 0.00014657 | NSMF | MEL |
| ATP6V0E2 | 1.15E-08 | 0.41747641 | 0.757 | 0.254 | 0.00038653 | ATP6V0E2 | MEL |
| CRABP1 | 1.56E-08 | 0.38757029 | 0.993 | 0.912 | 0.00052177 | CRABP1 | MEL |
| HSPB1 | 4.03E-08 | -0.9160969 | 1 | 1 | 0.00135209 | HSPB1 | MEL |
| PTGDS | 7.86E-08 | 1.44640212 | 0.5 | 0.194 | 0.00263537 | PTGDS | MEL |
| CYB561A3 | 9.13E-08 | 0.91160668 | 0.993 | 0.836 | 0.00306047 | CYB561A3 | MEL |
| COX7C | 1.17E-07 | -0.3635667 | 0.986 | 0.918 | 0.00393846 | COX7C | MEL |
| DKK3 | 2.34E-07 | 0.77001692 | 0.807 | 0.231 | 0.0078389 | DKK3 | MEL |
| TIMP1 | 2.67E-07 | -0.5412041 | 0.571 | 0.613 | 0.00895308 | TIMP1 | MEL |
| SGK1 | 2.84E-07 | 0.40031578 | 0.879 | 0.425 | 0.00951064 | SGK1 | MEL |
| CALU | 3.18E-07 | 0.3062811 | 0.743 | 0.272 | 0.01065559 | CALU | MEL |
| LMNA | 4.40E-07 | -0.4510173 | 1 | 0.988 | 0.01474044 | LMNA | MEL |
| DOCK5 | 5.40E-07 | 0.4637295 | 0.707 | 0.219 | 0.01809991 | DOCK5 | MEL |
| TUBA1B | 8.01E-07 | -0.4252734 | 0.979 | 0.947 | 0.02685219 | TUBA1B | MEL |
| GNAL | 1.00E-06 | 0.60307567 | 0.729 | 0.176 | 0.03354201 | GNAL | MEL |
| ID4 | 1.19E-06 | 0.27684122 | 0.5 | 0.17 | 0.03992312 | ID4 | MEL |
| CD81 | 1.38E-06 | 1.51697155 | 1 | 0.419 | 0.04624672 | CD81 | MEL |
| TYR | 1.46E-06 | 0.28490269 | 1 | 0.912 | 0.04910032 | TYR | MEL |
| CTSW | 3.97E-21 | -0.4335358 | 0.599 | 0.459 | 1.33E-16 | CTSW | TC-1 |
| LINC01871 | 1.89E-19 | -0.8333522 | 0.467 | 0.698 | 6.32E-15 | LINC01871 | TC-1 |
| XIST | 2.50E-19 | 0.37787681 | 0.371 | 0.09 | 8.40E-15 | XIST | TC-1 |
| GPX4 | 1.10E-13 | 0.26214872 | 0.939 | 0.701 | 3.70E-09 | GPX4 | TC-1 |
| ZFP36L2 | 4.46E-10 | 0.3051282 | 0.995 | 0.791 | 1.50E-05 | ZFP36L2 | TC-1 |
| MALAT1 | 1.15E-09 | -0.3408585 | 1 | 1 | 3.85E-05 | MALAT1 | TC-1 |
| PABPC1 | 2.40E-09 | 0.29161264 | 0.959 | 0.594 | 8.05E-05 | PABPC1 | TC-1 |
| IL27RA | 5.30E-09 | 0.63623929 | 0.629 | 0.177 | 0.0001779 | IL27RA | TC-1 |
| BIRC3 | 6.52E-09 | 0.6089939 | 0.878 | 0.454 | 0.00021872 | BIRC3 | TC-1 |
| PRDX1 | 1.71E-08 | 0.47872642 | 0.964 | 0.743 | 0.000574 | PRDX1 | TC-1 |
| TNFRSF4 | 2.62E-08 | 0.6541508 | 0.665 | 0.342 | 0.00087826 | TNFRSF4 | TC-1 |
| TGFB1 | 3.79E-08 | 0.80466861 | 0.944 | 0.449 | 0.00127125 | TGFB1 | TC-1 |
| NINJ1 | 9.59E-08 | 0.45623339 | 0.452 | 0.155 | 0.00321601 | NINJ1 | TC-1 |
| CRIP1 | 1.26E-07 | -0.7047794 | 0.817 | 0.726 | 0.00422273 | CRIP1 | TC-1 |
| SON | 1.41E-07 | -0.4273601 | 0.792 | 0.738 | 0.00473916 | SON | TC-1 |
| PET100 | 1.42E-07 | -0.4455664 | 0.538 | 0.559 | 0.0047476 | PET100 | TC-1 |
| HSPA1A | 1.50E-07 | -0.2933277 | 0.416 | 0.304 | 0.00502289 | HSPA1A | TC-1 |
| TAPBP | 3.05E-07 | 0.69887745 | 0.807 | 0.302 | 0.01021712 | TAPBP | TC-1 |
| HLA-DQA2 | 4.02E-07 | 0.41061137 | 0.178 | 0.01 | 0.01349738 | HLA-DQA2 | TC-1 |
| CD320 | 4.57E-07 | -0.3264402 | 0.061 | 0.18 | 0.01531578 | CD320 | TC-1 |
| IRF1 | 7.60E-07 | 0.71025327 | 0.665 | 0.269 | 0.02549531 | IRF1 | TC-1 |
| HBB | 9.99E-07 | -0.9100901 | 0.041 | 0.227 | 0.03349186 | HBB | TC-1 |
| LIMD2 | 1.20E-06 | 0.3030142 | 0.746 | 0.399 | 0.04029278 | LIMD2 | TC-1 |
| CD8B | 7.76E-13 | 0.78269501 | 0.872 | 0.315 | 2.60E-08 | CD8B | TC-2 |
| CD8A | 1.06E-10 | 1.16380721 | 0.936 | 0.425 | 3.56E-06 | CD8A | TC-2 |
| TRDC | 8.54E-09 | -0.9540185 | 0.007 | 0.197 | 0.00028633 | TRDC | TC-2 |
| KLRD1 | 1.96E-08 | -1.1844334 | 0.099 | 0.331 | 0.00065889 | KLRD1 | TC-2 |
| KLF2 | 1.09E-07 | -0.3477454 | 0.078 | 0.157 | 0.00364663 | KLF2 | TC-2 |
| LMO4 | 1.82E-07 | -0.469367 | 0.106 | 0.236 | 0.00611218 | LMO4 | TC-2 |
| KLRF1 | 2.85E-07 | -0.9934207 | 0.014 | 0.197 | 0.00956425 | KLRF1 | TC-2 |
| HLA-DRB1 | 3.21E-07 | 0.36855077 | 0.95 | 0.661 | 0.01077828 | HLA-DRB1 | TC-2 |
| KLRB1 | 3.58E-07 | -1.1694529 | 0.071 | 0.268 | 0.01201137 | KLRB1 | TC-2 |
| CD69 | 5.63E-07 | 0.36829778 | 0.915 | 0.528 | 0.01888986 | CD69 | TC-2 |
| CCL5 | 1.20E-06 | 0.45722628 | 1 | 0.89 | 0.04031506 | CCL5 | TC-2 |
| XIST | 4.08E-07 | 0.37613929 | 0.293 | 0.056 | 0.01367216 | XIST | TC-3 |
| MTRNR2L8 | 2.05E-23 | -0.9476829 | 0.219 | 0.562 | 6.88E-19 | MTRNR2L8 | TC-4 |
| MALAT1 | 7.71E-11 | -0.535138 | 0.982 | 1 | 2.59E-06 | MALAT1 | TC-4 |
| XIST | 1.47E-10 | 0.27794675 | 0.482 | 0.105 | 4.95E-06 | XIST | TC-4 |
| HBB | 1.83E-10 | -0.811717 | 0.018 | 0.267 | 6.15E-06 | HBB | TC-4 |
| NEAT1 | 9.58E-10 | -0.3472475 | 0.684 | 0.514 | 3.21E-05 | NEAT1 | TC-4 |
| CD3E | 1.27E-08 | 0.30962456 | 0.553 | 0.248 | 0.00042678 | CD3E | TC-4 |
| HLA-DQB1 | 3.24E-07 | -0.666079 | 0.158 | 0.381 | 0.01087889 | HLA-DQB1 | TC-4 |
| KLRC1 | 8.99E-07 | -0.642314 | 0.202 | 0.419 | 0.03014551 | KLRC1 | TC-4 |
| SNHG25 | 1.59E-12 | -0.9326362 | 0.021 | 0.364 | 5.32E-08 | SNHG25 | TC-5 |
| LMNA | 1.90E-10 | 0.94442428 | 0.745 | 0.252 | 6.37E-06 | LMNA | TC-5 |
| TXNIP | 6.89E-10 | -0.2892669 | 0.553 | 0.589 | 2.31E-05 | TXNIP | TC-5 |
| GIMAP4 | 1.72E-09 | -0.8471052 | 0.255 | 0.449 | 5.77E-05 | GIMAP4 | TC-5 |
| MTRNR2L8 | 1.48E-08 | -0.4358203 | 0.213 | 0.393 | 0.00049569 | MTRNR2L8 | TC-5 |
| GIMAP7 | 3.31E-08 | -1.0391793 | 0.362 | 0.551 | 0.0011096 | GIMAP7 | TC-5 |
| CREM | 1.35E-07 | 0.91337164 | 0.702 | 0.178 | 0.00451246 | CREM | TC-5 |
| RIPOR2 | 1.72E-07 | -0.4979997 | 0.34 | 0.355 | 0.00575984 | RIPOR2 | TC-5 |
| GIMAP1 | 3.58E-07 | -0.6276344 | 0.17 | 0.355 | 0.01200194 | GIMAP1 | TC-5 |
| RGS1 | 7.46E-07 | 0.31120363 | 0.638 | 0.243 | 0.02501578 | RGS1 | TC-5 |
| DUSP4 | 9.15E-07 | 0.83260846 | 0.426 | 0.093 | 0.03068181 | DUSP4 | TC-5 |
| SYTL3 | 1.12E-06 | 1.48810148 | 0.617 | 0.112 | 0.03764677 | SYTL3 | TC-5 |
| REL | 1.37E-06 | 1.02771058 | 0.723 | 0.187 | 0.04610769 | REL | TC-5 |
| HLA-DRB5 | 5.82E-10 | -0.3520269 | 0.398 | 0.2 | 1.95E-05 | HLA-DRB5 | TREG |
| XIST | 5.91E-09 | 0.26635236 | 0.414 | 0.1 | 0.00019826 | XIST | TREG |
| PSMB3 | 7.13E-08 | 0.25866069 | 0.891 | 0.52 | 0.00239091 | PSMB3 | TREG |
| HBB | 1.37E-06 | -0.8130269 | 0.039 | 0.24 | 0.04592532 | HBB | TREG |

**Supplementary Table 4: Differentially expressed genes of single-cell RNA sequencing analyses,** comparing spontaneously healed atopic dermatitis with healthy control samples for each cluster. Differential gene expression was defined by average log fold change (avg logFC) >⏐0.25⏐ and adjusted p-value<0.05, using logistic regression and Bonferroni correction. pct.1/2: The percentage of cells in which the feature is detected in the first/second group.

| **Protein name** | **logFC** | **AveExpr** | **t** | **P.Value** | **adj.P.Val** | **B** |
| --- | --- | --- | --- | --- | --- | --- |
| KYNU | -2.2130825 | 7.859276 | -7.5561648 | 1.2736E-06 | 0.00030329 | 5.68645137 |
| MMP.10 | -2.3021425 | 5.07359333 | -7.4096134 | 1.6262E-06 | 0.00030329 | 5.45285865 |
| PRTFDC1 | -1.9220975 | 2.077094 | -6.2472083 | 1.2524E-05 | 0.00155718 | 3.4857305 |
| CXCL1.1 | -3.07219 | 7.049578 | -5.82328 | 2.7607E-05 | 0.00240008 | 2.7180263 |
| PSG1 | 2.5208175 | 3.53220667 | 5.74267366 | 3.2173E-05 | 0.00240008 | 2.56911453 |
| MMP.1 | -3.0983575 | 6.68538667 | -5.6103872 | 4.1436E-05 | 0.00257593 | 2.32275192 |
| N2DL.2 | -2.523535 | 4.44294 | -5.5271573 | 4.8644E-05 | 0.00259203 | 2.16651224 |
| IL.20 | -1.8122575 | 1.22726333 | -5.3151951 | 7.3479E-05 | 0.00341745 | 1.76443235 |
| IL13.1 | -2.0476375 | 1.21567867 | -5.2271416 | 8.7358E-05 | 0.00341745 | 1.59569216 |
| CXCL1 | -2.79389 | 5.19560733 | -5.2029801 | 9.1621E-05 | 0.00341745 | 1.54922081 |
| CCL17 | -3.84701 | 7.076212 | -5.0640577 | 0.00012313 | 0.00383978 | 1.26434637 |
| MCP.3 | -2.26211 | 1.03565067 | -5.0522303 | 0.00012353 | 0.00383978 | 1.25767668 |
| MCP.3.1 | -1.9967975 | 1.289078 | -4.9576487 | 0.00014921 | 0.00428121 | 1.07340371 |
| GZMB | -4.3235575 | 2.52571467 | -4.6785327 | 0.00027654 | 0.00685913 | 0.48193314 |
| PTS | -1.764445 | 5.586348 | -4.6253623 | 0.00029199 | 0.00685913 | 0.41849678 |
| DSG3 | -1.35795 | 8.6837 | -4.6216138 | 0.00029423 | 0.00685913 | 0.41104796 |
| FKBP5 | -0.988545 | 8.30480933 | -4.4321546 | 0.00043361 | 0.00951398 | 0.03303942 |
| AREG | -2.5148525 | 5.457344 | -4.2078329 | 0.00068903 | 0.01427814 | -0.4178243 |
| MAD1L1 | -1.31761 | 4.82653467 | -3.8931542 | 0.00132692 | 0.02584983 | -1.0541833 |
| DEFB4A | -4.39314 | 0.08940067 | -3.8892063 | 0.00138605 | 0.02584983 | -1.0875897 |
| AARSD1 | -0.837775 | 6.605638 | -3.7985354 | 0.00161742 | 0.02805647 | -1.2459131 |
| MCP.4.1 | -2.21171 | 13.935558 | -3.7863487 | 0.00165923 | 0.02805647 | -1.2706078 |
| OPG | -1.7620025 | 7.30271067 | -3.7664001 | 0.00173002 | 0.02805647 | -1.3110292 |
| CETN2 | -1.3436625 | 2.93629267 | -3.6854568 | 0.00204986 | 0.03185823 | -1.474995 |
| NAA10 | -1.1563975 | 7.49264067 | -3.5601644 | 0.0026659 | 0.03977526 | -1.7284894 |
| CASP.8.1 | -1.04388 | 4.90855533 | -3.5034159 | 0.0030029 | 0.04308002 | -1.8431034 |
| MCP.4 | -1.939785 | 9.11640867 | -3.4805884 | 0.00315016 | 0.04351894 | -1.8891613 |
| EREG | -1.47399 | 4.43716333 | -3.4537345 | 0.00333266 | 0.04439575 | -1.9433045 |
| TRIM21 | -0.8584925 | 2.351604 | -3.3739698 | 0.00393918 | 0.04903399 | -2.1038465 |
| IL15.1 | -0.863975 | 1.99603267 | -3.3642169 | 0.0040205 | 0.04903399 | -2.1234439 |
| PTPN1 | -1.24851 | 6.52158533 | -3.3481898 | 0.00415777 | 0.04903399 | -2.155632 |
| NFATC3 | -0.811995 | 1.378208 | -3.3426075 | 0.00420667 | 0.04903399 | -2.1668382 |
| CCL23.1 | -1.6151575 | 7.605724 | -3.3198339 | 0.00441214 | 0.04987055 | -2.2125276 |
| IL6.2 | -3.55419 | 4.45151933 | -3.2400082 | 0.00533237 | 0.05749918 | -2.3866057 |
| IL13 | -1.3444825 | 0.472444 | -3.2236553 | 0.00539537 | 0.05749918 | -2.4049436 |
| DDX58 | -1.318155 | 5.79898867 | -3.1932805 | 0.00574877 | 0.05956369 | -2.465509 |
| PSME1 | -0.92897 | 6.41961867 | -3.153502 | 0.00624635 | 0.06026753 | -2.5446606 |
| ADAM15 | -0.75427 | 7.322374 | -3.1409816 | 0.00641157 | 0.06026753 | -2.5695332 |
| MCP.1.1 | -1.7323125 | 11.3488527 | -3.1209892 | 0.00668437 | 0.06026753 | -2.6092073 |
| LAIR.2 | -1.78955 | 4.98779733 | -3.1137405 | 0.00678608 | 0.06026753 | -2.6235789 |
| IL6 | -3.04696 | 3.38274733 | -3.1190175 | 0.00684967 | 0.06026753 | -2.6256831 |
| IL15 | -1.1514975 | 3.81005733 | -3.1120697 | 0.00680974 | 0.06026753 | -2.6268904 |
| HGF | 1.5844225 | 8.18112467 | 3.10178894 | 0.00695711 | 0.06026753 | -2.647259 |
| IL.17A | -1.4638475 | 1.005168 | -3.0913934 | 0.00710931 | 0.06026753 | -2.6678401 |
| TBCB | -1.1399625 | 8.29528267 | -3.0558462 | 0.00765489 | 0.06345056 | -2.7380998 |
| IL6.1 | -3.2269075 | 3.34373267 | -3.053103 | 0.00784801 | 0.06363708 | -2.7551753 |
| GFR.alpha.1 | 1.1681425 | 8.46002067 | 3.01937486 | 0.00825734 | 0.06553166 | -2.8099896 |
| JUN | -0.84402 | 1.45470467 | -2.9786913 | 0.00898425 | 0.0698151 | -2.8899331 |
| MUC.16 | -2.099405 | 3.552174 | -2.9677549 | 0.0091901 | 0.06995729 | -2.9113762 |
| ICA1 | -1.3551125 | 3.99222133 | -2.9004005 | 0.01056326 | 0.07880191 | -3.0429735 |
| HGF.1 | 1.37974 | 8.81748667 | 2.86329917 | 0.01140303 | 0.08063904 | -3.1151006 |
| uPA | -1.0508975 | 8.403514 | -2.8610166 | 0.01145677 | 0.08063904 | -3.1195293 |
| CASP.8 | -0.9390825 | 6.91289933 | -2.8609603 | 0.01145809 | 0.08063904 | -3.1196385 |
| CCL23 | -1.437405 | 7.22796867 | -2.8336217 | 0.01212117 | 0.0825524 | -3.1725986 |
| IL.1.alpha | 2.730295 | 3.754054 | 2.82237351 | 0.01259951 | 0.0825524 | -3.2031035 |
| PIK3AP1 | 1.250965 | 2.68296067 | 2.80558455 | 0.01283984 | 0.0825524 | -3.2267507 |
| MCP.1 | -1.582665 | 10.5260993 | -2.7959209 | 0.01309698 | 0.0825524 | -3.2453767 |
| EGLN1 | -1.0904425 | 9.17908867 | -2.79272 | 0.01318325 | 0.0825524 | -3.2515417 |
| CXCL11.1 | -2.264065 | 3.53159067 | -2.7916032 | 0.01341493 | 0.0825524 | -3.2620967 |
| PPP3R1 | -0.8105625 | 4.78560267 | -2.7816034 | 0.01348716 | 0.0825524 | -3.2729351 |
| PFDN2 | -0.6987775 | 2.37961267 | -2.7811201 | 0.01350053 | 0.0825524 | -3.2738645 |
| IL.1.alpha.1 | 2.59618 | 4.59490733 | 2.7768136 | 0.01382485 | 0.08317209 | -3.2903809 |
| PD.L1.1 | -0.83916 | 4.11626267 | -2.6757522 | 0.01674122 | 0.09911867 | -3.4752102 |
| SNCG | 2.2112 | 4.22956667 | 2.65059409 | 0.01785238 | 0.10404591 | -3.529756 |
| CXCL5 | 0.9702225 | 3.395236 | 2.63450787 | 0.01820371 | 0.10446131 | -3.5532778 |
| CXADR | -0.96219 | 2.70476667 | -2.6161456 | 0.01889357 | 0.10531322 | -3.5878902 |
| ANGPT1 | 0.728365 | 2.685086 | 2.61163153 | 0.01906696 | 0.10531322 | -3.5963852 |
| IL.17C | -1.9703825 | 2.18890533 | -2.6144721 | 0.01919919 | 0.10531322 | -3.5975478 |
| Siglec.9 | -0.8194725 | 3.09519267 | -2.6009033 | 0.01948518 | 0.10533293 | -3.6165523 |
| TRAF2 | -0.9404025 | 5.88905133 | -2.5638443 | 0.0209984 | 0.11189149 | -3.6859699 |
| CDH3 | -1.1426975 | 7.12423 | -2.545168 | 0.02180292 | 0.11454212 | -3.7208054 |
| TNFRSF9.1 | -0.9650325 | 5.30925133 | -2.5306972 | 0.02244641 | 0.11513782 | -3.7477266 |
| CDCP1 | -1.0889175 | 6.790348 | -2.5287651 | 0.02253368 | 0.11513782 | -3.7513162 |
| KIF1BP | -0.5545825 | 6.43904067 | -2.4644938 | 0.0256278 | 0.12917795 | -3.8700803 |
| CLEC7A | -1.300745 | 3.18832 | -2.4527041 | 0.02623748 | 0.13048772 | -3.8917256 |
| PDCD1 | -0.67085 | 2.04571667 | -2.4400278 | 0.02690831 | 0.13191539 | -3.9149484 |
| IFNL1 | -0.63451 | 1.129902 | -2.4280096 | 0.02755928 | 0.13191539 | -3.9369172 |
| BIRC2 | -1.0221375 | 2.80174467 | -2.4197709 | 0.02801411 | 0.13191539 | -3.9519497 |
| TANK | -0.7919325 | 4.627088 | -2.4141455 | 0.02832872 | 0.13191539 | -3.9622009 |
| CXCL11 | -2.0500225 | 2.90605 | -2.413641 | 0.02864651 | 0.13191539 | -3.9677827 |
| GBP2 | -0.56104 | 1.67958067 | -2.4092981 | 0.02860251 | 0.13191539 | -3.9710259 |
| PTN | 1.5367325 | 6.24665933 | 2.39360117 | 0.02950629 | 0.13421765 | -3.9995483 |
| PPP1R9B | -0.9466425 | 5.05223533 | -2.3770644 | 0.03048751 | 0.13701016 | -4.0295055 |
| MMP12 | -2.552585 | 7.85513533 | -2.3596172 | 0.03185833 | 0.14145308 | -4.0652311 |
| STAMBP | -1.0364975 | 8.47873933 | -2.3472969 | 0.03233164 | 0.14145308 | -4.0831903 |
| Gal.9 | -0.9726875 | 6.844904 | -2.335429 | 0.03309567 | 0.14145308 | -4.1045056 |
| CLM.1 | -0.98318 | 4.38284533 | -2.3343129 | 0.03316839 | 0.14145308 | -4.1065076 |
| HAGH | 0.751815 | 2.238034 | 2.33119527 | 0.03337231 | 0.14145308 | -4.1120973 |
| MIC.A.B | -1.1330425 | 1.585832 | -2.3100483 | 0.03478682 | 0.14538589 | -4.1499189 |
| MATN3 | -1.251795 | 6.25952667 | -2.3057705 | 0.03507971 | 0.14538589 | -4.1575496 |
| VEGFA.1 | -1.0365825 | 8.119742 | -2.2818675 | 0.03675915 | 0.15067211 | -4.2000621 |
| HMOX2 | -1.0647925 | 4.374058 | -2.2409723 | 0.0398076 | 0.15621881 | -4.2722892 |
| IL33.1 | 1.4753775 | 3.428026 | 2.23895194 | 0.03996414 | 0.15621881 | -4.2758405 |
| IL18.1 | -0.972795 | 9.322184 | -2.2384869 | 0.04000025 | 0.15621881 | -4.2766578 |
| CXCL10.1 | -2.3381825 | 8.93675333 | -2.2307528 | 0.04093762 | 0.15621881 | -4.2935009 |
| CDSN | 0.5435625 | 8.67206533 | 2.22404532 | 0.04113693 | 0.15621881 | -4.3019928 |
| CCL20.1 | -2.3768575 | 3.701202 | -2.2241723 | 0.0414606 | 0.15621881 | -4.3049898 |
| ARNT | -1.5485875 | 1.508608 | -2.2207192 | 0.04173751 | 0.15621881 | -4.3110119 |
| CTSS | -0.653605 | 5.48802333 | -2.218889 | 0.04155 | 0.15621881 | -4.3110184 |
| GALNT3 | -0.544565 | 2.08188467 | -2.2147825 | 0.04188172 | 0.15621881 | -4.3181989 |
| CADM3 | 0.9896525 | 2.79597 | 2.16948304 | 0.04570741 | 0.1642759 | -4.3969487 |
| SRPK2 | -1.24421 | 6.01826333 | -2.1673367 | 0.04589645 | 0.1642759 | -4.4006588 |
| WWP2 | -0.6097325 | 6.96952667 | -2.1653445 | 0.04607256 | 0.1642759 | -4.4041008 |
| CXCL5.1 | 0.7916125 | 4.157954 | 2.16266363 | 0.04631054 | 0.1642759 | -4.4087299 |
| TWEAK | 1.08506 | 7.93084067 | 2.16018905 | 0.04653121 | 0.1642759 | -4.4130001 |
| IRF9 | -1.4001 | 4.17827867 | -2.1584787 | 0.0466843 | 0.1642759 | -4.41595 |
| ZBTB16 | -1.187685 | 4.34092133 | -2.1366401 | 0.04868027 | 0.16903101 | -4.4535063 |
| IFN.gamma | -1.944325 | 3.37874467 | -2.1376173 | 0.04894195 | 0.16903101 | -4.4544837 |
| CD8A.1 | -0.837885 | 5.953074 | -2.1017634 | 0.05203126 | 0.17805192 | -4.513055 |
| TDGF1 | -0.94958 | 0.83736933 | -2.0924889 | 0.05295732 | 0.17957347 | -4.5287999 |
| X4E.BP1 | -0.5676225 | 10.7715627 | -2.0676481 | 0.05551252 | 0.18654209 | -4.5707801 |
| DFFA | -0.82753 | 9.83476133 | -2.0317667 | 0.05940249 | 0.19605615 | -4.6309186 |
| CXCL10 | -2.1297325 | 7.92039067 | -2.0284347 | 0.06015027 | 0.19605615 | -4.6384944 |
| ATP6V1F | -0.56006 | 3.40652267 | -2.0207143 | 0.06064975 | 0.19605615 | -4.6493213 |
| PD.L1 | -0.591265 | 2.51579867 | -2.0192081 | 0.06082156 | 0.19605615 | -4.6518248 |
| IL32 | -0.71092 | 1.84879 | -2.0175621 | 0.06100982 | 0.19605615 | -4.6545593 |
| CCL20 | -2.219235 | 3.52175933 | -2.0141834 | 0.06177473 | 0.19605615 | -4.6621143 |
| CLEC4A | -0.532875 | 2.291828 | -2.0087808 | 0.06202312 | 0.19605615 | -4.669126 |
| RGMB | 0.6807475 | 4.542826 | 1.99969076 | 0.06308813 | 0.19774682 | -4.684166 |
| CLM.6 | -0.6558725 | 4.09243 | -1.9762786 | 0.06590783 | 0.2048635 | -4.7227182 |
| GDNF | -0.44455 | 1.40385667 | -1.9648542 | 0.06732469 | 0.20753808 | -4.7414328 |
| TWEAK.1 | 0.88329 | 9.13287733 | 1.96017109 | 0.06791339 | 0.20763684 | -4.7490856 |
| CD40.1 | -0.742275 | 9.739654 | -1.9419829 | 0.07024405 | 0.21058387 | -4.7787038 |
| CSF.1.1 | -0.79363 | 8.16635733 | -1.9405374 | 0.07043234 | 0.21058387 | -4.7810506 |
| BMP.4 | -0.579585 | 2.81864667 | -1.9376159 | 0.07081426 | 0.21058387 | -4.7857904 |
| IFNLR1 | -0.5679025 | 1.92911333 | -1.9341443 | 0.07127052 | 0.21058387 | -4.791417 |
| ADAM.23 | 0.5898025 | 1.679288 | 1.93089352 | 0.07170014 | 0.21058387 | -4.7966802 |
| ADAM.22 | 0.69582 | 3.01796067 | 1.92381529 | 0.07264367 | 0.21168818 | -4.8081216 |
| GZMA.1 | -0.980855 | 3.469452 | -1.902833 | 0.0755064 | 0.2183247 | -4.8418868 |
| LILRB4 | -0.56534 | 1.712382 | -1.8960289 | 0.07645618 | 0.21937042 | -4.8527872 |
| ADGRG1 | -0.59212 | 1.63717267 | -1.8906329 | 0.07721699 | 0.21986211 | -4.8614146 |
| SMOC1 | 0.71719 | 3.76668 | 1.86009926 | 0.08165075 | 0.22930173 | -4.9099435 |
| GDNFR.alpha.3 | 0.5963175 | 4.39912267 | 1.85935403 | 0.08176174 | 0.22930173 | -4.9111218 |
| CLEC4C | -0.62568 | 2.003412 | -1.8494117 | 0.08325542 | 0.23174829 | -4.9268125 |
| VEGFA | -0.8135125 | 5.94944533 | -1.8407873 | 0.08457067 | 0.23366562 | -4.9403798 |
| TRIM5 | -0.77801 | 4.681386 | -1.8315416 | 0.08600112 | 0.23452403 | -4.9548798 |
| IRAK1 | -0.7921225 | 4.34604867 | -1.8306586 | 0.08613885 | 0.23452403 | -4.9562622 |
| STC1 | -0.9008375 | 5.626566 | -1.8257644 | 0.08690579 | 0.23489753 | -4.9639164 |
| sFRP.3 | 0.8823975 | 5.17201667 | 1.81490985 | 0.08862833 | 0.23782999 | -4.9808453 |
| CTSC | -0.614345 | 3.43494333 | -1.8051156 | 0.09020845 | 0.24034109 | -4.9960646 |
| CXCL9.1 | -1.5285775 | 6.21711933 | -1.793902 | 0.09245579 | 0.24264973 | -5.0143806 |
| TNFRSF9 | -0.726635 | 4.48321267 | -1.7903725 | 0.09263382 | 0.24264973 | -5.0188728 |
| RPS6KB1 | -0.67616 | 4.790132 | -1.7868426 | 0.09322299 | 0.24264973 | -5.0243157 |
| CCL11.1 | -0.637645 | 3.975568 | -1.7824203 | 0.09396575 | 0.24264973 | -5.0311246 |
| AXIN1 | -0.881165 | 4.36183867 | -1.7802767 | 0.09432764 | 0.24264973 | -5.034421 |
| TIE2 | 0.6276225 | 4.28769133 | 1.75205671 | 0.09920738 | 0.25321119 | -5.0775738 |
| NF2 | -0.92093 | 2.51296267 | -1.7375371 | 0.10180315 | 0.25321119 | -5.0995978 |
| IL7.1 | -0.4569275 | 2.60088733 | -1.7342232 | 0.10240385 | 0.25321119 | -5.1046072 |
| KPNA1 | -1.0381925 | -0.1835153 | -1.7308083 | 0.10302609 | 0.25321119 | -5.1097627 |
| IL18 | -0.8037825 | 9.10684733 | -1.7288421 | 0.10338584 | 0.25321119 | -5.1127279 |
| ITGA11 | 0.43767 | 1.63231133 | 1.72771442 | 0.10359267 | 0.25321119 | -5.1144275 |
| CNTN5 | 0.519225 | 2.09713067 | 1.72743483 | 0.103644 | 0.25321119 | -5.1148488 |
| CD5.1 | -0.6049325 | 2.632816 | -1.7262374 | 0.10386411 | 0.25321119 | -5.1166525 |
| CLEC10A | -0.607765 | 2.79907933 | -1.7221896 | 0.1046112 | 0.25337649 | -5.1227438 |
| MDGA1 | 0.441035 | 1.88388667 | 1.68863244 | 0.11098675 | 0.26391246 | -5.1728664 |
| IL33 | 0.626085 | 1.281152 | 1.6866338 | 0.11137689 | 0.26391246 | -5.1758304 |
| SCARA5 | 0.6091325 | 8.81008667 | 1.68637812 | 0.11142688 | 0.26391246 | -5.1762094 |
| ITGB6 | -0.490155 | 1.733256 | -1.6845172 | 0.11179134 | 0.26391246 | -5.1789667 |
| OSM | -1.0845 | 4.233262 | -1.6711574 | 0.1144382 | 0.26834322 | -5.1987001 |
| CXCL9 | -1.4448175 | 5.48359467 | -1.669895 | 0.11510701 | 0.26834322 | -5.2011034 |
| CD244.1 | -0.4348725 | 3.82984733 | -1.6503745 | 0.11866326 | 0.27491549 | -5.2291816 |
| ILKAP | -0.4947375 | 6.71198333 | -1.6279236 | 0.12337743 | 0.28407272 | -5.2618096 |
| CDH6 | 0.5581225 | 2.53549133 | 1.62345909 | 0.12433376 | 0.28429031 | -5.2682604 |
| CKAP4 | -0.6238625 | 3.90193667 | -1.6203834 | 0.12499627 | 0.28429031 | -5.2726971 |
| BACH1 | -0.7246675 | 5.27286133 | -1.6018856 | 0.12904479 | 0.29171943 | -5.299255 |
| FLRT2 | -0.6186675 | 4.86073667 | -1.5948109 | 0.13062246 | 0.2935071 | -5.3093549 |
| GZMA | -0.9137475 | 4.07848933 | -1.5860117 | 0.1326076 | 0.29368345 | -5.3218721 |
| IL4.1 | -1.02618 | 0.29341333 | -1.5837562 | 0.13312056 | 0.29368345 | -5.3250726 |
| CCL4.1 | -1.1271025 | 4.19437 | -1.5837241 | 0.13312786 | 0.29368345 | -5.3251181 |
| PMVK | -0.3496875 | 7.77638333 | -1.5792291 | 0.13415522 | 0.29368345 | -5.3314866 |
| FHIT | 0.453185 | 3.23354067 | 1.57712804 | 0.13463772 | 0.29368345 | -5.3344589 |
| PRTG | 0.425215 | 4.09204067 | 1.56740847 | 0.13688899 | 0.2968581 | -5.3481717 |
| NTRK3 | 0.4490025 | 4.084812 | 1.55036592 | 0.14091331 | 0.30289317 | -5.3720675 |
| HCLS1 | 0.9578575 | 4.53235 | 1.54876672 | 0.14129601 | 0.30289317 | -5.3743001 |
| CXCL6 | -0.6672275 | 4.07502 | -1.5453634 | 0.14211337 | 0.30290449 | -5.3790456 |
| PGF | -0.618715 | 6.81734 | -1.5307311 | 0.14567298 | 0.3087274 | -5.3993615 |
| TPPP3 | -0.30676 | 7.50903933 | -1.5221849 | 0.14778646 | 0.311437 | -5.4111616 |
| CD40 | -0.6066375 | 7.59228333 | -1.5063654 | 0.15176646 | 0.31802747 | -5.4328755 |
| TRANCE | -0.5923725 | 3.87758067 | -1.4865014 | 0.15689039 | 0.32692803 | -5.4599018 |
| Gal.8 | -0.5040625 | 8.547786 | -1.4755316 | 0.15978124 | 0.33110223 | -5.474712 |
| TNFRSF4 | -0.649845 | 4.02166933 | -1.4604211 | 0.16383548 | 0.33762781 | -5.4949773 |
| ADA.1 | -0.391995 | 5.809828 | -1.449486 | 0.16682216 | 0.34041969 | -5.5095444 |
| TRAIL.1 | -0.6619575 | 6.236228 | -1.4487835 | 0.16701556 | 0.34041969 | -5.5104773 |
| CSF.1 | -0.62558 | 7.43004867 | -1.4411166 | 0.16913829 | 0.34287272 | -5.5206373 |
| TGF.alpha | -0.55534 | 5.877876 | -1.4189594 | 0.175398 | 0.35266543 | -5.5497682 |
| TNFSF14.1 | -0.6190725 | 2.51072733 | -1.4099591 | 0.17799428 | 0.35266543 | -5.5615022 |
| SIRT2 | -0.943895 | 7.48034933 | -1.4038879 | 0.17976329 | 0.35266543 | -5.5693849 |
| CD8A | -0.635615 | 5.27410933 | -1.4030498 | 0.18000864 | 0.35266543 | -5.5704711 |
| CCL11 | -0.573425 | 4.15165733 | -1.4019063 | 0.1803438 | 0.35266543 | -5.5719522 |
| GM.CSF.R.alpha | -0.6137175 | 4.516196 | -1.4013944 | 0.18049401 | 0.35266543 | -5.572615 |
| EFNA4 | -0.40167 | 3.80227467 | -1.4010763 | 0.18058739 | 0.35266543 | -5.5730267 |
| CCL4 | -1.0050775 | 4.110052 | -1.3952935 | 0.18229201 | 0.35347685 | -5.5804989 |
| IL8.1 | -1.391305 | 7.95293133 | -1.3914165 | 0.18384587 | 0.35347685 | -5.5854406 |
| IMPA1 | -0.3278675 | 2.88716067 | -1.3905402 | 0.18370294 | 0.35347685 | -5.5866229 |
| SLAMF1 | -0.32516 | 1.398912 | -1.3596223 | 0.19309805 | 0.36936191 | -5.6260589 |
| TMPRSS5 | 0.29913 | 1.53344467 | 1.35605059 | 0.19420793 | 0.36947025 | -5.6305698 |
| DAPP1 | -0.7897075 | 4.694372 | -1.3530774 | 0.19513576 | 0.36947025 | -5.6343178 |
| Dkk.4 | -0.520375 | 2.18476733 | -1.3415105 | 0.1987793 | 0.37446807 | -5.6488371 |
| SH2D1A | -0.6292625 | 1.72601067 | -1.3368897 | 0.20025003 | 0.37534301 | -5.6546099 |
| DCTN1 | -0.441745 | 8.55627467 | -1.3331437 | 0.20144867 | 0.37570177 | -5.6592781 |
| CD200R1 | -0.3878825 | 2.64480667 | -1.3167058 | 0.20677656 | 0.38371969 | -5.67964 |
| ST1A1 | 0.63169 | 1.94166867 | 1.28220783 | 0.21832249 | 0.40314004 | -5.7217157 |
| CPA2 | -0.6092025 | 5.90223067 | -1.2780397 | 0.21975131 | 0.40377949 | -5.7267385 |
| IL.12B | -0.8781175 | 4.95215467 | -1.2703172 | 0.222418 | 0.4061653 | -5.7360097 |
| CD5 | -0.5140425 | 2.975118 | -1.2679871 | 0.22322758 | 0.4061653 | -5.738798 |
| IL.18R1 | -0.4435275 | 5.37206667 | -1.2515064 | 0.22901994 | 0.41468174 | -5.7584016 |
| NCR1 | -0.29111 | 1.32152067 | -1.2437758 | 0.23177712 | 0.41764669 | -5.7675251 |
| G.CSF | -0.51364 | 1.99874267 | -1.2395666 | 0.23328919 | 0.41835033 | -5.7724732 |
| CD83 | -0.3690775 | 1.76900267 | -1.2342985 | 0.23519245 | 0.41974538 | -5.7786468 |
| IL12.1 | -0.84867 | 6.298844 | -1.2311933 | 0.23631997 | 0.41974927 | -5.7822757 |
| Alpha.2.MRAP | -0.76115 | 6.58946467 | -1.2117262 | 0.24348414 | 0.4297285 | -5.8048543 |
| ING1 | -0.561705 | 4.49426067 | -1.2096916 | 0.24424247 | 0.4297285 | -5.807197 |
| CD83.1 | -0.3742675 | 1.63380067 | -1.1900391 | 0.25166121 | 0.4406525 | -5.8296573 |
| IL12 | -0.879675 | 5.21435733 | -1.1870252 | 0.25281403 | 0.4406525 | -5.8330747 |
| EIF4G1 | -0.3704825 | 7.26126933 | -1.15251 | 0.26630528 | 0.4620087 | -5.8716952 |
| NTF4 | 0.2478475 | 1.66850867 | 1.147282 | 0.26839542 | 0.46347912 | -5.8774616 |
| IL8 | -1.127635 | 7.517286 | -1.1298639 | 0.27580091 | 0.47407253 | -5.8962304 |
| Flt3L | -0.5027375 | 9.62566933 | -1.1254227 | 0.27726863 | 0.47440916 | -5.9013322 |
| CD27 | -0.373955 | 5.80362133 | -1.1219614 | 0.27869356 | 0.47466986 | -5.9050763 |
| EDAR | -0.2578775 | 1.516034 | -1.1087157 | 0.28419694 | 0.481843 | -5.9193137 |
| CST5 | -0.3025125 | 4.336758 | -1.1054426 | 0.28556922 | 0.48197882 | -5.9228097 |
| CRADD | -0.3567525 | 7.313446 | -1.0982014 | 0.28862262 | 0.4840736 | -5.9305126 |
| ARG1 | 0.626125 | 5.540088 | 1.09635292 | 0.28940593 | 0.4840736 | -5.9324721 |
| COL4A3BP | -0.6057525 | 3.175458 | -1.0840735 | 0.29464933 | 0.49064375 | -5.9454167 |
| GGT5 | 0.23683 | 2.42460733 | 1.06400751 | 0.30336739 | 0.50291571 | -5.9663 |
| CPM | 0.4645975 | 6.185972 | 1.05854825 | 0.30577153 | 0.50465832 | -5.9719233 |
| IL4 | -0.552675 | -0.077628 | -1.0304538 | 0.31836287 | 0.5231249 | -6.0004658 |
| N.CDase | -0.4971175 | 0.78788733 | -1.0235594 | 0.32150901 | 0.52597745 | -6.007368 |
| LAP.TGF.beta.1.1 | -0.426465 | 4.826524 | -1.0184998 | 0.32383204 | 0.52746441 | -6.0124077 |
| FGFR2 | 0.2621125 | 2.368186 | 1.01335596 | 0.32620598 | 0.52902101 | -6.0175089 |
| NTRK2 | 0.27617 | 4.32928267 | 1.00023055 | 0.33231971 | 0.53660282 | -6.0304229 |
| TRAIL | -0.4799875 | 6.105954 | -0.9909195 | 0.33670569 | 0.53721342 | -6.0394943 |
| HNMT | 0.483705 | 11.9747627 | 0.99028196 | 0.3370075 | 0.53721342 | -6.0401127 |
| SPRY2 | -0.29615 | 1.72181133 | -0.9893617 | 0.33744349 | 0.53721342 | -6.0410048 |
| EN.RAGE | -0.3197975 | 2.36032467 | -0.9872217 | 0.33845886 | 0.53721342 | -6.0430763 |
| CCL25 | 0.3650675 | 3.60056667 | 0.9811474 | 0.34135265 | 0.53834113 | -6.0489346 |
| RBKS | -0.2499375 | 7.41382267 | -0.9761644 | 0.34373948 | 0.53834113 | -6.0537166 |
| TNFSF14 | -0.4614375 | 2.37373733 | -0.9744657 | 0.34455584 | 0.53834113 | -6.0553419 |
| WFIKKN1 | 0.2316775 | 1.585504 | 0.97366221 | 0.34494244 | 0.53834113 | -6.0561098 |
| CD63 | -0.4773625 | 2.07229733 | -0.9591375 | 0.35198346 | 0.54704095 | -6.0698942 |
| CAIX | -0.37213 | 8.02969933 | -0.9300851 | 0.36636474 | 0.56603031 | -6.0969129 |
| VEGFR.2 | 0.4391025 | 5.146016 | 0.92834875 | 0.36723682 | 0.56603031 | -6.0985042 |
| DDR1 | -0.2691925 | 7.80842267 | -0.9248952 | 0.36897559 | 0.56636994 | -6.1016613 |
| HEXIM1 | -0.475245 | 8.46514133 | -0.9191562 | 0.37187745 | 0.5684848 | -6.1068845 |
| NT.3 | -0.22291 | 1.40743 | -0.9011563 | 0.38107943 | 0.58017398 | -6.1230775 |
| IL7 | -0.3344075 | 3.40738267 | -0.8905803 | 0.38655706 | 0.58415868 | -6.1324573 |
| TNFRSF12A.1 | -0.2693275 | 2.19654333 | -0.8900581 | 0.38682894 | 0.58415868 | -6.1329179 |
| MILR1 | -0.2367925 | 1.88823867 | -0.8751469 | 0.39464475 | 0.59355843 | -6.1459658 |
| NCAN | -0.38123 | 4.33208667 | -0.8706159 | 0.39704034 | 0.59430216 | -6.1498911 |
| CD6 | -0.34033 | 2.39212333 | -0.8649524 | 0.40004823 | 0.59430216 | -6.1547716 |
| CD28 | -0.329115 | 1.172156 | -0.8614286 | 0.40192725 | 0.59430216 | -6.1577935 |
| EIF4B | -0.205085 | 8.230958 | -0.8588774 | 0.40329129 | 0.59430216 | -6.1599744 |
| PLA2G10 | 0.2693425 | 1.90714533 | 0.85707562 | 0.4042565 | 0.59430216 | -6.1615112 |
| SRP14 | -0.5025025 | 5.842748 | -0.8556366 | 0.40502844 | 0.59430216 | -6.1627364 |
| ADA | -0.2508525 | 6.40945533 | -0.8532843 | 0.40629236 | 0.59430216 | -6.1647351 |
| SIGLEC1 | -0.2950025 | 4.66747 | -0.8462992 | 0.41006087 | 0.59747151 | -6.170641 |
| PVR | 0.2694175 | 5.16264267 | 0.81918315 | 0.42490504 | 0.61012601 | -6.193147 |
| SCARF2 | 0.27449 | 4.19470933 | 0.81802582 | 0.42554619 | 0.61012601 | -6.1940926 |
| TNFB | -0.24868 | 3.425338 | -0.8179885 | 0.42556688 | 0.61012601 | -6.1941231 |
| LAMP3.1 | -0.2909875 | 2.109474 | -0.8161031 | 0.4266127 | 0.61012601 | -6.1956609 |
| PDGF.subunit.B | 0.24111 | 2.188612 | 0.81554137 | 0.42692464 | 0.61012601 | -6.1961185 |
| PRDX1 | -0.256485 | 7.59865133 | -0.8062553 | 0.43210207 | 0.61516822 | -6.2036405 |
| CD70 | -0.266465 | 2.43169333 | -0.7887932 | 0.44194556 | 0.62553322 | -6.2175707 |
| BST2 | -0.185155 | 1.95245133 | -0.7874005 | 0.44273665 | 0.62553322 | -6.2186696 |
| EGF | 0.36988 | 2.45633 | 0.7818305 | 0.44590949 | 0.62719677 | -6.2230467 |
| GCP5 | 0.299615 | 2.11501333 | 0.77943734 | 0.44727705 | 0.62719677 | -6.2249185 |
| IL10.2 | -0.572815 | 5.567268 | -0.7598845 | 0.45854818 | 0.64059353 | -6.2400126 |
| PHOSPHO1 | 0.1821725 | 4.99262733 | 0.74950569 | 0.46460143 | 0.6462958 | -6.2478802 |
| UNC5C | -0.3538225 | 1.95645533 | -0.746957 | 0.46609536 | 0.6462958 | -6.2497969 |
| CDH15 | 0.318905 | 2.76480867 | 0.73840009 | 0.47113232 | 0.65086057 | -6.2561872 |
| PRDX5 | 0.18914 | 8.30354467 | 0.73111277 | 0.47544784 | 0.65439868 | -6.2615754 |
| AKT1S1 | -0.3453825 | 6.016606 | -0.7275147 | 0.47758738 | 0.65492681 | -6.2642174 |
| LAP.TGF.beta.1 | -0.3433525 | 5.53878067 | -0.7114371 | 0.48721788 | 0.66568597 | -6.275874 |
| ANGPT2 | 0.254295 | 3.14252067 | 0.70258537 | 0.4925689 | 0.67054088 | -6.2821875 |
| Gal.1 | 0.259315 | 6.65992467 | 0.69309033 | 0.49834708 | 0.67593985 | -6.2888775 |
| CD244 | -0.2167325 | 2.91399733 | -0.6879971 | 0.50146281 | 0.67770155 | -6.2924308 |
| SCF | 0.2127375 | 7.066144 | 0.68287784 | 0.50460579 | 0.67839815 | -6.2959774 |
| NOS3 | 0.21023 | 0.94918067 | 0.68087866 | 0.5058363 | 0.67839815 | -6.2973557 |
| FASLG | -0.218875 | 3.44532333 | -0.6760964 | 0.50878684 | 0.67839815 | -6.3006372 |
| CCL28 | -0.11269 | 2.198728 | -0.6752481 | 0.5093112 | 0.67839815 | -6.301217 |
| SPOCK1 | -0.2095525 | 1.93733667 | -0.6724034 | 0.51107206 | 0.67839815 | -6.3031564 |
| FAM3B | 0.2831475 | 2.46356933 | 0.66554705 | 0.51533034 | 0.68162488 | -6.3077989 |
| IL10 | -0.506815 | 5.514914 | -0.6557436 | 0.52145393 | 0.68728733 | -6.3143588 |
| DRAXIN | 0.2673175 | 2.67732933 | 0.64049564 | 0.53105957 | 0.69748317 | -6.3243789 |
| NCR1.1 | -0.16217 | 1.73345267 | -0.6348917 | 0.53461444 | 0.69968837 | -6.3280053 |
| CX3CL1 | -0.2797875 | 4.38999667 | -0.6171908 | 0.54592911 | 0.71064891 | -6.339261 |
| TNFRSF12A | -0.1934425 | 2.01245533 | -0.6158374 | 0.54679956 | 0.71064891 | -6.3401092 |
| MANF | -0.4545475 | 6.13890333 | -0.6097225 | 0.55074175 | 0.71328706 | -6.3439191 |
| IL10.1 | -0.466505 | 4.491188 | -0.5981919 | 0.5582735 | 0.71941873 | -6.350945 |
| GZMH | -0.25865 | 1.446484 | -0.5963937 | 0.55938766 | 0.71941873 | -6.3520982 |
| LAMP3 | -0.15761 | 1.994154 | -0.5931285 | 0.56151663 | 0.71941873 | -6.3540755 |
| RGMA | 0.1953725 | 8.53068133 | 0.58924934 | 0.56405153 | 0.71941873 | -6.3564111 |
| THY.1 | -0.2304475 | 9.82812133 | -0.5876173 | 0.56511981 | 0.71941873 | -6.3573894 |
| CLEC1B | 0.16928 | 5.10061133 | 0.58284348 | 0.56825072 | 0.72066438 | -6.360236 |
| MMP7 | 0.2119125 | 11.6912907 | 0.5802394 | 0.56996244 | 0.72066438 | -6.3617795 |
| GPNMB | -0.12178 | 6.176242 | -0.5448293 | 0.59350263 | 0.74789352 | -6.3821088 |
| EZR | -0.10546 | 6.77741133 | -0.5379842 | 0.59810901 | 0.75116048 | -6.3858967 |
| NEFL | 0.3813475 | 2.02421067 | 0.53265654 | 0.60186251 | 0.75333798 | -6.3886557 |
| MAPT | 0.529745 | 2.890688 | 0.52525614 | 0.60687488 | 0.75707134 | -6.3926637 |
| MCP.2.1 | -0.248645 | 7.88289 | -0.5149883 | 0.61371311 | 0.76304997 | -6.3982839 |
| EDA2R | -0.185415 | 3.445968 | -0.5056485 | 0.62010656 | 0.76843769 | -6.4031657 |
| DNER | -0.1771575 | 6.23319067 | -0.4897205 | 0.63108281 | 0.77870897 | -6.4112916 |
| SMPD1 | 0.1446475 | 2.77664267 | 0.48757168 | 0.63257056 | 0.77870897 | -6.4123686 |
| IFI30 | -0.1145375 | 4.55556867 | -0.4738805 | 0.64208775 | 0.78651614 | -6.4191225 |
| KLRD1 | -0.138635 | 3.21115 | -0.4723871 | 0.64312982 | 0.78651614 | -6.4198479 |
| TPSAB1 | 0.19742 | 5.57535867 | 0.46430095 | 0.64878551 | 0.79083985 | -6.423737 |
| CD200 | 0.1291825 | 3.91090667 | 0.44967497 | 0.65907187 | 0.79753472 | -6.4306055 |
| IRAK4 | -0.2004075 | 6.409184 | -0.4491034 | 0.6594753 | 0.79753472 | -6.4308696 |
| TNF | 0.39496 | 3.19042467 | 0.4475613 | 0.6606923 | 0.79753472 | -6.4314632 |
| LIF | -0.1987625 | 2.10812333 | -0.4298235 | 0.67314696 | 0.80893145 | -6.4395851 |
| KLRD1.1 | -0.1225025 | 2.63913067 | -0.4279654 | 0.67447099 | 0.80893145 | -6.4404054 |
| BCAN | -0.2175675 | 5.64780667 | -0.4144984 | 0.6840998 | 0.81574352 | -6.4462466 |
| MCP.2 | -0.20438 | 6.07298733 | -0.4138283 | 0.68458036 | 0.81574352 | -6.4465324 |
| IL.22.RA1 | -0.1394375 | 2.56774067 | -0.410859 | 0.6867117 | 0.81574352 | -6.4477938 |
| CRTAM.1 | 0.13368 | 2.41204933 | 0.40278511 | 0.69252084 | 0.8175782 | -6.4511785 |
| NPM1 | 0.2673 | 8.003858 | 0.40174442 | 0.69327107 | 0.8175782 | -6.45161 |
| MSR1 | -0.2075625 | 4.52127467 | -0.3987385 | 0.69543984 | 0.8175782 | -6.4528502 |
| CD4 | -0.134275 | 2.14838467 | -0.3957425 | 0.69760427 | 0.8175782 | -6.4540772 |
| IKZF2 | -0.1493075 | 2.40361733 | -0.3935143 | 0.69921568 | 0.8175782 | -6.4549839 |
| HO.1 | 0.23586 | 10.356456 | 0.38223556 | 0.70739528 | 0.82262103 | -6.4594963 |
| CCL3.1 | -0.2312225 | 5.966756 | -0.3809956 | 0.70829679 | 0.82262103 | -6.4599846 |
| ROBO2 | -0.128175 | 3.34497333 | -0.3776782 | 0.71071101 | 0.82262103 | -6.4612832 |
| IL.10RB | -0.110785 | 3.71566467 | -0.3750971 | 0.71259157 | 0.82262103 | -6.4622858 |
| IL.20RA | 0.09541 | 2.15062933 | 0.3724043 | 0.71455553 | 0.82262103 | -6.4633246 |
| LAYN | -0.1146975 | 5.817376 | -0.3395498 | 0.73868129 | 0.84713068 | -6.4754064 |
| NDRG1 | -0.0632925 | 3.38654333 | -0.3372411 | 0.74038767 | 0.84713068 | -6.4762141 |
| FCRL2 | 0.07008 | 1.75438133 | 0.30433726 | 0.7648553 | 0.86997828 | -6.4871347 |
| PLXNB3 | -0.05825 | 2.057812 | -0.3041155 | 0.76502112 | 0.86997828 | -6.4872046 |
| LXN | 0.073125 | 1.73456867 | 0.29423227 | 0.77242291 | 0.87572567 | -6.4902664 |
| CCL19.1 | -0.2491975 | 7.30787933 | -0.2825214 | 0.78130023 | 0.88223932 | -6.4937147 |
| FGF.19 | 0.126605 | 5.807234 | 0.27781696 | 0.78476678 | 0.88223932 | -6.4951308 |
| PTH1R | 0.09327 | 2.125702 | 0.27715749 | 0.78526395 | 0.88223932 | -6.4953205 |
| Nr.CAM | 0.0780325 | 7.043074 | 0.27147921 | 0.78954875 | 0.88438944 | -6.4969349 |
| TNFRSF21.1 | -0.0724175 | 6.83865733 | -0.2513702 | 0.80477829 | 0.89874941 | -6.5023861 |
| CD302 | -0.0875325 | 3.63938867 | -0.2469602 | 0.80812929 | 0.89928044 | -6.503526 |
| ADGRB3 | 0.076505 | 3.03718 | 0.2444011 | 0.81007568 | 0.89928044 | -6.5041783 |
| DCN | 0.066 | 4.862266 | 0.23404015 | 0.81796895 | 0.90437744 | -6.5067502 |
| CD38 | 0.06846 | 2.66715267 | 0.23201222 | 0.81951629 | 0.90437744 | -6.5072407 |
| CRIP2 | -0.0441 | 9.43178733 | -0.228684 | 0.82205744 | 0.90450568 | -6.5080365 |
| NMNAT1 | 0.1106925 | 8.89081733 | 0.21976995 | 0.82887347 | 0.90932295 | -6.5101115 |
| CX3CL1.1 | -0.0835925 | 3.45324733 | -0.2133528 | 0.8337891 | 0.91203324 | -6.5115545 |
| CCL3 | -0.1284975 | 5.59784 | -0.2097978 | 0.83651532 | 0.9123398 | -6.5123356 |
| JAM.B | 0.0677125 | 6.909452 | 0.19529607 | 0.84765842 | 0.92179764 | -6.5153868 |
| TNFRSF21 | -0.0666475 | 6.33833267 | -0.1917903 | 0.85035738 | 0.92204448 | -6.5160918 |
| LY75 | -0.0553525 | 1.71931667 | -0.187647 | 0.85354961 | 0.9228232 | -6.5169086 |
| CXCL13 | 0.119325 | 5.51345333 | 0.17934991 | 0.85995001 | 0.92705593 | -6.5184909 |
| CD33 | 0.1048425 | 2.64840267 | 0.16315405 | 0.87247229 | 0.93483033 | -6.5213742 |
| CDH17 | 0.0577475 | 1.08910933 | 0.16287603 | 0.87268757 | 0.93483033 | -6.5214213 |
| GDF.8 | 0.0482525 | 2.21429933 | 0.16030301 | 0.87468039 | 0.93483033 | -6.5218536 |
| DCBLD2 | -0.0831575 | 5.307228 | -0.1558267 | 0.87814943 | 0.93585639 | -6.5225894 |
| SFRP1 | 0.0835975 | 10.6416153 | 0.13172524 | 0.8968697 | 0.95276754 | -6.5261936 |
| FCRL6 | -0.0397225 | 1.266898 | -0.1288255 | 0.89912647 | 0.95276754 | -6.5265867 |
| CCL27 | -0.02958 | 5.73406 | -0.1213991 | 0.90491031 | 0.95618001 | -6.5275534 |
| FGF.21 | 0.0633225 | 2.63970133 | 0.11514936 | 0.90978205 | 0.95861216 | -6.5283226 |
| NRP2 | 0.033935 | 6.77884867 | 0.10502165 | 0.91768452 | 0.96034134 | -6.5294829 |
| NBL1 | 0.023965 | 5.41685333 | 0.10332725 | 0.91900752 | 0.96034134 | -6.5296666 |
| PSIP1 | 0.0959775 | 6.02344933 | 0.10257883 | 0.91961919 | 0.96034134 | -6.5297399 |
| CARHSP1 | 0.0317575 | 3.31112933 | 0.09985211 | 0.92172171 | 0.96034134 | -6.5300341 |
| PDGF.R.alpha | 0.03763 | 5.07817733 | 0.09534376 | 0.92524436 | 0.96132631 | -6.5304921 |
| SKR3 | -0.0276625 | 5.62163867 | -0.090913 | 0.92870797 | 0.96224465 | -6.5309216 |
| CRTAM | -0.031365 | 2.515772 | -0.0797176 | 0.93746599 | 0.96862829 | -6.531916 |
| CLEC4D | -0.0240175 | 1.54126267 | -0.0652721 | 0.94877868 | 0.97113784 | -6.5330063 |
| SH2B3 | -0.033415 | 1.37384867 | -0.0652243 | 0.94881613 | 0.97113784 | -6.5330095 |
| ICOSLG | 0.018665 | 3.74165133 | 0.06324028 | 0.95037084 | 0.97113784 | -6.5331422 |
| EPHB6 | 0.02487 | 6.44665133 | 0.05954748 | 0.95326509 | 0.97113784 | -6.5333783 |
| MGMT | 0.01224 | 7.89820267 | 0.05556447 | 0.95638755 | 0.97113784 | -6.533617 |
| VWC2 | 0.0275975 | 3.91596933 | 0.05415475 | 0.95749287 | 0.97113784 | -6.5336976 |
| NAAA | -0.0243925 | 3.161468 | -0.0524053 | 0.95886466 | 0.97113784 | -6.5337946 |
| SCARB2 | 0.0131225 | 2.985386 | 0.04970171 | 0.96098495 | 0.97113784 | -6.5339384 |
| LEPR | -0.01408 | 1.593664 | -0.0467157 | 0.96332708 | 0.97113784 | -6.5340883 |
| RSPO1 | 0.01535 | 6.22276867 | 0.03337465 | 0.97379513 | 0.9790447 | -6.5346447 |
| CCL19 | -0.0239475 | 6.50520067 | -0.0270671 | 0.97875339 | 0.98138444 | -6.5348427 |
| SMOC2 | 0.011765 | 8.235928 | 0.02360514 | 0.98146406 | 0.98146406 | -6.5349345 |

**Supplementary Table 5: Differential protein expression as detected by a proteomic multiplex assay from suction blister fluid,** given as log2 fold change (logFC) of spontaneously healed atopic dermatitis (n=4) over active AD (n=4); adjusted p values were calculated using a linear mixed model with Benjamini-Hochberg correction for multiple testing.

| **p value** | **avg_logFC** | **pct.1** | **pct.2** | **Adjusted p value** | **Cluster** | **Gene** | **Cluster label** |
| --- | --- | --- | --- | --- | --- | --- | --- |
| 0 | 1.43749622 | 0.986 | 0.558 | 0 | 0 | MT1X | KC-4 |
| 0 | 1.36411049 | 0.995 | 0.412 | 0 | 0 | LY6D |  |
| 0 | 1.34898936 | 0.998 | 0.488 | 0 | 0 | SFN |  |
| 0 | 1.29148493 | 0.995 | 0.399 | 0 | 0 | DMKN |  |
| 0 | 1.18735688 | 0.972 | 0.353 | 0 | 0 | LGALS7B |  |
| 0 | 1.13082026 | 0.897 | 0.31 | 0 | 0 | LYPD3 |  |
| 0 | 1.10110712 | 0.983 | 0.397 | 0 | 0 | S100A14 |  |
| 0 | 1.06768484 | 0.969 | 0.389 | 0 | 0 | S100A16 |  |
| 3.80E-286 | 1.0479806 | 0.835 | 0.519 | 1.27E-281 | 0 | PHLDA2 |  |
| 6.26E-215 | 1.1566827 | 0.692 | 0.272 | 2.10E-210 | 0 | KRT16 |  |
| 0 | 2.64503123 | 0.811 | 0.041 | 0 | 1 | KRT15 | KC-2 |
| 0 | 2.36623289 | 0.772 | 0.115 | 0 | 1 | CYR61 |  |
| 0 | 1.9841961 | 0.996 | 0.401 | 0 | 1 | CXCL14 |  |
| 0 | 1.94674367 | 0.945 | 0.281 | 0 | 1 | DST |  |
| 0 | 1.8516858 | 0.478 | 0.042 | 0 | 1 | CCL2 |  |
| 0 | 1.70592024 | 0.998 | 0.588 | 0 | 1 | KRT14 |  |
| 0 | 1.69813649 | 0.712 | 0.064 | 0 | 1 | POSTN |  |
| 0 | 1.60351976 | 0.993 | 0.397 | 0 | 1 | S100A2 |  |
| 0 | 1.52343504 | 0.867 | 0.13 | 0 | 1 | COL17A1 |  |
| 0 | 1.43957998 | 0.883 | 0.331 | 0 | 1 | KRT17 |  |
| 0 | 2.22230101 | 0.948 | 0.148 | 0 | 2 | AIF1 | Mo/Ma |
| 0 | 2.02252335 | 0.954 | 0.165 | 0 | 2 | LYZ |  |
| 0 | 1.98680318 | 0.997 | 0.917 | 0 | 2 | FTL |  |
| 0 | 1.90263083 | 0.983 | 0.202 | 0 | 2 | TYROBP |  |
| 0 | 1.84695612 | 0.961 | 0.192 | 0 | 2 | FCER1G |  |
| 0 | 1.78060931 | 0.836 | 0.1 | 0 | 2 | MS4A6A |  |
| 0 | 1.76785758 | 0.931 | 0.255 | 0 | 2 | PLAUR |  |
| 0 | 1.75984812 | 0.416 | 0.029 | 0 | 2 | RNASE1 |  |
| 0 | 1.69077841 | 0.997 | 0.922 | 0 | 2 | FTH1 |  |
| 1.29E-186 | 1.57685316 | 0.199 | 0.021 | 4.33E-182 | 2 | APOBEC3A |  |
| 0 | 2.86523185 | 0.661 | 0.069 | 0 | 3 | MMP12 | DC/LC |
| 0 | 2.23096431 | 0.93 | 0.093 | 0 | 3 | FCER1A |  |
| 0 | 2.09824461 | 0.999 | 0.24 | 0 | 3 | HLA-DQA1 |  |
| 0 | 2.05176902 | 1 | 0.483 | 0 | 3 | CD74 |  |
| 0 | 2.03244372 | 0.999 | 0.311 | 0 | 3 | HLA-DQB1 |  |
| 0 | 1.98295156 | 1 | 0.34 | 0 | 3 | HLA-DPB1 |  |
| 0 | 1.9756385 | 1 | 0.389 | 0 | 3 | HLA-DRA |  |
| 0 | 1.97093579 | 0.887 | 0.057 | 0 | 3 | CD1A |  |
| 0 | 1.95702341 | 1 | 0.361 | 0 | 3 | HLA-DPA1 |  |
| 0 | 1.94136055 | 1 | 0.413 | 0 | 3 | HLA-DRB1 |  |
| 0 | 2.6631588 | 0.594 | 0.053 | 0 | 4 | KRT2 | KC-1 |
| 0 | 2.41997441 | 0.986 | 0.294 | 0 | 4 | KRTDAP |  |
| 0 | 2.08061595 | 0.998 | 0.415 | 0 | 4 | KRT1 |  |
| 0 | 2.05947928 | 0.999 | 0.766 | 0 | 4 | KRT10 |  |
| 0 | 1.82962814 | 0.913 | 0.19 | 0 | 4 | SBSN |  |
| 0 | 1.67731793 | 0.999 | 0.447 | 0 | 4 | DMKN |  |
| 0 | 1.60745464 | 0.779 | 0.166 | 0 | 4 | CALML5 |  |
| 0 | 1.4734462 | 0.978 | 0.403 | 0 | 4 | LGALS7B |  |
| 0 | 1.45396283 | 0.933 | 0.355 | 0 | 4 | LYPD3 |  |
| 2.19E-153 | 1.49898593 | 0.462 | 0.161 | 7.34E-149 | 4 | SPRR1B |  |
| 0 | 2.22128687 | 0.95 | 0.131 | 0 | 5 | IL32 | TC-1 |
| 0 | 1.68761748 | 0.843 | 0.184 | 0 | 5 | IL7R |  |
| 0 | 1.67831944 | 0.909 | 0.228 | 0 | 5 | ALOX5AP |  |
| 0 | 1.62121886 | 0.85 | 0.105 | 0 | 5 | TRAC |  |
| 0 | 1.60986267 | 0.786 | 0.09 | 0 | 5 | TRBC2 |  |
| 0 | 1.59292589 | 0.872 | 0.088 | 0 | 5 | CD3D |  |
| 0 | 1.58427736 | 0.823 | 0.094 | 0 | 5 | CD2 |  |
| 2.20E-242 | 1.60814576 | 0.688 | 0.173 | 7.39E-238 | 5 | LTB |  |
| 4.22E-228 | 1.84014087 | 0.313 | 0.011 | 1.41E-223 | 5 | IL13 |  |
| 1.20E-135 | 1.80160749 | 0.158 | 0.006 | 4.02E-131 | 5 | IL22 |  |
| 0 | 4.73286024 | 1 | 0.022 | 0 | 6 | DCT | MEL |
| 0 | 4.55843495 | 0.992 | 0.033 | 0 | 6 | TYRP1 |  |
| 0 | 4.51177925 | 0.999 | 0.046 | 0 | 6 | PMEL |  |
| 0 | 4.32271793 | 0.998 | 0.032 | 0 | 6 | MLANA |  |
| 0 | 3.95098109 | 0.999 | 0.12 | 0 | 6 | IGFBP7 |  |
| 0 | 2.83301122 | 0.996 | 0.212 | 0 | 6 | QPCT |  |
| 0 | 2.76727723 | 0.963 | 0.006 | 0 | 6 | APOD |  |
| 0 | 2.70475781 | 0.947 | 0.015 | 0 | 6 | MITF |  |
| 0 | 2.54648677 | 0.983 | 0.019 | 0 | 6 | GPM6B |  |
| 0 | 2.43297194 | 0.994 | 0.458 | 0 | 6 | CD59 |  |
| 0 | 3.01120996 | 0.715 | 0.023 | 0 | 7 | CCL5 | TC-2 |
| 0 | 2.31523761 | 0.766 | 0.043 | 0 | 7 | NKG7 |  |
| 0 | 2.08207163 | 0.561 | 0.032 | 0 | 7 | GZMA |  |
| 0 | 2.04764885 | 0.967 | 0.1 | 0 | 7 | CD3D |  |
| 0 | 1.97072281 | 0.987 | 0.147 | 0 | 7 | IL32 |  |
| 0 | 1.90530453 | 0.703 | 0.014 | 0 | 7 | CD8A |  |
| 0 | 1.74486867 | 0.869 | 0.12 | 0 | 7 | TRAC |  |
| 5.79E-296 | 1.86512583 | 0.673 | 0.053 | 1.94E-291 | 7 | GZMB |  |
| 1.23E-178 | 1.8189989 | 0.254 | 0.002 | 4.12E-174 | 7 | GZMK |  |
| 9.14E-130 | 1.92854252 | 0.39 | 0.045 | 3.06E-125 | 7 | GNLY |  |
| 5.00E312 | 1.25475833 | 0.92 | 0.231 | 1.68E-307 | 8 | PCNA | KC-5 |
| 2.90E-290 | 1.34661546 | 0.801 | 0.075 | 9.74E-286 | 8 | TK1 |  |
| 3.89E-247 | 1.13082626 | 0.762 | 0.065 | 1.30E-242 | 8 | PCLAF |  |
| 1.02E-245 | 1.09513042 | 0.946 | 0.436 | 3.42E-241 | 8 | DUT |  |
| 8.37E-223 | 1.09914145 | 0.994 | 0.796 | 2.81E-218 | 8 | H2AFZ |  |
| 6.79E-180 | 0.91158612 | 0.924 | 0.408 | 2.28E-175 | 8 | CENPX |  |
| 1.25E-155 | 0.92348749 | 0.896 | 0.274 | 4.18E-151 | 8 | CKS1B |  |
| 1.13E-142 | 1.07900924 | 0.929 | 0.277 | 3.79E-138 | 8 | STMN1 |  |
| 2.61E-123 | 0.89182351 | 0.9 | 0.368 | 8.76E-119 | 8 | HMGB2 |  |
| 1.70E-38 | 0.87884819 | 0.671 | 0.266 | 5.69E-34 | 8 | KRT6A |  |
| 0 | 2.50113082 | 0.904 | 0.464 | 0 | 9 | HIST1H4C | KC-6 |
| 0 | 1.91867319 | 0.986 | 0.266 | 0 | 9 | PTTG1 |  |
| 0 | 1.88634208 | 0.993 | 0.367 | 0 | 9 | HMGB2 |  |
| 0 | 1.86914473 | 0.988 | 0.277 | 0 | 9 | STMN1 |  |
| 0 | 1.81353188 | 0.888 | 0.022 | 0 | 9 | UBE2C |  |
| 0 | 1.67684489 | 0.921 | 0.064 | 0 | 9 | CENPF |  |
| 0 | 1.66761795 | 0.935 | 0.045 | 0 | 9 | NUSAP1 |  |
| 0 | 1.56462776 | 0.885 | 0.023 | 0 | 9 | TOP2A |  |
| 0 | 1.53900141 | 0.959 | 0.274 | 0 | 9 | CKS1B |  |
| 0 | 1.46843313 | 1 | 0.797 | 0 | 9 | H2AFZ |  |
| 1.84E-290 | 1.83817947 | 0.963 | 0.471 | 6.17E-286 | 10 | DSP | KC-3 |
| 6.44E-246 | 1.76634115 | 0.99 | 0.452 | 2.16E-241 | 10 | KRT1 |  |
| 3.54E-190 | 1.49967544 | 0.99 | 0.782 | 1.19E-185 | 10 | KRT10 |  |
| 2.08E-168 | 1.41879751 | 0.806 | 0.403 | 6.98E-164 | 10 | DSC3 |  |
| 4.17E-157 | 1.42341549 | 0.724 | 0.329 | 1.40E-152 | 10 | PKP1 |  |
| 3.53E-143 | 1.47525539 | 0.698 | 0.447 | 1.18E-138 | 10 | MTRNR2L12 |  |
| 6.24E-143 | 1.27083127 | 0.864 | 0.445 | 2.09E-138 | 10 | EMP2 |  |
| 2.15E-116 | 1.29441779 | 0.845 | 0.394 | 7.22E-112 | 10 | LYPD3 |  |
| 9.26E-91 | 1.42285566 | 0.546 | 0.282 | 3.11E-86 | 10 | MTRNR2L8 |  |
| 1.45E-73 | 1.40590543 | 0.593 | 0.306 | 4.87E-69 | 10 | HES1 |  |
| 0 | 3.0988333 | 0.984 | 0.106 | 0 | 11 | CTSW | NK |
| 0 | 2.83204921 | 0.918 | 0.014 | 0 | 11 | XCL1 |  |
| 0 | 2.40928855 | 0.815 | 0.006 | 0 | 11 | XCL2 |  |
| 0 | 2.09948426 | 0.726 | 0.013 | 0 | 11 | SPINK2 |  |
| 0 | 1.98305368 | 0.973 | 0.328 | 0 | 11 | TNFRSF18 |  |
| 0 | 1.75403156 | 0.709 | 0.009 | 0 | 11 | KRT86 |  |
| 3.63E-280 | 3.01957864 | 0.791 | 0.043 | 1.22E-275 | 11 | GNLY |  |
| 1.08E-254 | 1.56659466 | 0.97 | 0.502 | 3.61E-250 | 11 | FXYD5 |  |
| 1.71E-252 | 2.90317435 | 0.717 | 0.066 | 5.73E-248 | 11 | GZMB |  |
| 7.61E-72 | 2.94494097 | 0.193 | 0.007 | 2.55E-67 | 11 | CCL1 |  |
| 5.62E-261 | 1.75169951 | 0.958 | 0.271 | 1.88E-256 | 12 | PTTG1 | KC-7 |
| 8.17E-216 | 1.37735265 | 0.872 | 0.07 | 2.74E-211 | 12 | CENPF |  |
| 7.84E-200 | 1.2314806 | 0.988 | 0.694 | 2.63E-195 | 12 | HMGN2 |  |
| 3.33E-185 | 1.19846922 | 0.73 | 0.047 | 1.12E-180 | 12 | CDC20 |  |
| 3.72E-180 | 1.09314932 | 0.748 | 0.055 | 1.25E-175 | 12 | BIRC5 |  |
| 4.77E-158 | 1.31537662 | 1 | 0.487 | 1.60E-153 | 12 | KRT5 |  |
| 2.60E-80 | 1.06549248 | 0.917 | 0.327 | 8.70E-76 | 12 | KRT16 |  |
| 6.59E-72 | 1.115204 | 0.858 | 0.265 | 2.21E-67 | 12 | KRT6A |  |
| 1.32E-59 | 1.1171462 | 0.861 | 0.397 | 4.43E-55 | 12 | KRT17 |  |
| 3.40E-54 | 1.11939001 | 0.439 | 0.1 | 1.14E-49 | 12 | CLDN4 |  |
| 1.26E-243 | 2.09865125 | 0.975 | 0.137 | 4.24E-239 | 13 | TRAC | TREG |
| 5.59E-241 | 1.6599755 | 0.673 | 0.019 | 1.88E-236 | 13 | CTLA4 |  |
| 4.45E-228 | 1.67865948 | 0.762 | 0.034 | 1.49E-223 | 13 | TIGIT |  |
| 2.47E-206 | 1.63444414 | 0.722 | 0.031 | 8.30E-202 | 13 | CD27 |  |
| 6.71E-201 | 1.96489443 | 0.997 | 0.168 | 2.25E-196 | 13 | IL32 |  |
| 2.43E-186 | 1.70282186 | 0.969 | 0.123 | 8.16E-182 | 13 | CD3D |  |
| 1.65E-170 | 2.39963015 | 0.759 | 0.099 | 5.53E-166 | 13 | TNFRSF4 |  |
| 3.94E-164 | 1.96793134 | 0.877 | 0.121 | 1.32E-159 | 13 | TRBC2 |  |
| 1.17E-163 | 1.70342247 | 0.627 | 0.039 | 3.93E-159 | 13 | IL2RA |  |
| 1.03E-83 | 1.71704581 | 0.556 | 0.072 | 3.46E-79 | 13 | TRBC1 |  |
| 0 | 3.66717838 | 0.93 | 0.027 | 0 | 14 | CCL22 | DC-1 |
| 0 | 1.82561649 | 0.896 | 0.03 | 0 | 14 | LAMP3 |  |
| 4.75E-318 | 2.27848324 | 1 | 0.93 | 1.59E-313 | 14 | TXN |  |
| 1.20E-243 | 2.10481963 | 0.696 | 0.015 | 4.04E-239 | 14 | CCR7 |  |
| 3.00E-235 | 2.09189863 | 0.926 | 0.185 | 1.01E-230 | 14 | BASP1 |  |
| 1.47E-233 | 1.98710943 | 0.941 | 0.105 | 4.93E-229 | 14 | IL4I1 |  |
| 1.90E-197 | 3.30887891 | 0.852 | 0.09 | 6.38E-193 | 14 | CCL17 |  |
| 2.16E-165 | 2.05228154 | 0.781 | 0.099 | 7.24E-161 | 14 | CD1B |  |
| 1.03E-164 | 2.07784615 | 0.907 | 0.234 | 3.45E-160 | 14 | BIRC3 |  |
| 2.39E-77 | 1.80415283 | 0.778 | 0.216 | 8.01E-73 | 14 | C15orf48 |  |
| 6.92E-172 | 2.17013948 | 0.867 | 0.051 | 2.32E-167 | 16 | IRF8 | DC-2 |
| 1.54E-93 | 2.43377881 | 0.392 | 0.001 | 5.18E-89 | 16 | JCHAIN |  |
| 1.68E-87 | 1.60583726 | 0.567 | 0.094 | 5.63E-83 | 16 | C1orf54 |  |
| 5.27E-86 | 2.36821359 | 0.933 | 0.202 | 1.77E-81 | 16 | LTB |  |
| 3.65E-77 | 1.66681375 | 1 | 0.536 | 1.22E-72 | 16 | CD74 |  |
| 1.49E-60 | 1.61308374 | 1 | 0.408 | 4.99E-56 | 16 | HLA-DPB1 |  |
| 4.24E-57 | 1.82688626 | 0.817 | 0.23 | 1.42E-52 | 16 | CPVL |  |
| 1.52E-37 | 1.5855443 | 0.558 | 0.124 | 5.11E-33 | 16 | S100B |  |
| 5.84E-27 | 1.59419496 | 0.567 | 0.152 | 1.96E-22 | 16 | HLA-DRB5 |  |
| 9.00E-17 | 1.73822328 | 0.325 | 0.08 | 3.02E-12 | 16 | GZMB |  |
| 1.17E-111 | 2.67888276 | 1 | 0.107 | 3.92E-107 | 17 | CRABP2 | KC-8 |
| 3.14E-91 | 1.66155897 | 0.873 | 0.004 | 1.05E-86 | 17 | KRT23 |  |
| 1.16E-67 | 2.92010467 | 1 | 0.217 | 3.88E-63 | 17 | CALML5 |  |
| 3.93E-52 | 2.00616157 | 0.964 | 0.444 | 1.32E-47 | 17 | CSTA |  |
| 1.84E-48 | 2.14622877 | 0.982 | 0.539 | 6.18E-44 | 17 | FABP5 |  |
| 3.83E-44 | 1.55652187 | 1 | 0.255 | 1.28E-39 | 17 | DEFB1 |  |
| 1.18E-30 | 1.51190225 | 0.527 | 0.041 | 3.96E-26 | 17 | KRT6B |  |
| 3.56E-26 | 1.51853013 | 0.691 | 0.092 | 1.20E-21 | 17 | TM4SF1 |  |
| 3.70E-26 | 1.69166296 | 0.982 | 0.353 | 1.24E-21 | 17 | KRTDAP |  |
| 9.93E-20 | 1.91545879 | 0.673 | 0.186 | 3.33E-15 | 17 | SPRR1B |  |

**Supplementary Table 6: Top 10 differentially expressed genes** according to highest average log fold change (avg logFC) ordered by smallest adjusted p-value using logistic regression with Bonferroni correction for each cluster, as compared to the rest of the dataset in all sequenced skin cells (comparison spontaneously healed AD vs. chronic active AD). pct.1/2: The percentage of cells in which the feature is detected in the first/second group.

**Supplementary Table 7: See separate excel sheet.**

**Differentially expressed genes of single-cell RNA sequencing analyses,** comparing spontaneously healed atopic dermatitis with untreated, active AD for each cluster. Differential gene expression was defined by average log fold change (avg logFC) >⏐0.25⏐ and adjusted p-value<0.05, using logistic regression and Bonferroni correction. pct.1/2: The percentage of cells in which the feature is detected in the first/second group.
