# Supplementary material for: Spontaneously Resolved Atopic Dermatitis Shows Melanocyte and Immune Cell Activation Distinct From Healthy Control Skin
Source: Front Immunol. 2021 Feb 24;12:630892. doi: 10.3389/fimmu.2021.630892 (PMC7943477; doi:10.3389/fimmu.2021.630892)
Supplement: Supplementary Figure 3 — scRNA-seq map of skin cells from suction blister samples of spontaneously healed atopic dermatitis and active AD lesions. (A,B) UMAP of spontaneously healed atopic dermatitis patients (n = 4) and active AD samples (n = 4) according to similarity of their transcriptome, resulting in 17 different color-coded clusters. (C) Expression heat map of top 10 downregulated DEGs per cluster in spontaneously healed AD vs. active AD samples. Upregulation is indicated in yellow, downregulation in purple. AD, Atopic dermatitis; KC, Keratinocytes; Mo/Ma, Monocytes/Macrophages; LC, Langerhans cells; TC, T lymphocytes; MEL, Melanocytes; NK, NK cells; TREG, regulatory T lymphocytes; DC, Dendritic cells; UMAP, Uniform Manifold Approximation and Projection. [file Image_3.pdf]

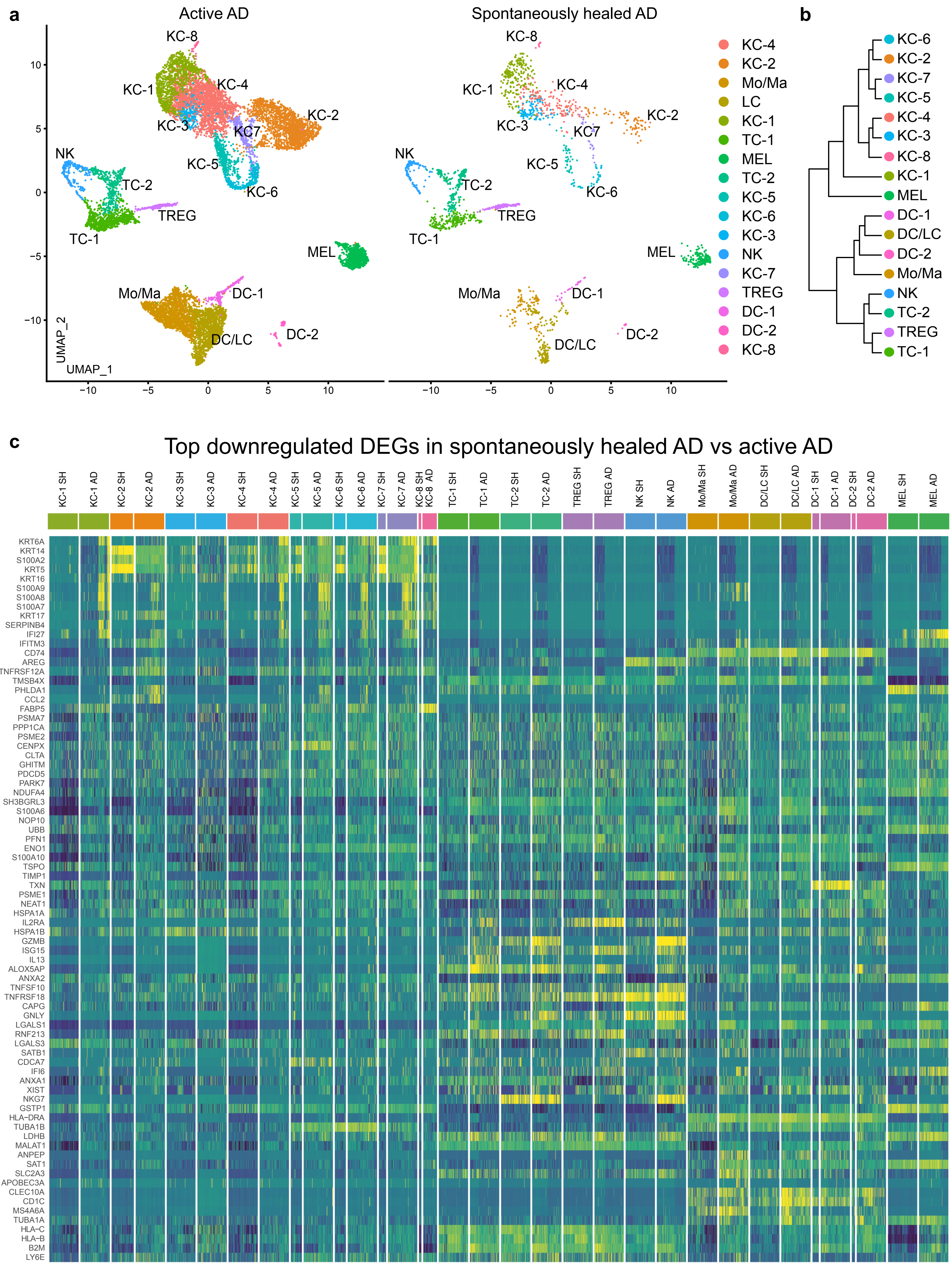
a
Active AD
Spontaneously healed AD
KC-8
KC-1
KC-4
KC-3
KC7
KC-2
KC-5
KC-6
NK
TC-2
TREG
TC-1
MEL
Mo/Ma
DC-1
DC/LC
DC-2
UMAP_2
UMAP_1
KC-4
KC-2
Mo/Ma
LC
KC-1
TC-1
MEL
TC-2
KC-5
KC-6
KC-3
NK
KC-7
TREG
DC-1
DC-2
KC-8
b
KC-6
KC-2
KC-7
KC-5
KC-4
KC-3
KC-8
KC-1
MEL
DC-1
DC/LC
DC-2
Mo/Ma
NK
TC-2
TREG
TC-1
c
Top downregulated DEGs in spontaneously healed AD vs active AD
KC-1 SH
KC-1 AD
KC-2 SH
KC-2 AD
KC-3 SH
KC-3 AD
KC-4 SH
KC-4 AD
KC-5 SH
KC-5 AD
KC-6 SH
KC-6 AD
KC-7 SH
KC-7 AD
KC-8 SH
KC-8 AD
TC-1 SH
TC-1 AD
TC-2 SH
TC-2 AD
TREG SH
TREG AD
NK SH
NK AD
Mo/Ma SH
Mo/Ma AD
DC/LC SH
DC/LC AD
DC-1 SH
DC-1 AD
DC-2 SH
DC-2 AD
MEL SH
MEL AD
KRT6A
KRT14
S100A2
KRT5
KRT16
S100A9
S100A8
S100A7
KRT17
SERPINB4
IFI27
IFITM3
CD74
AREG
TNFRSF12A
TMSB4X
PHLDA1
CCL2
FABP5
PSMA7
PPP1CA
PSME2
CENPX
CLTA
GHITM
PDCD5
PARK7
NDUFA4
SH3BGRL3
S100A6
NOP10
UBB
PFN1
ENO1
S100A10
TSPO
TIMP1
TXN
PSME1
NEAT1
HSPA1A
IL2RA
HSPA1B
GZMB
ISG15
IL13
ALOX5AP
ANXA2
TNFSF10
TNFRSF18
CAPG
GNLY
LGALS1
RNF213
LGALS3
SATB1
CDCA7
IFI6
ANXA1
XIST
NKG7
GSTP1
HLA-DRA
TUBA1B
LDHB
MALAT1
ANPEP
SAT1
SLC2A3
APOBEC3A
CLEC10A
CD1C
MS4A6A
TUBA1A
HLA-C
HLA-B
B2M
LY6E
